# Discovery of an Antiviral Electron Transfer Process to Create Catalytically Self‐Sufficient Viral Restriction Factors

**DOI:** 10.1002/anie.5423408

**Published:** 2026-07-21

**Authors:** Mengdi Wu, Nghi Thao Hoang, Deborah Grifagni, Meritxell Wu‐Lu, Yujie Sheng, Theo Situmorang, Pei‐Hsin Tai, Zheng Chen, Astrid Maluta, Mohammed Hakil, Bianca Susini, Hannah Florance, Peter‐Leon Hagedoorn, John Mark Sutton, Maria‐Andrea Mroginski, Simone Ciofi‐Baffoni, Kourosh H. Ebrahimi

**Affiliations:** ^1^ Institute of Pharmaceutical Science King's College London London UK; ^2^ Department of Pharmacy Da Nang University of Medical Technology and Pharmacy Da Nang Vietnam; ^3^ Magnetic Resonance Center and Department of Chemistry University of Florence Sesto Fiorentino Tuscany Italy; ^4^ Department of Chemistry Technical University of Berlin Berlin Germany; ^5^ Institution: Agilent Technologies Cheadle UK; ^6^ Department of Biotechnology Delft University of Technology Delft the Netherlands; ^7^ UK Health Security Agency Salisbury UK

**Keywords:** CYB5R3, electron transfer, nucleotide analogues, radical‐SAM enzymes, RSAD2 (viperin)

## Abstract

Engineering immune‐silent, catalytically self‐sufficient antiviral restriction factor enzymes (iCAREs) provides a proof‐of‐concept for developing autonomous enzymes for future antiviral applications. To rationally design these enzymes, we sought to identify and engineer components of the intrinsic immune system. Here, we identified the endoplasmic reticulum (ER)‐anchored cytochrome b5 reductase 3 (CYB5R3) as a putative electron donor to the ER‐anchored and interferon‐stimulated antiviral radical S‐adenosylmethionine (SAM)‐dependent nucleotide dehydratase (SAND), or RSAD2 (viperin) in humans. We demonstrate that their functional partnership depends on co‐localisation. We obtained insights into the structure of the RSAD2:CYB5R3 complex and, using mutagenesis, identified the structural elements required for electron transfer. Based on this discovery, we engineered iCAREs that autonomously generate an antiviral nucleotide analogue to chain‐terminate viral RNA polymerase activity. Because iCAREs are self‐sufficient and do not require recruitment of endogenous redox partners, they are predicted to avoid interference with numerous biological processes associated with single‐domain redox partners such as CYB5R3, including electron delivery to the oncogenic enzyme stearoyl‐CoA desaturase (SCD1). This work solves a long‐standing question of why ER localisation is critical for RSAD2 activity, resolves a key mechanistic question in radical‐SAM enzymology, and establishes a general strategy to engineer self‐sufficient radical‐SAM enzymes for biotechnological applications.

## Graphical abstract

CYB5R3 is identified as a putative electron‐transfer partner of viperin (RSAD2), explaining why ER localisation is required for antiviral activity. Its utilisation enabled the engineering of immune‐silent, catalytically self‐sufficient antiviral restriction factor enzymes (iCAREs) that produce an antiviral nucleoside triphosphate analogue (ANTA), thereby enabling the development of novel antiviral strategies.

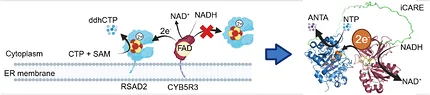

## 1 Introduction

**FIGURE 1:**
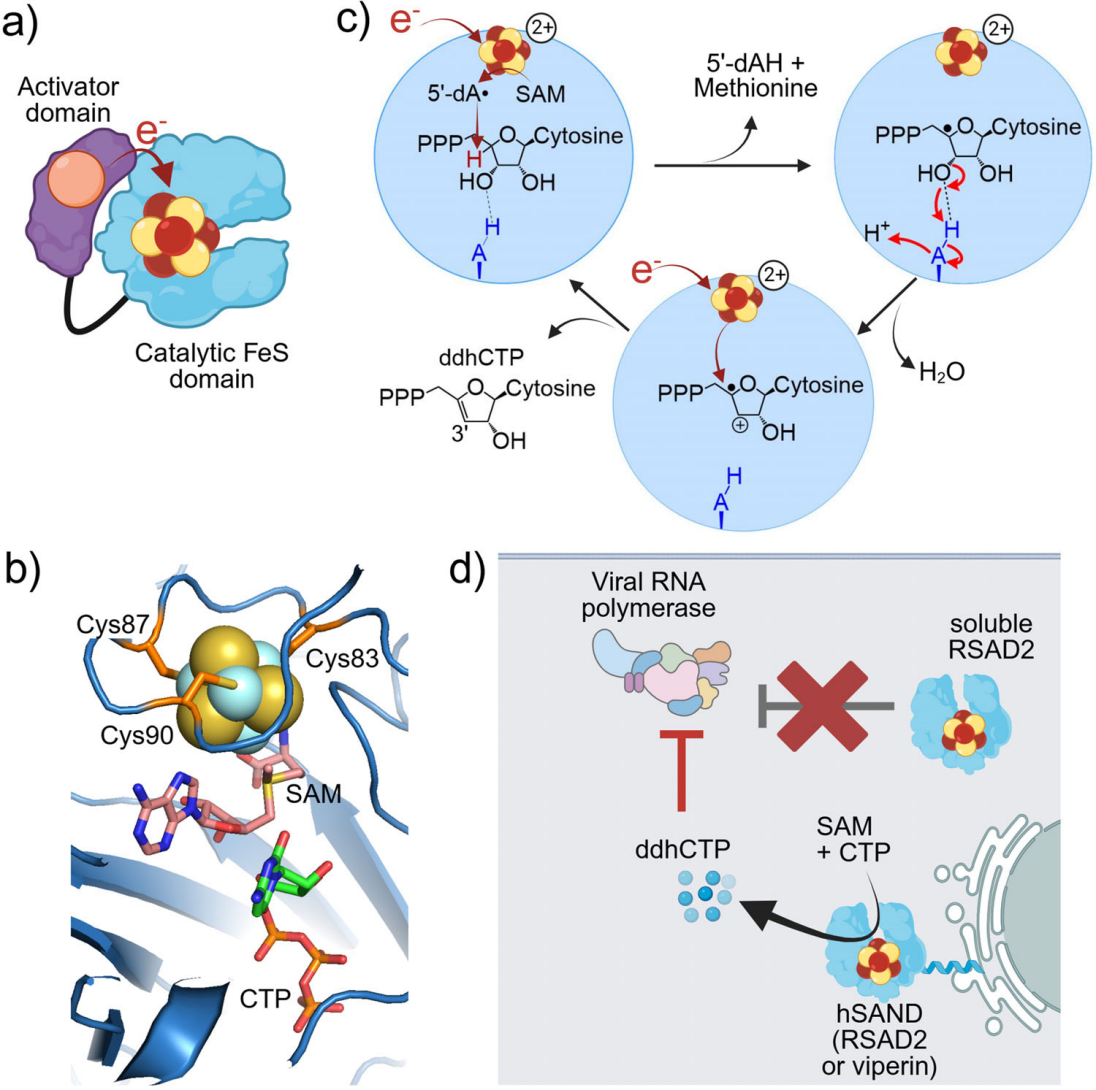
The human radical‐SAM enzyme RSAD2 requires two electrons to catalyse the transformation of CTP to ddhCTP. (a) A schematic depiction of a self‐sufficient radical‐SAM enzyme composed of two domains: a catalytic iron‐sulphur (FeS) cluster domain and an activator domain. (b) RSAD2 is a radical‐SAM enzyme and contains a [4Fe‐4S] cluster coordinated to three cysteine residues. (c) The proposed mechanism of catalytic transformation of CTP (the preferred substrate) to ddhCTP by RSAD2. The first electron is used by the cluster to reductively cleave the SAM, generating a 5′‐deoxyadenosyl radical (5′‐dA•), which abstracts the H4′ atom from the CTP substrate. This hydrogen atom transfer reaction generates 5′‐deoxyadenosine (5′‐dAH), methionine, and a substrate radical intermediate. The latter undergoes rearrangement reactions to lose its 3′‐hydroxyl group and receives a second electron to form ddhCTP. Because the ddhCTP lacks the 3′‐hydroxyl group, it can chain‐terminate the activity of viral RNA polymerases. (d) RSAD2 or human SAND (hSAND) (viperin) is localised to the ER membrane by its N‐terminal amphipathic domain. It catalyses the transformation of CTP to ddhCTP as the preferred substrate. This ANTA chain terminates the activity of viral RNA polymerases. A soluble form of RSAD2 lacking the N‐terminus amphipathic domain cannot localise to the ER and restrict viral RNA polymerase activity through chain‐termination activity of ddhCTP.

Despite the significance of self‐sufficient enzymes for biotechnological applications [1, 2, 3, 4, 5, 6, 7, 8] and the growing interest in the biocatalytic use of the radical S‐adenosylmethionine (SAM) superfamily of enzymes [9, 10, 11], for example, synthesis of antimicrobials or antivirals, a self‐sufficient radical‐SAM enzyme has yet to be engineered. This enzyme would have at least two domains (Figure 1a): the catalytic radical‐SAM domain, which carries out a specific biochemical transformation, and an activator domain, which generates the electrons required for catalysis. In this respect, engineering radical‐SAM enzymes to create immune‐silent, catalytically self‐sufficient antiviral restriction factor enzymes (iCAREs) would offer a radically new preventive antiviral approach: mRNA delivery for ectopic expression of the enzyme in cells and the enzymatic production of broad‐spectrum antivirals within the body. Unlike natural single‐domain redox enzymes, which rely on cellular partners participating in many native pathways, iCAREs would operate independently, thereby minimising the risk of perturbing endogenous biological processes.

A radical‐SAM enzyme suitable as the catalytic domain of iCAREs is an interferon (IFN)‐stimulated protein localised at the cytosolic face of the endoplasmic reticulum (ER) and lipid droplets by its N‐terminal amphipathic domain (Figure 1b) [12, 13, 14]. Consequently, it was originally named viperin (virus‐inhibitory protein, endoplasmic reticulum‐associated, interferon‐inducible) [15, 16, 17, 18]. The enzyme is also known as RSAD2 (Radical S‐Adenosyl Methionine Domain Containing 2) or SAM‐dependent nucleotide dehydratase (SAND) as recognised by UniProt [12]. It is identified primarily as an antiviral effector [19]. It restricts viral replication through different mechanisms [20, 21, 22, 23, 24, 25]. Human RSAD2 and its homologues from different species catalyse the transformation of a nucleoside triphosphate (NTP) to its antiviral nucleoside triphosphate analogue (ANTA) ddhNTP (3′‐deoxy‐3′,4′‐didehydro‐NTP) [26, 27, 28]. The enzyme turnover number is approximately 2.2–4 h^−1^ [29, 30], similar to that of many other radical‐SAM enzymes [31]. This chemical transformation requires two electrons (Figure 1c), the source of which has remained elusive [24, 28]. Additionally, the N‐terminal domain is known to be important for antiviral activity against many viruses [16, 32, 33] and inhibition of T7 RNA polymerase activity. The latter is because a soluble form of the protein lacking the N‐terminus (Figure 1d) [34] does not possess chain‐termination activity [35, 36]. However, the mechanism by which the N‐terminal amphipathic domain may affect catalytic function and antiviral activity remains unclear.

Here, we sought to identify an electron‐transfer partner (ETP) for RSAD2 and to engineer their fusion as an iCARE candidate. To this end, we employed a multidisciplinary approach integrating bioinformatics, biochemical and molecular biology, metabolomics, diamagnetic and paramagnetic NMR spectroscopy, AI‐based structural predictions, molecular dynamics (MD) simulations, and mutagenesis studies. We identified ER‐anchored cytochrome b5 reductase 3 (CYB5R3), a key cellular redox regulator [37], as a putative ETP of RSAD2. We demonstrate that co‐localisation is key to their partnership and shed light on the mechanism of electron transfer from CYB5R3 to RSAD2. By fusing RSAD2 and CYB5R3, we show that we can bypass the need for co‐localisation and create iCARE candidates with improved activity. Our combined results extend our understanding of radical‐SAM catalysis, address a key mechanistic question regarding the antiviral activity of RSAD2, and introduce a step‐change in the biotechnological application of radical‐SAM enzymes.

## 2 Results and Discussion

### 2.1 Prediction of CYB5R3 as a Putative ETP Co‐Localised With RSAD2

**FIGURE 2:**
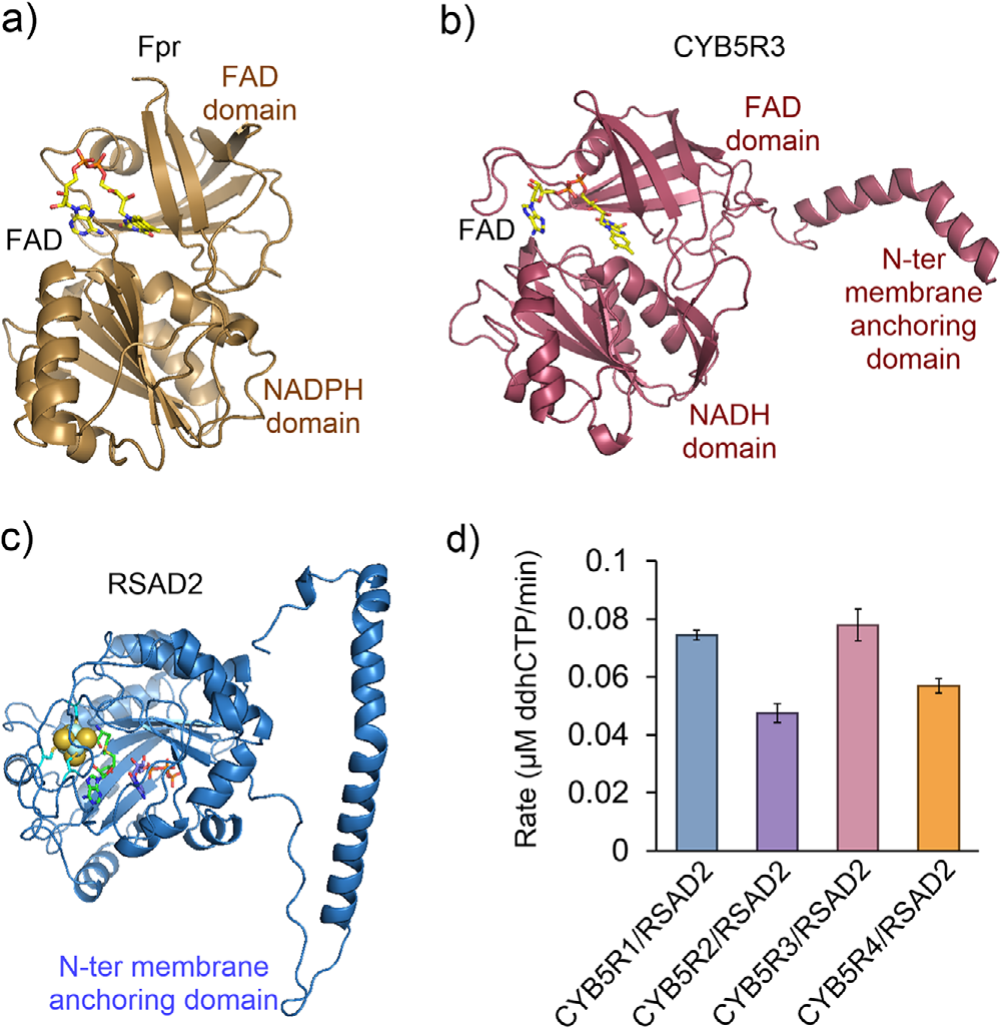
In vitro, soluble forms of CYB5R members donate electrons to soluble RSAD2 (hSAND or viperin). (a) The x‐ray crystal structure of *E. coli* flavodoxin reductase (Fpr) (PDB Code: 1FDR). (b) An AlphaFold‐predicted and energy‐optimised structure of CYB5R3 in complex with FAD cofactor and (c) RSAD2 in complex with [4Fe‐4S] cluster, SAM, and CTP. Both CYB5R3 and RSAD2 have an N‐terminal amphipathic membrane‐anchoring domain. (d) The enzymatic rate (µM ddhCTP produced per minute) of RSAD2 (55 µM) in vitro in the presence of each CYB5R member (11 µM) was determined using an endpoint measurement assay (methods). Each reaction was incubated overnight (approximately 17 h). The CTP, SAM, and NADH concentrations were 3.6–5.5, 14–22, and 5.6–7.1 mM, respectively. Data are the average of two measurements (*n* = 2) ± errors. The experiments with different CYB5R members were performed in parallel and compared with CYB5R3 as a reference to ensure consistent conditions. A truncated soluble form of RSAD2, CYB5R1, and R3, lacking the N‐terminal amphipathic domain, was used.

In bacteria, a flavodoxin/flavodoxin‐reductase (Fld/Fpr) system or ferredoxin/ferredoxin reductase (Fdx/FdxR) is believed to generate electrons from NADH/NADPH and provide them to some radical‐SAM enzymes in a species‐specific manner [38, 39]. These systems are absent in the cytoplasm of human cells. We used bioinformatics to identify homologues of these systems as potential ETPs of human cytosolic radical‐SAM enzymes. We specifically examined predicted proteins homologous to the bacterial reductases, for example, flavodoxin reductase (Fpr) (Figure 2a). It is shown that removal of the N‐terminus amphipathic domain of RSAD2 abolishes its localisation to the ER membrane and eliminates its chain‐termination activity measured by inhibition of T7 RNA polymerase in HEK293 cells [34]. Therefore, we hypothesised that the N‐terminus is essential for the co‐localisation of RSAD2 and its ETP in the ER membrane, electron transfer, and ddhCTP formation. Accordingly, we searched for reductases known to be anchored to the cytosolic face of the ER membrane, similar to human RSAD2. Using the ProteinPrompt server [40], we identified CYB5R3 as a potential ETP of RSAD2. This reductase is known to be localised to the cytosolic face of the ER membrane [37], the same as RSAD2 [11]. The AlphaFold‐predicted structure of CYB5R3 exhibits a structural fold containing an FAD and NADH/NADPH binding domain (Figure 2b), similar to *E. coli* flavodoxin reductase (Figure 2a), and an N‐terminal membrane‐anchoring domain (Figure 2b), the same as RSAD2 (Figure 2c). CYB5R3 is one of four closely related cytochrome‐b5 reductases (CYB5R), annotated as CYB5R1‐4 [37]. These enzymes are conserved from fungi to humans. While CYB5R3 is shown to localise to the ER membrane [37], CYB5R2‐4 are predominantly localised to other cellular compartments. CYB5R1 is present in the outer mitochondrial membrane [41], and CYB5R2 and R4 lack an N‐terminal amphipathic domain (Figures S1,S2 and Table S1) and are predicted to localise to cellular compartments such as the Golgi. They are predicted to interact with eight human radical‐SAM enzymes (Table S2).

### 2.2 In Vitro Biochemical Characterisation of CYB5R Members

To test electron transfer from a CYB5R member to RSAD2, we first overexpressed and purified the soluble forms of all four CYB5R members by removing the N‐terminal amphipathic domain from CYB5R1 and R2 (Supplementary Methods). To confirm proper folding and activity, we monitored the presence of cofactors FAD (CYB5R1‐R3) and haem (CYB5R4), as well as enzymatic activity (Figure S3). The purified CYB5R1‐3 contained FAD (Figure S3), and CYB5R4 contained FAD capable of oxidising NADH to reduce the haem in its Cyt‐b5 domain (Figure S3). All enzymes catalysed the oxidation of NADH with the apparent *K_m_* values of 0.9 ± 0.3, 1.1 ± 0.3, 0.9 ± 0.2, and 6.2 ± 1.8 µM for CYB5R1, R2, R3, and R4, respectively (Figure S3). Therefore, all purified enzymes were correctly folded and were active. In vitro, all CYB5R members could donate electrons to soluble RSAD2 (lacking the N‐terminal amphipathic domain) and catalyse the synthesis of ddhCTP, as measured by liquid chromatography‐mass spectrometry (LC‐MS) (Figure 2d and Figures S4,S5). Consistently, a pull‐down assay showed that all CYB5R members weakly interacted with RSAD2 (Figure S6). The in vitro ability of all CYB5R members to interact with RSAD2 (Figure S6) and provide electrons for catalysis (Figure 2d) is somewhat expected, as the microbial reductase system can non‐specifically provide electrons to human RSAD2 (viperin) in vitro [27] and inside *E. coli* cells [36]. Therefore, the specificity of CYB5R3 to RSAD2 is not driven by a strong interaction between the two proteins. This conclusion aligns with transient protein‐protein interactions observed in other electron‐transfer systems and with the importance of redox centre orientation in the cellular environment for efficient encounter and rapid electron transfer [42, 43, 44, 45, 46].

### 2.3 Co‐Localisation Defines CYB5R3 Specificity to RSAD2

**FIGURE 3:**
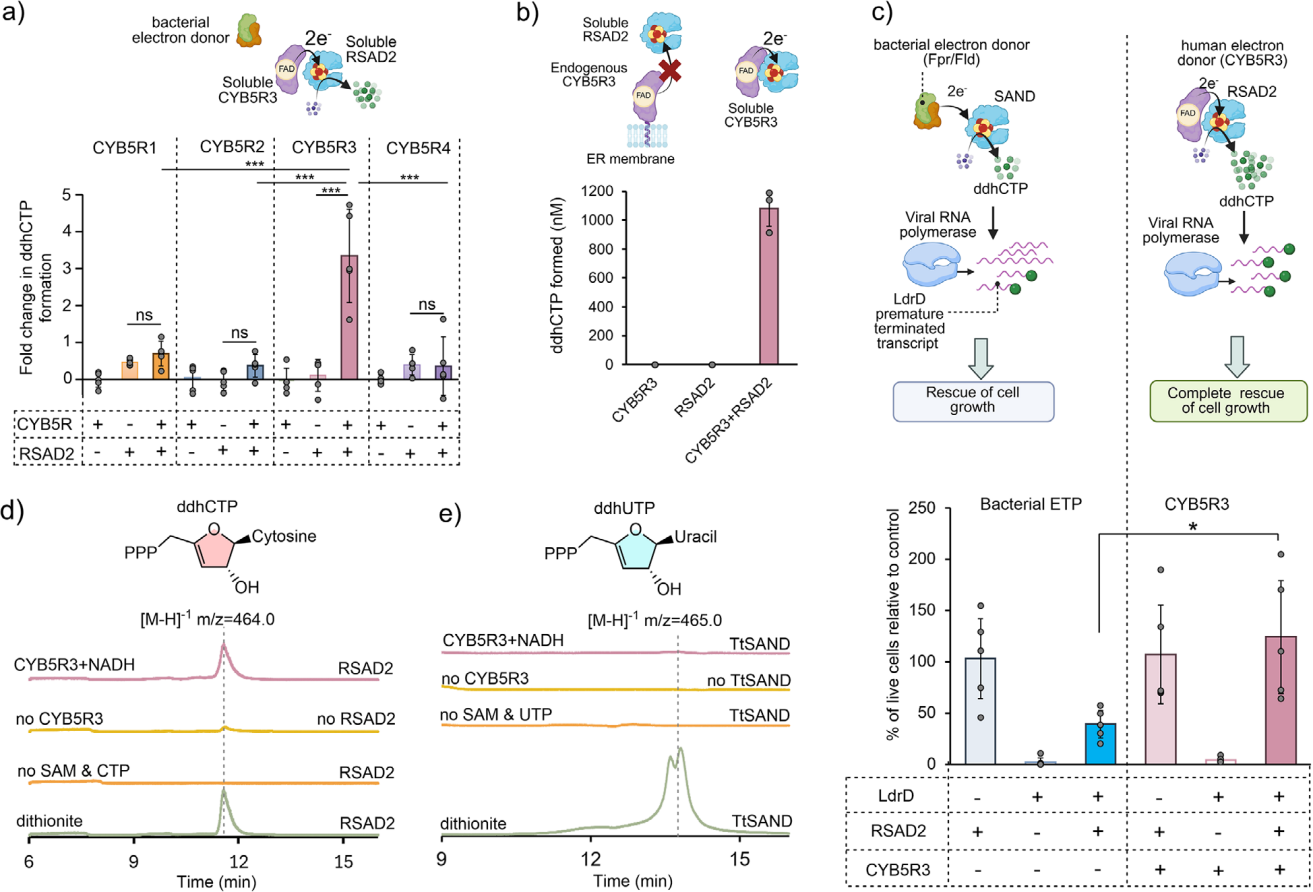
Co‐localisation is essential for the functional electron transfer from CYB5R3 to RSAD2 (hSAND or viperin). (a) Targeted metabolomic studies of ddhCTP formation in *E. coli* cells after co‐transformation with plasmids expressing RSAD2/CYB5R1, RSAD2/CYB5R2, RSAD2/CYB5R3, or RSAD2/CYB5R4. The fold‐change increase in ddhCTP formation (Equation 1, Supplementary methods) due to the expression of a CYB5R member is plotted relative to cells expressing a CYB5R or RSAD2 alone. Data are an average of five (*n* = 5) biologically independent samples ± standard deviation and are corrected for the expression level of each CYB5R member. One‐way ANOVA analysis was used to calculate *p* values. (b) Targeted metabolomic studies of ddhCTP formation in human HEK293 cells transfected with plasmids expressing soluble RSAD2, CYB5R3, or both RSAD2 and CYB5R3. Data are an average of three (*n* = 3) independent samples ± standard deviation. HEK293 cells exhibit high‐level endogenous full‐length CYB5R3 (ER‐anchored) expression. (c) VITAS assay to measure chain‐termination activity inside cells. The expression of CYB5R3 together with RSAD2 and LdrD fully rescues growth relative to live control (cells expressing RSAD2 or cells co‐expressing RSAD2 and CYB5R3). Data are an average of five (*n* = 5) biologically independent samples ± standard deviation. One‐way ANOVA analysis was used to calculate *p* values. (d) CYB5R3 (28 µM) activates the catalytic activity of RSAD2 (28 µM) but not (e) *Tt*SAND (28 µM). (d) Extracted ion chromatogram of ddhCTP, [M − H]^−1^ (m/z) = 464.0 ± 0.1 and (e) ddhUTP, [M − H]^−1^ (m/z) = 465.0 ± 0.1. The concentrations of dithionite, CTP or UTP, SAM, and NADH were 2.8 mM, 2.8 mM, 11.2 mM, and 5.6 mM, respectively. In all experiments (a–d), soluble forms of RSAD2, CYB5R1, and CYB5R3, lacking the N‐terminal amphipathic domain, were used. *p value ≤ 0.05; **p value ≤ 0.01; ***p value ≤ 0.001. ns: not significant.

Previously, cellular assays were used to determine the specificity of an ETP to a radical‐SAM enzyme [38]. Accordingly, we performed in‐cell competition assays to test which CYB5R member can compete with *E. coli* reductase to improve ddhCTP synthesis. We used the soluble forms of CYB5R1, R3, and RSAD2, whose N‐terminal amphipathic domain was truncated, and full‐length R2 and R4, which don't have an N‐terminal amphipathic domain (Supplementary Methods). Using Western blot analysis, we first determined the expression levels of CYB5R1, R2, R3, and R4 to demonstrate that our expression system is robust and to correct metabolomics data for variations in protein expression levels (Figure S7). Subsequently, we conducted targeted metabolomic analyses and measured the formation of ddhCTP in *E. coli* cells co‐expressing soluble RSAD2 and soluble CYB5R. We compared the results with cells expressing soluble RSAD2 alone or, as a negative control, soluble CYB5R3 (Supplementary Methods) (Figure 3a and Table S3). The results revealed that when CYB5R3 and RSAD2 were co‐expressed, there was an 8‐ to 10‐fold increase in ddhCTP formation compared to the RSAD2‐alone control, in which the *E. coli* reductase system provides electrons for ddhCTP formation (Figure 3a). However, co‐expression of CYB5R1, R2, or R4 with RSAD2 did not result in any significant ddhCTP formation compared with the RSAD2‐alone control (Figure 3a).

Overexpression of the full‐length RSAD2 (ER‐anchored) in HEK293 drives the synthesis of ddhCTP [27]. In contrast, deletion of the N‐terminal amphipathic domain of RSAD2 abolishes both ER localisation and ddhCTP production, as evidenced by the loss of chain‐termination activity in the T7 polymerase assay conducted in HEK293 cells [34]. To further assess whether co‐localisation is essential for electron transfer, we utilised HEK293T cells. These cells exhibit high‐level endogenous expression of full‐length CYB5R3 (localised to the ER membrane) but lack RSAD2 expression (Figure S8). We expressed a soluble RSAD2 (lacking the N‐terminal amphipathic domain) and analysed ddhCTP production (Figure S8). Consistent with previous findings [34], we did not observe ddhCTP formation similar to the control cells expressing CYB5R3 alone (Figure 3b). However, when we bypassed the need for ER co‐localisation by co‐expressing soluble forms of RSAD2 and CYB5R3, a significant amount of ddhCTP was detected (Figure 3b). Therefore, our data reveal why the N‐terminus amphipathic of RSAD2 is essential for ddhCTP formation [27, 34]. It couples co‐localisation with electron transfer from the putative partner CYB5R3. The soluble form of RSAD2 cannot accept electrons from other cytosolic soluble reductases, such as CYB5R4, or form the correct orientation with its putative ER‐anchored ETP CYB5R3 to synthesise ddhCTP.

### 2.4 CYB5R3 Improves the Chain‐Termination Activity of Human RSAD2

Next, we investigated whether human CYB5R3 expression would improve the chain‐termination activity of human RSAD2. We applied our live‐dead cell assay (Figure 3c), named VITAS (Viral polymerase‐Inhibition Toxin Associated Selection) [36]. VITAS measures how efficiently the ddhCTP formation chain terminates the activity of viral T7 RNA polymerase in generating the toxin LdrD protein transcript (mRNA), thereby protecting *E. coli* cells and increasing cell growth (Figure 3c). The concomitant expression of RSAD2 and LdrD partially rescued (approximately 46%) the growth of *E. coli* cells as compared to live (RSAD2 alone) and dead (LdrD alone) controls (Figure 3c). This observation is consistent with our published data [36], suggesting inefficient electron transfer from bacterial reductase. When CYB5R3 was expressed together with RSAD2, the growth of *E. coli* cells increased by 50% and was fully recovered (Figure 3c) as compared to the live control, in which RSAD2 and CYB5R3 were co‐expressed in the absence of LdrD. This observation is consistent with electron transfer from human CYB5R3 to human RSAD2, which increases ddhCTP formation, as indicated by metabolomics data (Figure 3a), thereby improving chain‐termination activity. The specificity of CYB5R3 to RSAD2 was further confirmed in vitro. While human CYB5R3 was able to support ddhCTP formation by human RSAD2 (Figure 3d and Figure S4), it was unable to support ddhUTP formation by its fungal homologue from *Thielavia terrestris* (*Tt*SAND) in vitro (Figure 3e and Figure S9) or inside cells (Figure S10).

### 2.5 Monitoring the Oxidation State of FAD

**FIGURE 4:**
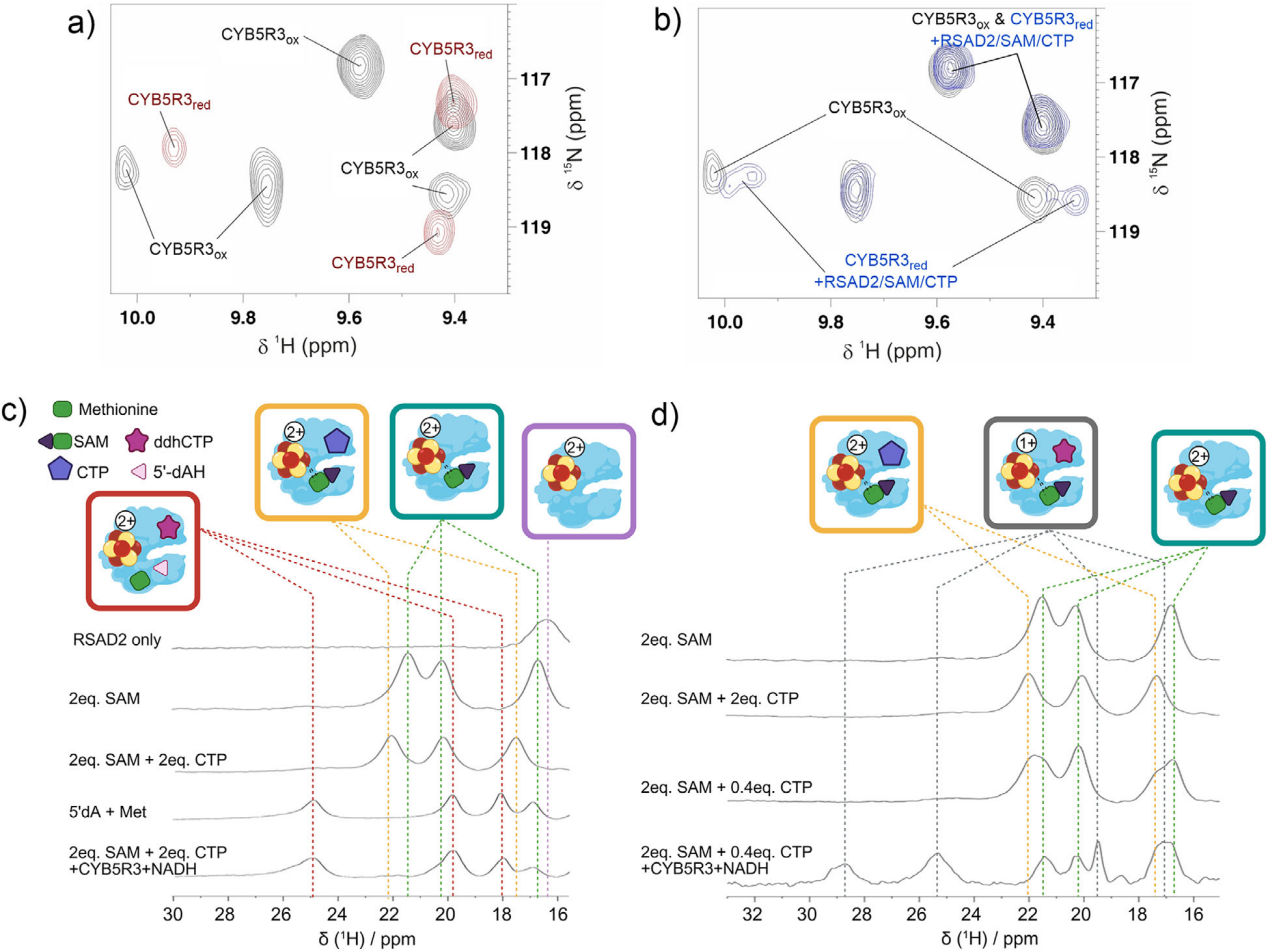
Monitoring the oxidation state of the FAD cofactor in CYB5R3 and the [4Fe‐4S] cluster of RSAD2. (a) ^1^H‐^15^N HSQC NMR spectra of ^15^N‐labelled oxidized CYB5R3 (CYB5R3_OX_) and NADH‐reduced CYB5R3 (CYB5R3_red_), and (b) that of oxidized CYB5R3 (CYB5R3_OX_) followed by the addition of unlabelled [4Fe‐4S]^2+^ RSAD2, CTP, and SAM. The same amount of NADH, SAM, and CTP was added. (c) Paramagnetic 1D ^1^H NMR spectra of [4Fe‐4S]^2+^ RSAD2 (purple box), and [4Fe‐4S]^2+^ RSAD2 in the presence of SAM alone (green box), SAM and CTP (yellow box), Met and 5’‐dAH, or after the addition of SAM, CTP, CYB5R3, and NADH to [4Fe‐4S]^2+^ RSAD2 leading to product formation (red box). Two equivalents (eq.) of SAM and CTP were added. (d) The paramagnetic 1D ^1^H NMR spectrum of the mixture containing 2 eq. SAM and 0.4 eq. CTP contains two species (yellow and green boxes) as a result of the comparison with the paramagnetic 1D ^1^H NMR spectra of [4Fe‐4S]^2+^ RSAD2 in the presence of 2 eq. SAM alone (green box) and of 2 eq. SAM and 2 eq. CTP (yellow box). The new signals in the paramagnetic 1D ^1^H NMR spectrum of the same mixture after adding 0.5 eq. CYB5R3 and an excess of NADH identify the formation of a new RSAD2 species containing a reduced [4Fe‐4S]^1+^ cluster (grey box). These signals originate from SAM‐ and CTP‐bound [4Fe‐4S]^2+^ RSAD2, whose NMR signals disappear upon addition of CYB5R3 and NADH, forming SAM‐ and ddhCTP‐bound, reduced [4Fe‐4S]^1+^ RSAD2. The NMR signals from the SAM‐[4Fe‐4S]^2+^ RSAD2 cluster (green box), which cannot receive electrons in the absence of CTP, are still present in the final mixture.

To provide unambiguous evidence of electron transfer in our system, we investigated changes in the oxidation state of CYB5R3 FAD. We employed ^1^H‐^15^N‐ HSQC NMR spectroscopy and monitored the FAD oxidation state by following protein NMR signals. We recorded the ^1^H‐^15^N HSQC spectra of ^15^N‐labelled CYB5R3 (Supplementary Methods) before and after the addition of NADH (Figure S11). We monitored the chemical shifts of the backbone NH signals to identify the spectral fingerprints of FAD‐oxidized and ‐reduced CYB5R3 species (Figure 4a). Next, we added [4Fe‐4S]^2+^ RSAD2 and an equimolar amount of CTP, SAM, and NADH. We observed that in the ^1^H‐^15^N HSQC spectrum of the final mixture, the majority of the backbone NH signals of the FAD‐reduced CYB5R3 returned to those of the FAD‐oxidized CYB5R3 (Figure 4b and Figure S11). This observation indicates FAD re‐oxidation, confirming the electron transfer from reduced FAD to the oxidised [4Fe‐4S]^2+^ cluster of RSAD2, enabling the catalytic conversion of CTP to ddhCTP, as measured by LC‐MS in vitro and *inside cells* (Figures 2, 3). However, some backbone NH signals were not fully recovered to the same chemical shifts observed for FAD‐oxidized CYB5R3. They exhibited chemical shifts significantly different from those of FAD‐reduced CYB5R3 (Figure 4b). This observation is consistent with a transient interaction between RSAD2 and CYB5R3.

### 2.6 Electron Transfer Requires CTP and SAM Binding

Using UV‐visible spectroscopy, we initially observed that upon complete consumption of SAM and in the presence of excess NADH, the FAD cofactor in CYB5R3 was reduced, with no peaks observed for the oxidised FAD cofactor (Figure S12). This observation suggests that electron transfer requires at least SAM binding to the protein. To clearly define the prerequisites for electron transfer from CYB5R3 FADH_2_ to the RSAD2 [4Fe‐4S]^2+^ cluster, we used paramagnetic 1D ^1^H NMR spectroscopy to follow changes in the RSAD2 [4Fe‐4S] cluster environment due to the addition of CTP, SAM, and CYB5R3/NADH. This approach is a powerful tool for monitoring the oxidation state of the cluster [47, 48, 49]. In this approach, NMR signals from the oxidised [4Fe‐4S]^2+^ cluster show anti‐Curie temperature dependence, while those of the reduced [4Fe‐4S]^1+^ cluster show anti‐Curie and Curie‐type temperature dependence [47, 48, 49]. The binding of SAM to the oxidised RSAD2 [4Fe‐4S]^2+^ cluster changed hyperfine‐shifted proton signals of the cluster ligands (Figure 4c). The position of the proton hyperfine‐shifted signals and their anti‐Curie temperature dependence indicate that the signals originate from SAM‐bound oxidised [4Fe‐4S]^2+^ RSAD2 (Figure S13). In the presence of either SAM or CTP, the addition of CYB5R3 in the absence or presence of NADH did not affect the NMR spectra (Figure S14). This observation suggests electron transfer from CYB5R3 FADH_2_ to the oxidised [4Fe‐4S]^2+^ cluster in RSAD2 requires the binding of both SAM and CTP. Consequently, when both SAM and CTP were present, and CYB5R3 together with NADH was added, we observed a dramatic change in the hyperfine‐shifted proton signals of the cysteine ligand of the cluster (Figure 4c and Figure S15). The hyperfine‐shifted proton signals showed anti‐Curie temperature dependence (Figure S16), indicating that they originate from a species with an oxidised [4Fe‐4S]^2+^ cluster after catalysis. This species likely corresponds to the [4Fe‐4S]^2+^ of RSAD2 bound to reaction products, that is, methionine (Met), 5′‐dAH and/or ddhCTP. The 1D ^1^H NMR spectrum of [4Fe‐4S]^2+^ RSAD2 obtained in the presence of the reaction products Met and 5′‐dAH fully reproduced the key four hyperfine shifted signals in the 27–15 ppm region assigned to the reaction products, observed in the reaction mixture containing [4Fe‐4S]^2+^ RSAD2, CYB5R3, CTP, SAM and NADH (Figure 4c and Figure S15). In conclusion, NMR data monitoring the oxidation states of FAD and the [4Fe‐4S] cluster demonstrate electron transfer from reduced FADH_2_ in CYB5R3 to the RSAD2‐oxidized [4Fe‐4S]^2+^ cluster in a SAM‐ and CTP‐dependent manner.

### 2.7 NMR Detection of the Reduced [4Fe‐4S]^1+^ Cluster

When equal amounts of CTP and SAM (each at two equivalents of RSAD2 concentration) were present, they were entirely consumed, and we did not detect a reduced [4Fe‐4S]^1+^ cluster. Therefore, we tested whether we could measure the formation of the reduced [4Fe‐4S]^1+^ cluster when less CTP was added relative to SAM. We performed analysis in the presence of 0.4 and 2 equivalents (relative to RSAD2) of CTP and SAM, respectively. Like before, in the absence of NADH, only the hyperfine‐shifted protons of [4Fe‐4S] cysteine residues bound to SAM and CTP were observed (Figure 4d). Upon addition of excess NADH with CYB5R3, the signals assigned to the CTP‐ and SAM‐bound cluster species disappeared, but those of the SAM‐bound [4Fe‐4S] cluster species were present (Figure 4d). This observation indicates that only the CTP‐ and SAM‐bound cluster species undergo consumption in the reaction. Under this condition, we observed new NMR signals in the 30‐10 ppm region (Figure 4d), which do not match those of SAM‐bound [4Fe‐4S]^2+^ cluster species. These signals are also distinct from those assigned to reaction products and obtained in the mixture containing equal amounts of CTP and SAM (Figure 4c). Additionally, we observed new signals at 125–35 ppm (Figure S17). The 30–10 ppm and 124–35 ppm signals showed anti‐Curie and Curie‐type temperature dependence, respectively, consistent with the formation of a reduced [4Fe‐4S]^1+^ cluster (Figure S17). This species is likely formed by the displacement of 5′‐dAH and methionine by extra SAM added to a subset of enzymes bound to the ddhCTP product, which, like CTP‐binding, potentially allows electron transfer from FADH_2_ to the SAM‐bound [4Fe‐4S]^2+^ cluster.

### 2.8 Resolving the CYB5R3 Surface Recognised by RSAD2

**FIGURE 5:**
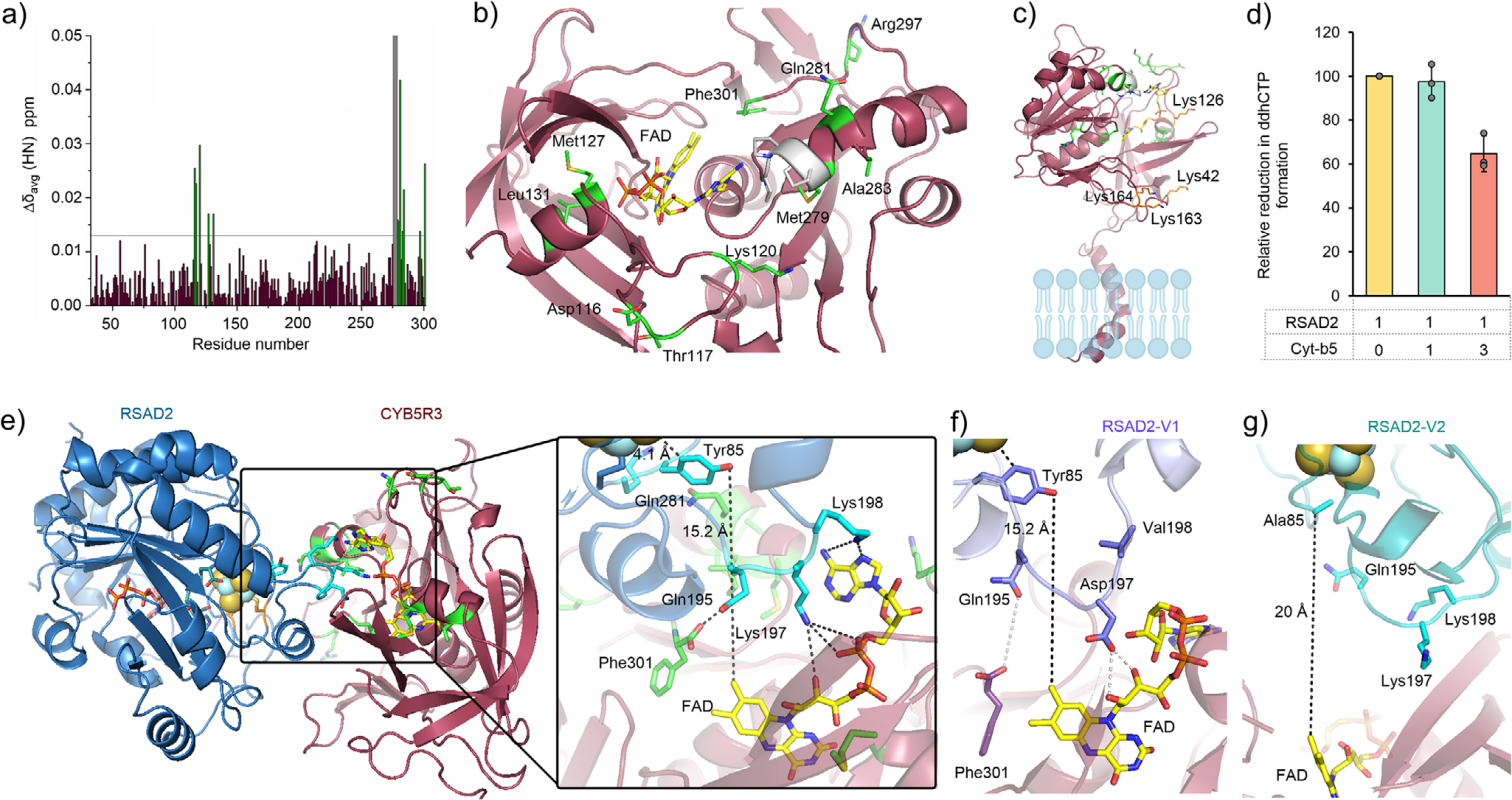
RSAD2 and Cyt‐b5 share the same binding site on CYB5R3. (a) Backbone‐weighted average chemical shift differences (Δ*δ*_avg_(HN)) between ^15^N‐labelled CYB5R3 before and after the addition of 1 eq. of unlabelled [4Fe‐4S]^2+^ RSAD2 and an equimolar amount of CTP, SAM, and NADH. A chemical shift threshold value, indicated as a line, defines the significant chemical shift differences, indicated by green bars. The grey bars indicate prolines 276–278. These proline residues are part of a short α‐helix that interacts with RSAD2. (b) The chemical shift changes displayed with grey (three prolines) and green bars in panel (a) are mapped on the crystal structure of CYB5R3 (PDB ID 1UMK) and the modelled structure of CYB5R3. (c) The figure shows that the NMR‐detected surface of CYB5R3 interacting with RSAD2 (green residues) is shared with the surface accessible to ER‐anchored Cyt‐b5 (orange residues). (d) Cyt‐b5 competes with RSAD2 to receive electrons from CYB5R3. The relative reduction (as compared to control in the absence of Cyt‐b5) in ddhCTP produced by the CYB5R3/RSAD2 couple when the Cyt‐b5:RSAD2 ratio was 1:1 and when it was 3:1. The concentration of RSAD2 and CYB5R3 was 30 µM, and those of NADH, CTP, and SAM were 6, 3, and 12 mM. Data are the average of two (*n* = 2) measurements ± errors. (e) The prediction of RSAD2 in complex with CYB5R3. The inset structure shows that the exposed Q_195_GKK surface loop interacts with the CYB5R3 FAD cofactor and that RSAD2 Tyr85 is along the electron transfer path from FAD to the [4Fe‐4S] cluster. (f) The interactions of the surface loop with FAD in the CYB5R3/RSAD2‐V1 complex are partially disrupted, and (g) the interactions with FAD in the CYB5R3/RSAD2‐V2 complex are fully abolished.

To identify the surface of CYB5R3 interacting with RSAD2, we produced ^13^C,^15^N‐labelled CYB5R3 and performed the backbone NMR assignment of the oxidised FAD form of CYB5R3. We successfully assigned 96% of the backbone signals detected in the ^1^H‐^15^N HSQC map (Figure S18). With this information in hand, we were able to map the chemical shift changes of CYB5R3 in the mixture containing FAD‐oxidized CYB5R3, [4Fe‐4S]^2+^ RSAD2, and equimolar amounts of CTP, SAM, and NADH, the same mixture discussed above (Figure 4b and Figure S11). The backbone NMR chemical shifts (δ^15^N, δ^13^C′, δ^13^C^α^, δ^13^C^β^ and δ^1^H^N^) of FAD‐oxidized CYB5R3 were first used to perform secondary structure analysis by TALOS‐N [50], showing that the elements of secondary structure well reproduce those present in the crystal structure of CYB5R3 (Figure S18). Weighted‐average backbone chemical shift differences (Δ*δ*_avg_(HN)) between FAD‐oxidized CYB5R3 and the mixture were then calculated (Figure 5a) and mapped on the crystal structure of CYB5R3 (Figure 5b). The chemical shift changes are small, consistent with the transient interaction between the two proteins, and chemical shift perturbations above the threshold (indicated in green in Figure 5a,b) clearly identify two regions: a region on the surface composed of a short α‐helix (residues 276–281), the C‐terminal (Arg297 and Phe301), and a region surrounding FAD composed of a loop (including Lys120) and a second short α‐helix in which two buried residues, namely Met127 and Leu131, are in contact with FAD (Figure 5b).

### 2.9 The NMR‐Detected Surface of CYB5R3 Is Shared Between RSAD2 and Cyt‐b5

Considering the orientation of the N‐terminus amphipathic domain of CYB5R3, the NMR‐resolved surface of CYB5R3 interacting with RSAD2 is available for binding to partner proteins (Figure 5c). This surface is shared with that shown to interact with the membrane‐anchored cytochrome b5 (Cyt‐b5) (Figure 5c and Figure S19) [51], the known electron acceptor of CYB5R3. It is also consistent with a similar surface observed for the interaction of the Cyt‐b5 domain of soluble CYB5R4, as predicted by our modelled structure (Figure S19) and observed in the X‐ray crystal structure [52]. Hence, we tested if RSAD2 and Cyt‐b5 compete for the same surface (Figure 5d). We added RSAD2 to CYB5R3 in the absence of Cyt‐b5 (control) or the presence of one or three equivalents (relative to RSAD2) of Cyt‐b5 (soluble form) (Figure 5d). When the Cyt‐b5/RSAD2 ratio was one in the presence of excess NADH, we did not observe a significant impact on ddhCTP formation (Figure 5d). However, when the concentration of Cyt‐b5 was 3‐fold more than that of RSAD2, a 30–40% decrease in ddhCTP formation was observed (Figure 5d). These results are consistent with NMR data showing that RSAD2 interacts with CYB5R3 (Figure 5b). More importantly, they suggest that the surface above the FAD cofactor in CYB5R3, which is accessible to the known partner Cyt‐b5 in the ER membrane, is also accessible to RSAD2. Further investigations are required to determine whether the composition of the ER membrane influences electron transfer between CYB5R3 and RSAD2 and subsequent antiviral effects. For example, we cannot rule out the possibility that a specific lipid component during inflammation may facilitate the interaction between these proteins and the electron‐transfer process.

### 2.10 Predicting the Transient Complex Between CYB5R3 and RSAD2

After identifying the CYB5R3 surface that interacts with RSAD2, we aimed to determine the structure of the transient complex between CYB5R3 and RSAD2, a task that is not easily achievable using X‐ray crystallography or Cryo‐EM. Therefore, we used advanced computational methods to predict the complex between the two proteins. The X‐ray crystal structure of mouse RSAD2 [30, 53] is solved, allowing us to use AlphaFold and AlphaFill in combination with molecular dynamics (MD) simulations to predict the structure of human RSAD2 (hSAND). We predicted with high confidence the energy‐optimised structure of human RSAD2 in complex with SAM and CTP (Figure S20). Based on the predicted structure of human RSAD2, the solved structure of human CYB5R3 [52], and our identification of CYB5R3 surface interacting with RSAD2 (Figure 5a–d), we performed multiple docking experiments combined with MD simulations (Figure S21 and Tables S4,S5). The result yielded a model with a shortest distance of approximately 20 Å between the CYB5R3 FAD and the RSAD2 [4Fe‐4S] cluster (Figure 5e). This distance is consistent with the electron‐tunnelling distance of approximately 20 Å between biological cofactors along electron transfer chains [54]. The predicted model is similar to the complex predicted by AlphaFold Multimer (Figure S22). In the predicted complex between RSAD2 and CYB5R3, the CTP H4′ is pointed towards the SAM C5′ position, approximately 3 Å away (Figure S20). This observation is consistent with the distance observed in the X‐ray crystal structure of mouse RSAD2 solved in the presence of CTP [30]. Therefore, it confirms the accuracy of our predicted structures. The predicted surface of RSAD2 interacting with CYB5R3 is available for interactions when considering the orientation of the N‐terminus amphipathic domain of RSAD2 and its localisation to the ER membrane (Figure S23).

### 2.11 Structural Features Important for Electron Transfer

The predicted RSAD2/CYB5R3 complex (Figure 5e) aligns with the NMR‐detected surface of CYB5R3 that interacts with RSAD2. Specifically, residues 276–281 in a short α‐helix and the C‐terminal segment of CYB5R3 (Arg297 and Phe301) identified by NMR spectroscopy (Figure 5b) directly face the Q_195_GKK loop of RSAD2 (Figure 5e and Figure S24). The Q195GKK loop of RSAD2 directly interacts with FAD. Specifically, the Gln195 forms hydrogen bonds with the CYB5R3 C‐terminal carboxylate Phe301, and the two lysine residues (Lys197 and Lys198) of this loop form hydrogen bonds with FAD (Figure 5e). These interactions can lead to conformational changes and chemical shift perturbations in its surrounding residues, namely Met127, Leu131, and Lys120, as observed by NMR (Figure 5b). Additionally, the structure revealed that a highly conserved tyrosine (Tyr85) is located 4.1 Å above the RSAD2 [4Fe‐4S] cluster and is 15.2 Å from the flavin of CYB5R3. These distances suggested a role of Tyr85 in long‐range electron tunnelling (Figure 5e). The Q_195_GKK surface loop and Tyr85 are highly conserved in vertebrates (Figure S25). Additionally, we noted that analogous loops with aromatic amino acid residues, which facilitate long‐range electron tunnelling, are present in several other human radical‐SAM enzymes (Figures S26,S27). The Q_195_GKK loop and Tyr85 are absent in TtSAND (Figure S28), which cannot receive electrons from CYB5R3 (Figure 3e). These interactions are absent in the predicted complex between RSAD2 and CYB5R1, R2, and R4 (Figures S29,S30).

### 2.12 Mutations of the Q_195_GKK Loop or Tyr85 Affect Electron Transfer

**FIGURE 6:**
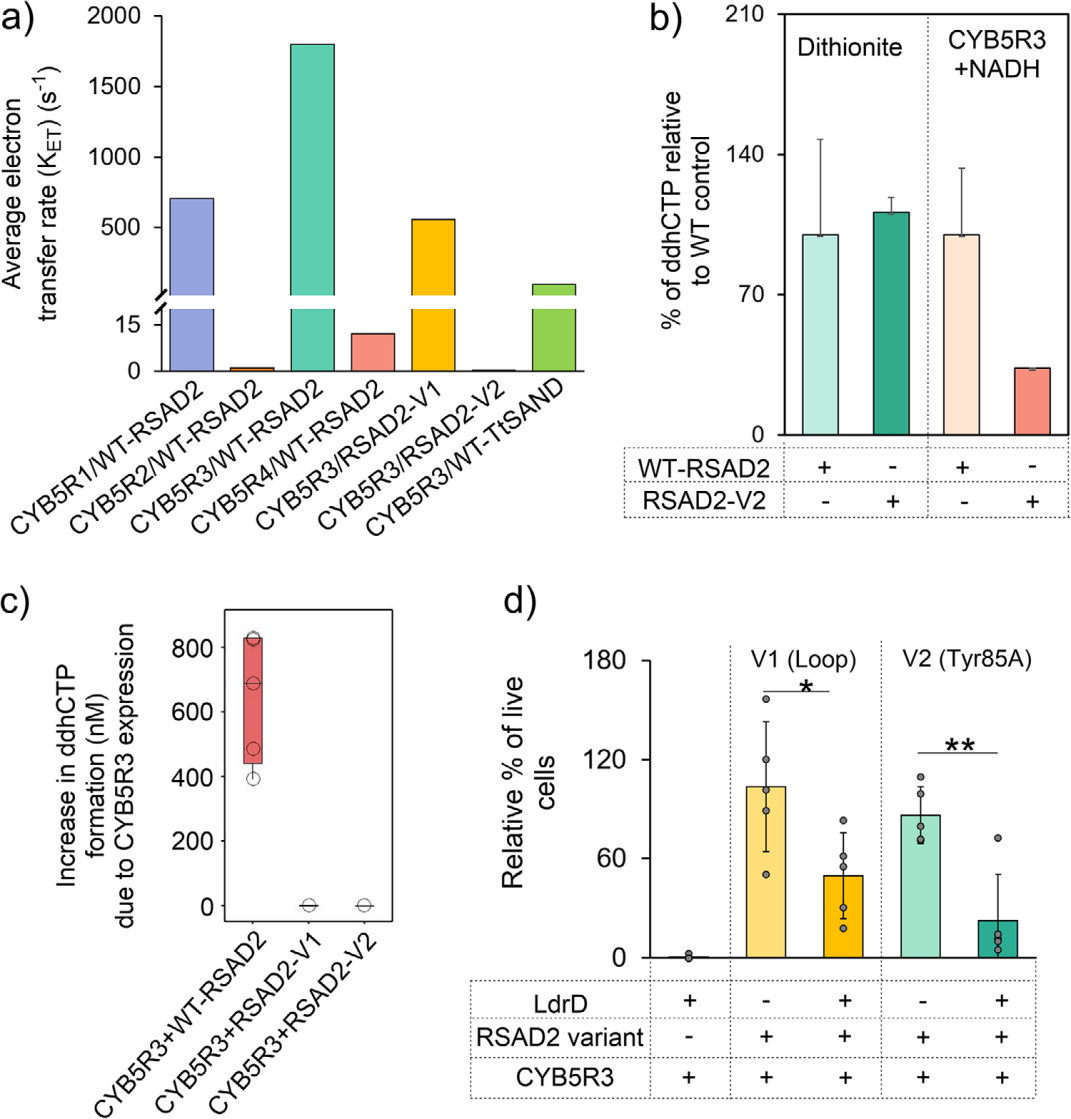
The Q195GKK loop and Tyr85 in RSAD2 are required for electron transfer from the CYB5R3 FAD cofactor to the RSAD2 [4Fe‐4S]^2+^ cluster. (a) The mutation of the loop and tyrosine abolishes the predicted electron‐transfer rate. Computationally predicted average electron transfer rates (r^−1^) for each CYB5R in complex with RSAD2, for CYB5R3 in complex with RSAD2‐V1 or RSAD2‐V2, and for TtSAND in complex with CYB5R3. (b) Relative % of ddhCTP formation for WT‐RSAD2 and RSAD2‐V2 (Tyr85A) in the presence of dithionite as reducing agent or CYB5R3 and NADH as electron donor. The concentration of wild‐type (WT) or Tyr85A variant (V2) was 18 µM. Concentrations of CTP, SAM, and sodium dithionite were 5.3, 21.2, and 5.3 mM, respectively. The final buffer was 50 mM MOPS buffer, pH 7.0, containing 100 mM NaCl. Data are the average of two (*n* = 2) independent measurements ± errors. (c) The RSAD2 surface loop (RSAD2‐V1) and Tyr85A variants (RSAD2‐V2) were unable to receive electrons from CYB5R3 inside cells. Targeted metabolomics was used to detect an increase in ddhCTP levels resulting from co‐expression of CYB5R3 and an RSAD2 variant in *E. coli*. For visualisation and comparison, the increase in ddhCTP formation resulting from co‐expression of WT‐RSAD2 and CYB5R3 (data from Figure 3a) is plotted alongside. Data are an average of five (*n* = 5) biologically independent samples ± standard deviation. (d) The RSAD2 Q_195_GKK loop variant (V1) and Tyr85A variant (V2) cannot receive electrons from CYB5R3 to efficiently produce ddhCTP and fully rescue the growth of *E. coli* cells, unlike wild‐type (WT) RSAD2 (Figure 3c). (c,d) Data are an average of five (*n* = 5) biologically independent samples ± standard deviation and are corrected for differences in the expression level of proteins. One‐way ANOVA analysis was used to calculate *p* values. *p value ≤ 0.05; **p value ≤ 0.01; ***p value ≤ 0.001.

To test if Q_195_GKK and Tyr85 are important for electron transfer, we created two variants of RSAD2: Variant 1 (RSAD2‐V1), in which two lysine residues of the Q_195_GKK loop were replaced with aspartic acid and valine, respectively, and Variant 2 (RSAD2‐V2), in which Tyr85 was replaced with an alanine (Supplementary Methods). We predicted their complex with CYB5R3 (Figure S31). In the RSAD2‐V1/CYB5R3 complex, the interaction of the loop with the CYB5R3 FAD cofactor is partially disrupted (Figure 5f), while in the CYB5R3/RSAD2‐V2 complex, it is entirely altered (Figure 5g). Consistently, the calculated electron coupling and electron transfer rate (*k_ET_*) (Supplementary Methods) for the CYB5R3/RSAD2‐V1 complex decreased by 30%, while those for the CYB5R3/RSAD2‐V2 complex were negligible (Figure 6a and Table S6). The lower electron transfer rate (*k_ET_*) of RSAD2‑V1 and RSAD2‑V2 with CYB5R3‐compared to wild‑type RSAD2 with CYB5R3‐mirrors the low rates seen for other predicted complexes, none of which support efficient electron transfer inside cells, unlike the wild‐type RSAD2/CYB5R3 pair (Figure 3a).

To further validate the results of predictions, we overexpressed and purified the soluble forms of RSAD2‐V1 and RSAD2‐V2. The catalytic activity of RSAD2‐V1 and RSAD2‐V2 was the same as that of WT‐RSAD2 when the chemical reducing agent dithionite was used (Figure 6b and Figure S32). Therefore, mutations did not affect the RSAD2 cluster function. The in vitro activity of CYB5R3/RSAD2‐V1 was the same as that of CYB5R3/WT‐RSAD2 (Figure S32). In contrast, the activity of CYB5R3/RSAD2‐V2 to generate ddhCTP in vitro decreased by more than 70% (Figure 6b). We then measured activity inside cells. We first quantified the expression of WT‐RSAD2, RSAD2‐V1, and RSAD2‐V2 in *E. coli* using Western blot analysis (Figure S33). Subsequently, targeted metabolomics measurements of ddhCTP formation were performed, and data were corrected for relative protein expression (Figure 6c). The results revealed that both RSAD2‐V1 and RSAD2‐V2 failed to efficiently receive electrons from CYB5R3 inside cells and increase ddhCTP synthesis beyond detection limit (Figure 6c), unlike wild‐type RSAD2 (Figure 3a). Consistently, the VITAS assay showed that the chain‐termination activity of both variants in the presence of CYB5R3 (Figure 6d) did not fully rescue *E. coli* growth, unlike wild‐type RSAD2 (Figure 3c). Thus, both variants lost the ability to efficiently receive electrons from human CYB5R3 inside cells. These findings together confirm that the RSAD2 Q_195_GKK surface loop acts as a molecular plug, facilitating electron transfer from the reduced CYB5R3 FADH_2_ to the oxidised RSAD2 [4Fe‐4S]^2+^ cluster through the conserved Tyr85. This electron transfer process activates catalytic synthesis of ddhCTP.

### 2.13 Creating iCAREs to Bypass the Need for Co‐Localisation

**FIGURE 7:**
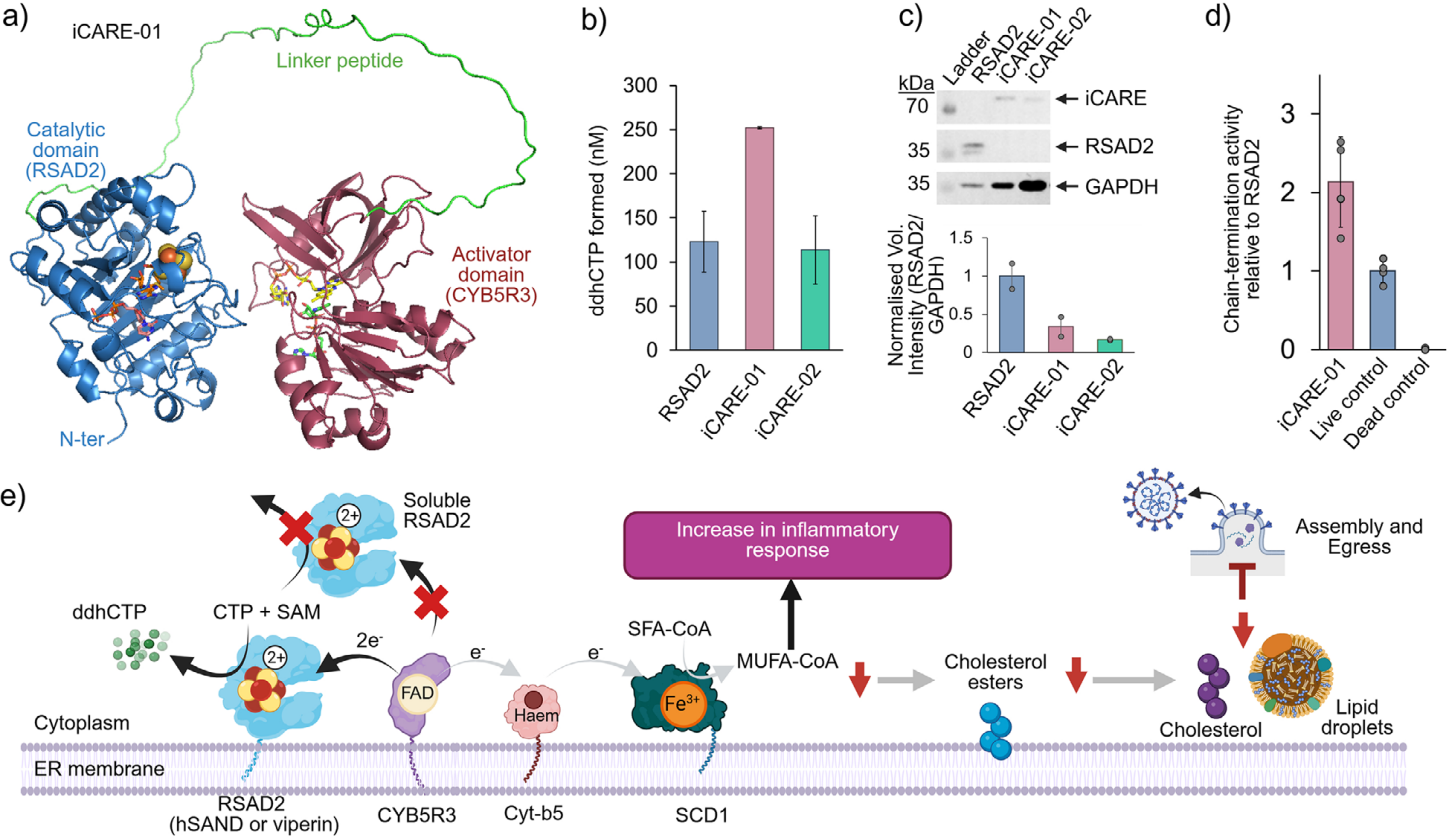
Creation of iCAREs. (a) The AlphaFold predicted structure of iCARE01, which was created by linking the C‐terminus of RSAD2 (catalytic domain) to the N‐terminus of CYB5R3 (activator domain). (b) The amount (nM) of ddhCTP formation by iCARE‐01 and iCARE‐02 as compared to wild‐type RSAD2 (14 µM) in the presence of CYB5R3 (14 µM). The final concentrations of iCARE‐01 and iCARE‐02 were 25 µM, and those of CTP, SAM, and NADH were 1.5, 2, and 2 mM, respectively. Experiments were repeated at least twice using different protein batches to confirm reproducibility. FAD (yellow). (c) Western blot analysis of iCAREs expression as compared to RSAD2. *E. coli*. The top 10 cells were transfected with plasmids encoding RSAD2, iCARE‐01, or iCARE‐02. An overlay of the Chemi‐blot and the ladder image is shown at the top, and the bands are quantified relative to the GAPDH control. The results from two expressions were quantified. Relative volumetric intensities were used to correct the VITAS assay results. (b–c) Data are the average of two (*n* = 2) biologically independent measurements ± errors. (d) Chain termination activity of iCARE‐01 relative to that measured in cells expressing RSAD2 and CYB5R3 together with LdrD (live control) or cells expressing only LdrD (dead control). The data are the average of four (*n* = 4) biologically independent measurements ± standard deviation (SD). (e) The RSAD2 is co‐localised with CYB5R3 via its N‐terminus amphipathic domain. Removal of this domain blocks electron transfer and antiviral activity. Electron transfer from CYB5R3 enables transformation of CTP to ddhCTP. RSAD2 expression and interaction with CYB5R3 may interfere with Cyt‐b5 interaction with CYB5R3. This interference may reduce electron flow to stearoyl‐CoA desaturase (SCD1). This enzyme is the rate‐limiting step in fatty acid biosynthesis. It catalyses the conversion of saturated fatty acids (SFA) to monounsaturated fatty acids (MUFA), both of which are required for cholesterol metabolism. Hence, RSAD2 activity can indirectly reduce cholesterol metabolism as reported, thereby restricting viral replication, as observed for several viruses.

The naturally or genetically fused self‐sufficient enzymes are powerful biocatalysts for various applications [1, 5, 6, 55]. Despite the significant potential of radical‐SAM enzymes for biocatalysis, they have not been engineered to create self‐sufficient enzymes. To fill this gap, we used our discovery to create iCAREs as a proof‐of‐concept self‐sufficient enzymes for antiviral therapy. We used a non‐immunogenic peptide naturally used to fuse the FAD and Cyt‐b5 domains of CYB5R4. Two designs were created. In one design, the C‐terminus of RSAD2 is fused with the N‐terminus of CYB5R3 (iCARE‐01), and in another design, the N‐terminus of RSAD2 is fused with the C‐terminus of CYB5R3 (iCARE‐02). The predicted structure of iCAREs (Figure 7a and Figure S34) showed that the interaction surface between the two domains is consistent with that observed in the RSAD2‐CYB5R3 complex (Figure 5). Purification of the enzymes and their characterisation by UV‐visible spectrophotometry, compared with soluble RSAD2 and CYB5R3, confirmed that both enzymes contain a [4Fe‐4S] cluster with a shoulder extending to 700 nm and an FAD cofactor with peaks at 391, 460, and 485 nm (Figure S35). Analysis of iron content (Figure S35) confirmed that both iCAREs have similar [4Fe‐4S] cluster content to wild‐type RSAD2. Hence, engineering iCAREs did not affect FeS cluster and FAD binding to the catalytic domain (RSAD2) and the activator domain (CYB5R3), respectively. Consistently, both iCAREs were active in vitro in the presence of NADH (Figure 7b). After correction for the relative protein expression levels (Figure 7c and Figure S36) (Equation 3, Supplementary methods), both enzymes showed improved chain‐termination activity as compared to live control, when RSAD2 and CYB5R3 were co‐expressed in the presence of LdrD, and dead control, cells only expressing LdrD (Figure 7d and Figure S35). Therefore, by fusing the two proteins, we bypassed the need for co‐localisation and increased the number of encounters required for efficient electron transfer in transient complexes [44, 45].

## 3 Conclusion

We demonstrated that CYB5R3 is a putative ETP of RSAD2 (hSAND). Our data show direct electron transfer from CYB5R3 to RSAD2, a mechanism distinct from that involving a reductase and an electron‑carrier protein, as proposed for bacterial radical‑SAM enzymes [38]. We found that the RSAD2 Q195GKK surface loop and Tyr85 participate in electron tunnelling from FADH_2_ to the [4Fe‑4S]^2^^+^ cluster. Importantly, we show that co‑localisation at the ER membrane is required for this electron transfer, providing a mechanistic explanation for the observation by several groups that the N‑terminal amphipathic region of RSAD2 is essential for antiviral activity [16, 32, 33] and for inhibiting T7 RNA polymerase activity [34]. The N‑terminal region enables RSAD2 to co‑localise with CYB5R3 at the ER membrane, thereby receiving electrons to synthesise ddhCTP (Figure 7e), which can block viral RNA polymerases or modulate immunometabolism [22].

Building on this discovery, we created iCAREs—the first example of catalytically self‑sufficient radical‑SAM enzymes. These autonomous enzymes are candidates for mRNA or gene delivery, for example, using Adeno‑Associated Virus (AAV) [56, 57]. Because iCAREs do not require recruitment of endogenous CYB5R3, they are predicted to avoid the unintended interference associated with RSAD2 overexpression and are thus likely to have a favourable safety profile relative to constitutive RSAD2, which requires recruitment of its putative ETP, CYB5R3. This recruitment could perturb the CYB5R3 interaction with Cyt‑b5, as we observed in vitro, thereby affecting electron flow to stearoyl‑CoA desaturase (SCD1), the rate‑limiting enzyme in MUFA biosynthesis. Such interference would disrupt lipid metabolism, an essential cellular process. This prediction is consistent with evidence that cholesterol metabolism and lipid droplets are essential for viral particle assembly and budding [58, 59] and that RSAD2 overexpression disrupts lipid and cholesterol metabolism [23, 32, 60]. While constitutive RSAD2 overexpression is driven by inflammation in the tumour microenvironment and the enzyme may contribute to oncometabolism [61], genome‑wide association studies have not identified RSAD2 as a driver oncogene. However, SCD1 has been identified as an oncogene [62, 63], and CYB5R3 can have both cancer‑promoting and tumour‑suppressing roles [37, 64]. Our findings thus raise the possibility that constitutive RSAD2 overexpression could indirectly contribute to tumorigenesis by interfering with other biological functions of CYB5R3.

A limitation of our work is that it does not provide definite experimental evidence that CYB5R3 is the sole ETP of RSAD2 within cells. Further investigations are needed to determine whether other ETPs in the ER membrane can supply electrons to RSAD2. Additionally, future studies are required to test whether members of the CYB5R family can donate electrons to other radical‑SAM enzymes, such as Elp3 [65] and Dph1‑Dph2 [66]. Finally, rigorous preclinical safety and efficacy studies are required to establish iCARE's therapeutic suitability.

## Author Contributions

**Mengdi Wu**: formal analysis, writing – review and editing, writing – original draft, visualization, data collection and curation, funding acquisition. **Nghi Thao Hoang**: data collection and curation, formal analysis, writing – review and editing, writing – original draft, visualization, funding acquisition. **Deborah Grifagni**: data collection and curation, formal analysis, writing – review and editing, writing – original draft, visualization. **Meritxell Wu‐Lu**: data collection and curation, formal analysis, writing – review and editing, writing – original draft, visualization. **Yujie Sheng**: data collection and curation. **Theo Situmorang**: data collection and curation, formal analysis. **Pei‐Hsin Tai**: data collection and curation. **Zheng Chen**: data collection and curation. **Astrid Maluta**: data collection and curation. **Mohammed Hakil**: data collection and curation. **Bianca Susini**: data collection and curation. **Hannah Florance**: data collection and curation, methodology, formal analysis, software. **Peter‐Leon Hagedoorn**: supervision, resources, data curation, writing – review and editing, investigation. **J. Mark Sutton**: supervision. **Maria‐Andrea Mroginski**: resources, supervision, data curation, formal analysis, project administration, writing – review and editing, visualization, validation, investigation, funding acquisition, writing – original draft, methodology. **Simone Ciofi‐Baffoni**: investigation, data curation, funding acquisition, writing – original draft, writing – review and editing, visualization, validation, formal analysis, project administration, resources, supervision, data curation, methodology. **Kourosh H. Ebrahimi**: conceptualization, investigation, data curation, funding acquisition, writing – original draft, writing – review and editing, project administration, supervision, resources, visualization, validation, formal analysis, methodology.

## Conflicts of Interest

KHE has filed a patent application based on the discoveries described in this article.

## Supporting information



The authors have cited additional references within the Supporting Information [1, 2, 3, 4, 5, 6, 7, 8, 9, 10, 11, 12, 13, 14, 15, 16, 17, 18, 19, 20, 21, 22, 23, 24, 25, 26, 27, 28, 29, 30, 31, 32, 33, 34]. **Supporting File**: anie73650‐sup‐0001‐SuppMat.pdf.

## Data Availability

The data that support the findings of this study are available from the corresponding author upon reasonable request.
