# Supplementary material for: Discovery of an Antiviral Electron Transfer Process to Create Catalytically Self‐Sufficient Viral Restriction Factors
Source: Angew Chem Int Ed Engl. 2026 Jul 21;65(39):e5423408. doi: 10.1002/anie.5423408 (PMC13592299; doi:10.1002/anie.5423408)
Supplement: Supplementary file 1 [file ANIE-65-e5423408-s001.pdf]

Discovery of an Antiviral Electron Transfer Process to Create Catalytically Self-Sufficient Viral Restriction Factors

Mengdi Wu[a]†, Nghi Thao Hoang[a,b]†, Deborah Grifagni[c]†, Meritxell Wu-Lu[d]†, Yujie Sheng[a], Theo Situmorang[a], Pei-Hsin Tai[a], Zheng Chen[a], Astrid Maluta[a], Mohammed Hakil[a], Bianca Susini[c], Hannah Florance[e], Peter-Leon Hagedoorn[f], J. Mark Sutton[a,g], Maria-Andrea Mroginski[d], Simone Ciofi-Baffoni[c], Kourosh H. Ebrahimi[a]*

Dedication † These authors contributed equally to this work.

[a] Ms M. Wu, Ms N. T. Hoang, Mr. Y. Sheng, Mr. T. Situmorang, Ms. P-H. Tai, Mr. Z. Chen, Ms. A. Maluta, Mr. M. Hakil, Prof. J. M. Sutton, Dr K. H. Ebrahimi
Department: Institute of Pharmaceutical Science,
Institution: King's College London
Address: 150 Stamford Street, London, SE1 9NH, United Kingdom
Correspondence: Kourosh.ebrahimi@kcl.ac.uk

[b] Ms. N. T. Hoang,
Department: Department of Pharmacy
Institution: Da Nang University of Medical Technology and Pharmacy
Address: 99 Hùng Vương, Hải Châu, Đà Nẵng 550000, Vietnam

[c] Dr D. Grifagni, Ms. B. Susini, Prof. S. Ciofi-Baffoni
Department: Magnetic Resonance Center and Department of Chemistry
Institution: University of Florence
Address: Via Sacconi, 6, 50019 Sesto Fiorentino, Florence, Italy

[d] Dr M. Wu-Lu, Prof M. A. Mroginski,
Department: Department of Chemistry
Institution: Technical University of Berlin
Address: Chemiegebäude, Str. des 17. Juni 115, 10623 Berlin, Germany

[e] Dr H. Florance
Institution: Agilent Technologies
Address: Cheadle, UK

[f] Dr P. L. Hagedoorn
Department: Department of Biotechnology
Institution: Delft University of Technology
Address: Gebouw 58, Van der Maasweg 9, 2629 HZ Delft, The Netherlands

[g] Prof J. M. Sutton
Institution: UK Health Security Agency
Address: Manor Farm Road, Porton Down, Salisbury, SP4 0JG, United Kingdom

Supplementary Methods

Chemicals and reagents. All chemicals were reagent grade and purchased from Sigma or Thermo Fisher. Enterokinase from the porcine intestine (40 UN) was from Merck (E0885-40UN) and prepared as explained previously.[1]

Cloning and overexpression of proteins (RSAD2 (hSAND), TtSAND, CYB5R1-5, and Cyt-b5) in E. coli. The cloning of human SAND (RSAD2) and the fungal TtSAND was described before.[2,3] The *E. coli* codon-optimized synthetic genes for RSAD2-V1, RSAD2-V2, all CYB5R members and Cyt-b5 were purchased from GeneArt (Life Science Tech.). In the RSAD2-V1 variant, two lysine residues of Q_{195}GKK were replaced by aspartic acid and valine. These mutations were done since, in the fungal TtSAND, these lysine residues are replaced by aspartic acid and valine. The synthesized gene for RSAD2-V1 or RSAD2-V2 was cloned into pBAD His/C plasmid using Kpn-I and EcoRI restriction enzymes. The genes of all CYB5R members were cloned into the pCYC-Duet-1 expression vector using EcoRI and HindIII restriction enzymes. The synthetic gene of Cyt-b5 had a C-terminus His-tag and was cloned into pCYC-Duet-1 expression vector using NdeI and KpnI restriction sites. The presence of the correct insert was confirmed using restriction enzyme double digestion. RSAD2, its variants, and TtSAND were expressed as described previously for TtSAND and RSAD2.[2,3] Briefly, the TOP10 competent cells were transformed with the pBAD His/C plasmid expressing a protein. Cells were spread on an LB-Agar plate containing 100 µg/ml ampicillin and incubated overnight at 37 °C. The next day, a single colony was picked and inoculated in 50 ml LB media supplemented with 100 µg/ml ampicillin. Cells were grown overnight in a MAXQ6000 shaker (ThermoFisher) (37 °C and 185 rpm) for approximately 18 hours and subsequently inoculated in a 2 L flask containing 500 ml TB medium. After the OD at 600 nm reached a value between 0.5 - 0.8, expression of the protein was induced by the addition of 0.04% (W/V) arabinose (final concentration), and the growth was continued (37 °C and 200 rpm) for another 6-7 hours. Finally, cells were collected by centrifugation (Megafuge ST Plus Series, Thermo Scientific) at 4696 x *g* at 4 °C for 20 minutes and stored in a -80 °C freezer. For the expression of each CYB5R or Cyt-b5 enzyme, BL21 *E. coli* cells were transformed with the pCYC-Duet-1 plasmid encoding a protein. Cells were spread on LB-agar plates supplemented with 25 µg/ml chloramphenicol. The plates were incubated at 37 °C overnight. The next day, a single colony was picked up and inoculated into a small flask containing 50 mL LB medium and 25 µg/ml chloramphenicol. The culture was incubated in a shaker/incubator overnight at 37 °C and 200 rpm for approximately 18 hours. The next day, the overnight culture was inoculated into a 2 L flask containing 500 ml TB medium. Protein production was induced by the addition of 1 mM (final concentration) isopropyl β-d-1-thiogalactopyranoside (IPTG) when the OD_{600} reached 0.5 - 0.8. Subsequently, the cells were grown for an additional 6-7 hours and then collected by centrifugation at 4696 x *g* at 4 °C for 20 minutes. Collected cells were stored at -80 °C. The ^{13}C,^{15}N-labelled CYB5R3 protein was expressed in M9 minimal medium containing 3.5 g of ^{13}C-glucose and 1.2 g of ^{15}N ammonium sulfate, with the same plasmid and cellular line of the protein expressed in TB medium. Cell growth was performed at 37°C. When the culture reached an OD at 600 nm of 0.5-0.8, 1 mM isopropyl IPTG was added to induce protein overexpression, and the cells were incubated at 18°C overnight. The lysis and purification steps were performed as described below for the CYB5R proteins.The RSAD2 constructs lack the N-terminus hydrophobic domain, residues 1-68. The CYB5R1 construct lacks the N-terminus hydrophobic domain, residues 1-32. The CYB5R3 construct lacks the N-terminus hydrophobic domain, residues 1-27.

Cloning and overexpression of synthetic iCAREs. The *E. coli* codon-optimised genes encoding iCAREs were purchased from GeneArt (Thermo Fisher). These synthetic genes were subcloned into the pBAD/His C vector using the KpnI and HindIII restriction sites. After cloning, the identity of the correct insert was confirmed by double enzyme restriction digestion and subsequently by DNA sequencing. Proteins were expressed as explained above for RSAD2.

DNA sequences

E. coli RSAD2 wild-type
*CAGCTAGCATGCCGACCACACCGACCAGCGTTAATTATCATTTTACCCGTCAGTGCAACTACA
AATGCGGTTTTTGTTTTCATACCGCCAAAACCAGCTTTGTTCTGCCGCTGGAAGAAGCAAAAC
GCGGTCTGCTGCTGCTGAAAGAAGCCGGTATGGAAAAGATTAACTTTAGCGGTGGTGAACCG
TTTCTGCAGGATCGTGGTGAATATCTGGGTAAACTGGTTCGTTTTTGCAAAGTTGAACTGCGT
CTGCCGAGCGTTAGCATTGTTAGCAATGGTAGCCTGATTCGTGAACGTTGGTTTCAGAATTAT
GGCGAGTATCTGGATATTCTGGCAATTAGCTGTGATAGCTTTGATGAAGAAGTGAACGTTCTG
ATTGGTCGTGGTCAGGGTAAAAAGAATCATGTTGAAAACCTGCAAAAACTGCGTCGTTGGTGT
CGTGATTATCGTGTTGCCTTTAAAATCAACAGCGTGATCAATCGCTTTAACGTGGAAGAAGATA
TGACCGAGCAGATTAAAGCACTGAATCCGGTTCGTTGGAAAGTTTTTCAGTGTCTGCTGATTG
AAGGTGAAAATTGTGGTGAAGATGCACTGCGTGAAGCAGAACGTTTTGTTATTGGTGATGAAG
AGTTTGAACGTTTTCTGGAACGTCATAAAGAAGTTAGCTGTCTGGTTCCGGAAAGCAACCAGA
AAATGAAAGATTCCTATCTGATCCTGGATGAATACATGCGTTTTCTGAATTGCCGTAAAGGTCG
TAAAGATCCGAGCAAAAGCATTCTGGATGTTGGTGTTGAAGAAGCCATCAAATTTAGCGGTTT
CGACGAGAAAATGTTTCTGAAACGTGGTGGCAAATACATTTGGAGCAAAGCAGATCTGAAACT
GGATTGGTAA*

E. coli RSAD2 loop variant (RSAD2-V1)
*CAGCTAGCATGCCGACCACACCGACCAGCGTTAATTATCATTTTACCCGTCAGTGCAACTACA
AATGCGGTTTTTGTTTTCATACCGCCAAAACCAGCTTTGTTCTGCCGCTGGAAGAAGCAAAAC
GCGGTCTGCTGCTGCTGAAAGAAGCCGGTATGGAAAAGATTAACTTTAGCGGTGGTGAACCG
TTTCTGCAGGATCGTGGTGAATATCTGGGTAAACTGGTTCGTTTTTGCAAAGTTGAACTGCGT
CTGCCGAGCGTTAGCATTGTTAGCAATGGTAGCCTGATTCGTGAACGTTGGTTTCAGAATTAT
GGCGAGTATCTGGATATTCTGGCAATTAGCTGTGATAGCTTTGATGAAGAAGTGAACGTTCTG
ATTGGTCGTGGTCAGGGTGATGTTAATCATGTTGAAAATCTGCAAAAACTGCGTCGTTGGTGT
CGTGATTATCGTGTTGCCTTTAAAATCAACAGCGTGATCAATCGCTTTAACGTGGAAGAAGATA
TGACCGAGCAGATTAAAGCACTGAATCCGGTTCGTTGGAAAGTTTTTCAGTGTCTGCTGATTG
AAGGTGAAAATTGTGGTGAAGATGCACTGCGTGAAGCAGAACGTTTTGTTATTGGTGATGAAG
AGTTTGAACGTTTTCTGGAACGTCATAAAGAAGTTAGCTGTCTGGTTCCGGAAAGCAACCAGA
AAATGAAAGATTCCTATCTGATCCTGGATGAATACATGCGTTTTCTGAATTGCCGTAAAGGTCG
TAAAGATCCGAGCAAAAGCATTCTGGATGTTGGTGTTGAAGAAGCCATCAAATTTAGCGGTTT
CGACGAGAAAATGTTTCTGAAACGTGGTGGCAAATACATTTGGAGCAAAGCAGATCTGAAACT
GGATTGGTAA*

E. coli RSAD2 Tyr85A variant (RSAD2-V2)
*ATGCCGACCACACCGACCAGCGTTAATTATCATTTTACCCGTCAGTGTAATGCCAAATGCGGTT
TTTGTTTTCATACCGCCAAAACCAGCTTTGTTCTGCCGCTGGAAGAAGCAAAACGCGGTCTGC
TGCTGCTGAAAGAAGCCGGTATGGAAAAGATTAACTTTAGCGGTGGTGAACCGTTTCTGCAGG
ATCGTGGTGAATATCTGGGTAAACTGGTTCGTTTTTGCAAAGTTGAACTGCGTCTGCCGAGCG
TTAGCATTGTTAGCAATGGTAGCCTGATTCGTGAACGTTGGTTTCAGAATTATGGCGAGTATCTG
GATATTCTGGCAATTAGCTGTGATAGCTTTGATGAAGAAGTGAACGTTCTGATTGGTCGTGGTC
AGGGTAAAAAGAATCATGTTGAAAACCTGCAAAAACTGCGTCGTTGGTGTCGTGATTATCGTGT
TGCCTTTAAAATCAACAGCGTGATCAATCGCTTTAACGTGGAAGAAGATATGACCGAGCAGATT
AAAGCACTGAATCCGGTTCGTTGGAAAGTTTTTCAGTGTCTGCTGATTGAAGGTGAAAATTGTG
GTGAAGATGCACTGCGTGAAGCAGAACGTTTTGTTATTGGTGATGAAGAGTTTGAACGTTTTCT
GGAACGTCATAAAGAAGTTAGCTGTCTGGTTCCGGAAAGCAACCAGAAAATGAAAGATTCCTAT
CTGATCCTGGATGAATACATGCGTTTTCTGAATTGCCGTAAAGGTCGTAAAGATCCGAGCAAAA
GCATTCTGGATGTTGGTGTTGAAGAAGCCATCAAATTTAGCGGTTTCGACGAGAAAATGTTTCT
GAAACGTGGTGGCAAATACATTTGGAGCAAAGCAGATCTGAAACTGGATTGG*

E. coli CYB5R1
*CGAATTCGATGAGCCGTCGTCCGCAGGTTACCCTGCTGGATCCGAATGAAAAATATCTGCTG
CGTCTGCTGGATAAAACCACCGTTAGCCATAATACCAAACGTTTTCGTTTTGCACTGCCGACC*

*GCACATCACACCCTGGGTCTGCCGGTTGGTAAACATATTTATCTGAGCACCCGTATTGATGGT
AGCCTGGTTATTCGTCCGTATACACCGGTTACCAGTGATGAAGATCAGGGTTATGTGGATCTG
GTGATTAAAGTGTATCTGAAAGGTGTGCATCCGAAATTTCCGGAAGGTGGTAAAATGAGCCAG
TATCTGGATAGCCTGAAAGTTGGTGATGTTGTTGAATTTCGTGGTCCGAGCGGTCTGCTGACC
TATACCGGTAAAGGCCATTTTAACATTCAGCCGAATAAAAAGAGCCCTCCGGAACCGCGTGTT
GCAAAGAAACTGGGTATGATTGCCGGTGGCACCGGTATTACCCCGATGCTGCAGCTGATTCG
TGCAATTCTGAAAGTGCCGGAAGATCCGACACAGTGTTTTCTGCTGTTTGCAAATCAGACCGA
GAAAGATATTATTCTGCGTGAGGATCTGGAAGAACTGCAGGCACGTTATCCGAATCGTTTTAA
ACTGTGGTTTACCCTGGATCATCCGCCTAAAGATTGGGCATATAGCAAAGGTTTTGTTACCGC
AGATATGATCCGTGAACATCTGCCTGCACCGGGTGATGATGTTCTGGTTCTGCTGTGTGGTCC
GCCTCCGATGGTTCAGCTGGCATGTCATCCGAATCTGGATAAACTGGGTTATAGCCAGAAAAT
GCGCTTCACCTATTAA*

***E. coli* CYB5R2**
*CGAATTCGATGAATAGCCGTCGTCGTGAACCGATTACACTGCAGGATCCGGAAGCAAAATATC
CGCTGCCGCTGATTGAAAAAGAGAAAATTAGCCATAATACCCGTCGCTTTCGTTTTGGTCTGC
CGAGTCCGGATCATGTTCTGGGTCTGCCGGTTGGTAATTATGTTCAGCTGCTGGCAAAAATCG
ATAATGAACTGGTTGTTCGTGCATATACACCGGTTAGCAGTGATGATGATCGTGGTTTTGTTG
ATCTGATCATCAAGATCTACTTCAAAAATGTGCATCCGCAGTATCCTGAAGGTGGTAAAATGAC
CCAGTATCTGGAAAACATGAAAATCGGCGAAACCATTTTCTTTCGTGGTCCGCGTGGTCGTCT
GTTTTATCATGGTCCGGGTAATCTGGGTATTCGTCCTGATCAGACCAGCGAACCGAAAAAGAC
CCTGGCAGATCATCTGGGCATGATTGCCGGTGGCACCGGTATTACCCCGATGCTGCAGCTGA
TTCGTCATATTACCAAAGATCCGAGCGATCGTACCCGTATGAGCCTGATTTTTGCAAATCAGA
CCGAAGAAGATATCCTGGTTCGTAAAGAACTGGAAGAAATTGCACGTACCCATCCGGATCAGT
TTAATCTGTGGTATACCCTGGATCGTCCGCCTATTGGTTGGAAATATAGCAGCGGTTTTGTTA
CCGCAGATATGATCAAAGAACATCTGCCTCCGCCTGCAAAAAGCACCCTGATTCTGGTTTGTG
GTCCGCCTCCGCTGATTCAGACCGCAGCACATCCGAACCTGGAAAAACTGGGTTATACCCAG
GATATGATCTTCACCTATTAA*

***E. coli* CYB5R3**
*CGAATTCGATGCAGCGTAGCACACCGGCAATTACCCTGGAAAGTCCGGATATCAAATATCCG
CTGCGTCTGATTGATCGTGAAATTATCAGCCATGATACCCGTCGTTTTCGTTTTGCACTGCCG
AGTCCGCAGCATATTCTGGGTCTGCCGGTTGGTCAGCATATTTATCTGAGCGCACGTATTGAT
GGTAATCTGGTTGTTCGTCCGTATACACCGATTAGCAGTGATGATGATAAAGGCTTTGTTGAT
CTGGTGATCAAGGTGTATTTCAAAGATACGCATCCGAAATTTCCGGCAGGCGGTAAAATGAGC
CAGTATCTGGAAAGCATGCAGATTGGTGATACCATTGAATTTCGTGGTCCGAGCGGTCTGCTG
GTTTATCAAGGTAAAGGTAAATTTGCAATCCGTCCGGATAAAAAGAGCAATCCGATTATTCGTA
CCGTTAAAAGCGTTGGTATGATTGCTGGCGGTACAGGTATTACCCCGATGCTGCAGGTTATTC
GTGCAATTATGAAAGATCCTGATGATCATACCGTTTGCCATCTGCTGTTTGCAAATCAGACCG
AAAAAGATATTCTGCTGCGTCCGGAACTGGAAGAACTGCGTAATAAACATAGCGCACGTTTCA
AACTGTGGTATACCCTGGATCGTGCACCGGAAGCATGGGATTATGGTCAGGGTTTTGTTAAC
GAAGAAATGATCCGTGATCATCTGCCTCCGCCTGAAGAAGAACCGCTGGTTCTGATGTGTGG
TCCGCCTCCGATGATTCAGTATGCATGTCTGCCGAATCTGGATCATGTTGGTCATCCGACCGA
ACGTTGTTTTGTGTTTTAA*

***E. coli* CYB5R4**
*CGAATTCGATGCTGAATGTTCCGAGCCAGAGCTTTCCGGCACCGCGTAGCCAGCAGCGTGTT
GCAAGCGGTGGTCGTAGCAAAGTTCCGCTGAAACAGGGTCGTAGCCTGATGGATTGGATTCG
TCTGACCAAAAGCGGTAAAGATCTGACCGGTCTGAAAGGTCGTCTGATTGAAGTTACCGAAGA
AGAACTGAAAAAGCACAACAAAAAGGATGATTGCTGGATTTGCATTCGCGGTTTTGTTTATAAT
GTGAGCCCGTACATGGAATATCATCCTGGTGGTGAAGATGAACTGATGCGTGCAGCAGGTAG
TGATGGCACCGAACTGTTTGATCAGGTTCATCGTTGGGTTAACTATGAGAGCATGCTGAAAGA
ATGTCTGGTTGGTCGTATGGCAATTAAACCGGCAGTTCTGAAAGATTATCGTGAGGAAGAAAA
GAAAGTTCTGAATGGTATGCTGCCGAAAAGCCAGGTTACCGATACACTGGCAAAAGAAGGTC
CGAGCTATCCGAGTTATGATTGGTTTCAGACAGATAGCCTGGTTACCATTGCCATTTATACCAA
ACAGAAAGACATCAACCTGGACAGCATTATTGTGGATCATCAGAATGATAGCTTTCGTGCCGA*

*AACCATTATCAAAGATTGCCTGTATCTGATTCATATCGGTCTGAGCCATGAAGTGCAAGAGGA
TTTTAGCGTTCGTGTTGTTGAAAGCGTGGGCAAAATTGAAATTGTGCTGCAGAAGAAAGAAAA
CACCAGTTGGGATTTTCTGGGTCACCCGCTGAAAAACCATAATAGCCTGATTCCGCGTAAAGA
TACCGGTCTGTATTATCGTAAATGTCAGCTGATTAGCAAAGAGGATGTTACCCACGATACCCG
TCTGTTTTGTCTGATGCTGCCTCCGAGCACACATCTGCAGGTTCCGATTGGTCAGCATGTTTA
TCTGAAACTGCCGATTACCGGCACCGAAATTGTTAAACCGTATACACCGGTTAGCGGTAGCCT
GCTGAGCGAATTTAAAGAACCTGTTCTGCCGAACAACAAATATATCTACTTCCTGATCAAGATC
TATCCGACGGGTCTGTTTACACCGGAACTGGATCGTCTGCAGATTGGTGATTTTGTTAGCGTT
AGCAGTCCGGAAGGCAATTTCAAAATTAGCAAATTCCAAGAGCTTGAGGACCTGTTTCTGCTG
GCAGCAGGCACCGGTTTTACCCCGATGGTTAAAATTCTGAATTATGCCCTGACCGATATTCCG
AGCCTGCGTAAAGTTAAACTGATGTTCTTCAATAAAACCGAGGACGATATTATCTGGCGTAGT
CAGCTGGAAAAACTGGCCTTTAAAGATAAACGTCTGGACGTGGAATTTGTTCTGAGCGCACCG
ATTAGCGAATGGAATGGCAAACAGGGCCATATTAGTCCGGCACTGCTGTCAGAATTTCTGAAA
CGTAATCTGGACAAAAGCAAAGTGCTGGTTTGTATTTGTGGTCCGGTTCCGTTTACCGAACAG
GGTGTTCGTCTGCTGCATGATCTGAATTTTAGCAAAAACGAAATCCACAGCTTTACCGCCTAA*

Human expression gene RSAD2 wild-type
*CAGCTAGCATGCCCACCACACCTACCAGCGTGAACTACCACTTCACCCGGCAGTGCAACTAC
AAGTGCGGCTTCTGCTTCCACACCGCCAAGACCAGCTTTGTGCTGCCTCTGGAAGAGGCCAA
GAGAGGCCTGCTGCTGCTGAAAGAGGCCGGCATGGAAAAGATCAACTTCTCTGGCGGCGAG
CCCTTTCTGCAGGACAGAGGCGAGTATCTGGGAAAGCTCGTGCGGTTCTGCAAGGTGGAACT
GAGACTGCCCAGCGTGTCCATCGTGTCCAATGGCAGCCTGATCAGAGAGCGGTGGTTCCAG
AACTACGGCGAGTACCTGGACATCCTGGCCATCAGCTGCGACAGCTTCGACGAGGAAGTGAA
CGTCCTGATCGGCAGAGGCCAGGGCAAGAAAAACCACGTGGAAAATCTGCAGAAACTGCGG
CGGTGGTGCCGGGATTACAGAGTGGCCTTCAAGATCAACAGCGTGATCAACCGGTTCAACGT
GGAAGAGGACATGACCGAGCAGATCAAGGCCCTGAATCCTGTGCGGTGGAAGGTGTTCCAG
TGCCTGCTGATCGAGGGCGAGAATTGTGGCGAGGATGCCCTGAGAGAAGCCGAGAGATTTG
TGATCGGCGACGAAGAGTTCGAGCGGTTCCTGGAACGGCACAAAGAGGTGTCCTGTCTGGT
CCCCGAGAGCAACCAGAAGATGAAGGACAGCTACCTGATCCTGGACGAGTACATGCGGTTTC
TGAACTGCCGGAAGGGCAGAAAGGACCCCAGCAAGAGCATCCTGGATGTGGGCGTTGAGGA
AGCCATCAAGTTCAGCGGCTTCGATGAGAAGATGTTCCTGAAGAGAGGCGGCAAGTACATCT
GGTCCAAGGCCGACCTGAAGCTGGATTGGTAG*

Human Expression gene CYB5R3
*CGAATTCGATGCAGCGTAGCACACCGGCAATTACCCTGGAAAGTCCGGATATCAAATATCCG
CTGCGTCTGATTGATCGTGAAATTATCAGCCATGATACCCGTCGTTTTCGTTTTGCACTGCCG
AGTCCGCAGCATATTCTGGGTCTGCCGGTTGGTCAGCATATTTATCTGAGCGCACGTATTGAT
GGTAATCTGGTTGTTCGTCCGTATACACCGATTAGCAGTGATGATGATAAAGGCTTTGTTGAT
CTGGTGATCAAGGTGTATTTCAAAGATACGCATCCGAAATTTCCGGCAGGCGGTAAAATGAGC
CAGTATCTGGAAAGCATGCAGATTGGTGATACCATTGAATTTCGTGGTCCGAGCGGTCTGCTG
GTTTATCAAGGTAAAGGTAAATTTGCAATCCGTCCGGATAAAAAGAGCAATCCGATTATTCGTA
CCGTTAAAAGCGTTGGTATGATTGCTGGCGGTACAGGTATTACCCCGATGCTGCAGGTTATTC
GTGCAATTATGAAAGATCCTGATGATCATACCGTTTGCCATCTGCTGTTTGCAAATCAGACCG
AAAAAGATATTCTGCTGCGTCCGGAACTGGAAGAACTGCGTAATAAACATAGCGCACGTTTCA
AACTGTGGTATACCCTGGATCGTGCACCGGAAGCATGGGATTATGGTCAGGGTTTTGTTAAC
GAAGAAATGATCCGTGATCATCTGCCTCCGCCTGAAGAAGAACCGCTGGTTCTGATGTGTGG
TCCGCCTCCGATGATTCAGTATGCATGTCTGCCGAATCTGGATCATGTTGGTCATCCGACCGA
ACGTTGTTTTGTGTTTTAA*

Protein sequences

Wild-type RSAD2 in E. coli
MPTTPTSVNYHFTRQCNYKCGFCFHTAKTSFVLPLEEAKRGLLLLKEAGMEKINFSGGEPFLQDR
GEYLGKLVRFCKVELRLPSVSIVSNGSLIRERWFQNYGEYLDILAISCDSFDEEVNVLIGRGQGKKN

HVENLQKLRRWCRDYRVAFKINSVINRFNVEEDMTEQIKALNPVRWKVFQCLLIEGENCGEDALR
EAERFVIGDEEFERFLERHKEVSCLVPESNQKMKDSYLILDEYMRFLNCRKGRKDPSKSILDVGVE
EAIKFSGFDEKMFLKRGGKYIWSKADLKLDW

RSAD2-V1 in *E. coli*
MPTTPTSVNYHFTRQCNYKCGFCFHTAKTSFVLPLEEAKRGLLLLKEAGMEKINFSGGEPFLQDR
GEYLGKLVRFCKVELRLPSVSIVSNGSLIRERWFQNYGEYLDILAISCDSFDEEVNVLIGRGQGDVN
HVENLQKLRRWCRDYRVAFKINSVINRFNVEEDMTEQIKALNPVRWKVFQCLLIEGENCGEDALR
EAERFVIGDEEFERFLERHKEVSCLVPESNQKMKDSYLILDEYMRFLNCRKGRKDPSKSILDVGVE
EAIKFSGFDEKMFLKRGGKYIWSKADLKLDW

RSAD2-V2 in *E. coli*
MPTTPTSVNYHFTRQCNAKCGFCFHTAKTSFVLPLEEAKRGLLLLKEAGMEKINFSGGEPFLQDR
GEYLGKLVRFCKVELRLPSVSIVSNGSLIRERWFQNYGEYLDILAISCDSFDEEVNVLIGRGQGKKN
HVENLQKLRRWCRDYRVAFKINSVINRFNVEEDMTEQIKALNPVRWKVFQCLLIEGENCGEDALR
EAERFVIGDEEFERFLERHKEVSCLVPESNQKMKDSYLILDEYMRFLNCRKGRKDPSKSILDVGVE
EAIKFSGFDEKMFLKRGGKYIWSKADLKLDW

CYB5R3 in E. coli
MQRSTPAITLESPDIKYPLRLIDREIISHDTRRFRFALPSPQHILGLPVGQHIYLSARIDGNLVVRPYT
PISSDDDKGFVDLVIKVYFKDTHPKFPAGGKMSQYLESMQIGDTIEFRGPSGLLVYQGKGKFAIRPD
KKSNPIIRTVKSVGMIAGGTGITPMLQVIRAIMKDPDDHTVCHLLFANQTEKDILLRPELEELRNKHS
ARFKLWYTLDRAPEAWDYGQGFVNEEMIRDHLPPPEEEPLVLMCGPPPMIQYACLPNLDHVGHPT
ERCFVF

CYB5R1 in E. coli
MSRRPQVTLLDPNEKYLLRLLDKTTVSHNTKRFRFALPTAHHTLGLPVGKHIYLSTRIDGSLVIRPY
TPVTSDEDQGYVDLVIKVYLKGVHPKFPEGGKMSQYLDSLKVGDVVEFRGPSGLLTYTGKGHFNI
QPNKKSPPEPRVAKKLGMIAGGTGITPMLQLIRAILKVPEDPTQCFLLFANQTEKDIILREDLEELQA
RYPNRFKLWFTLDHPPKDWAYSKGFVTADMIREHLPAPGDDVLVLLCGPPPMVQLACHPNLDKL
GYSQKMRFTY

CYB5R2 in E. coli
MNSRRREPITLQDPEAKYPLPLIEKEKISHNTRRFRFGLPSPDHVLGLPVGNYVQLLAKIDNELVVR
AYTPVSSDDDRGFVDLIIKIYFKNVHPQYPEGGKMTQYLENMKIGETIFFRGPRGRLFYHGPGNLGI
RPDQTSEPKKTLADHLGMIAGGTGITPMLQLIRHITKDPSDRTRMSLIFANQTEEDILVRKELEEIAR
THPDQFNLWYTLDRPPIGWKYSSGFVTADMIKEHLPPPAKSTLILVCGPPPLIQTAAHPNLEKLGYT
QDMIFTY

CYB5R4 in E. coli
MLNVPSQSFPAPRSQQRVASGGRSKVPLKQGRSLMDWIRLTKSGKDLTGLKGRLIEVTEEELKKH
NKKDDCWICIRGFVYNVSPYMEYHPGGEDELMRAAGSDGTELFDQVHRWVNYESMLKECLVGR
MAIKPAVLKDYREEEKKVLNGMLPKSQVTDTLAKEGPSYPSYDWFQTDSLVTIAIYTKQKDINLDSII
VDHQNDSFRAETIIKDCLYLIHIGLSHEVQEDFSVRVVESVGKIEIVLQKKENTSWDFLGHPLKNHN
SLIPRKDTGLYYRKCQLISKEDVTHDTRLFCLMLPPSTHLQVPIGQHVYLKLPITGTEIVKPYTPVSG
SLLSEFKEPVLPNNKYIYFLIKIYPTGLFTPELDRLQIGDFVSVSSPEGNFKISKFQELEDLFLLAAGT
GFTPMVKILNYALTDIPSLRKVKLMFFNKTEDDIIWRSQLEKLAFKDKRLDVEFVLSAPISEWNGKQ
GHISPALLSEFLKRNLDKSKVLVCICGPVPFTEQGVRLLHDLNFSKNEIHSFTA

Cyt-b5 in *E. coli*
MAEQSDEAVKYYTLEEIQKHNHSKSTWLILHHKVYDLTKFLEEHPGGEEVLREQAGGDATENFED
VGHSTDAREMSKTFIIGELHPDDRPKLNKPPETLITTIDSSDKLAAALEHHHHHH

RSAD2 expression in human

MPTTPTSVNYHFTRQCNYKCGFCFHTAKTSFVLPLEEAKRGLLLLKEAGMEKINFSGGEPFLQDR
GEYLGKLVRFCKVELRLPSVSIVSNGSLIRERWFQNYGEYLDILAISCDSFDEEVNVLIGRGQGKKN
HVENLQKLRRWCRDYRVAFKINSVINRFNVEEDMTEQIKALNPVRWKVFQCLLIEGENCGEDALR
EAERFVIGDEEFERFLERHKEVSCLVPESNQKMKDSYLILDEYMRFLNCRKGRKDPSKSILDVGVE
EAIKFSGFDEKMFLKRGGKYIWSKADLKLDW

CYB5R3 expression in human
MQRSTPAITLESPDIKYPLRLIDREIISHDTRRFRFALPSPQHILGLPVGQHIYLSARIDGNLVVRPYT
PISSDDDKGFVDLVIKVYFKDTHPKFPAGGKMSQYLESMQIGDTIEFRGPSGLLVYQGKGKFAIRPD
KKSNPIIRTVKSVGMIAGGTGITPMLQVIRAIMKDPDDHTVCHLLFANQTEKDILLRPELEELRNKHS
ARFKLWYTLDRAPEAWDYGQGFVNEEMIRDHLPPPEEEPLVLMCGPPPMIQYACLPNLDHVGHPT
ERCFVF

Purification of RSAD2, RSAD2-V1, RSAD2-V2, and TtSAND. Purification of TtSAND was carried out as explained previously.[3] For human RSAD2 (hSAND) and its variants (RSAD2-V1 and RSAD2-V2) the cell pellet was removed from -80 °C freezer. Cell lysis was performed as explained before.[4] Briefly, cells were transferred to an anaerobic glovebox (COY Laboratories) and added to centrifuge tubes. The pellet was thawed, and cells were resuspended in lysis buffer (50 mM Tris pH 8.0, 300 mM NaCl, 2% Triton X-100, 0.5 mM DTT, 0.05 mg/ml DNase, 0.05 mg/ml PMSF (phenylmethylsulfonyl fluoride)) containing lysozyme (2 mg/mL). Fully resuspended cells were lysed on ice using a Branson SFX 150 Sonifier (Emerson). The settings were power 30%, 10 cycles, each 30 seconds on, followed by 10 seconds off. Each time, 5 ml of resuspended cells was lysed to ensure homogeneous lysis. After centrifugation at 4696 x *g* at 4 °C for 40 minutes, the supernatant containing soluble proteins was collected and immediately transferred to an anaerobic glovebox (COY Laboratories) (O_2 < 5 ppm). Anaerobic purification of RSAD2, RSAD2-V1, RSAD2-V2 or TtSAND was achieved using a gravity column packed with Ni^{2+}-resin (ThermoFisher Scientific). The following buffers were used: Equilibration buffer (50 mM Tris, 300 mM NaCl, 2% Triton X-100, 5 mM Imidazole, pH 7.5); Wash buffer (50 mM Tris, 300 mM NaCl, 2%v/v Triton X-100, 0.5 mM DTT, 10 mM Imidazole, pH 7.5); and Elution buffer (50mM Tris, 300 mM NaCl, 2 %v/v Triton X-100, 0.5 mM DTT, 500 mM Imidazole, pH 7.5). Subsequently, the PD10 desalting column (Cytiva) was used to exchange the buffer and remove Imidazole and DTT. The final buffer was 50 mM MOPS buffer, pH 7.0, containing 100 mM NaCl. The purified proteins were aliquoted into 1 ml and flash-frozen and stored in liquid nitrogen. Protein purity was confirmed by 10% SDS-PAGE, and protein concentration was measured by the BCA assay. The concentration of RSAD2 was between 90 and 100 µM unless otherwise stated. Protein concentrations are the average of three independent measurements ± 15% error.

Purification of CYB5R proteins. BL21 cells overexpressing a CYB5R member were removed from the freezer and thawed on ice at room temperature. Cells were resuspended in lysis buffer. The lysis buffer was 50 mM Tris-Base, 300 mM NaCl, 2% (v/v) Triton X-100 and 2.0 mg/ml lysozyme (Thermo Fisher Scientific) at pH 8.0 with the addition of 0.05 mg/ml Phenylmethylsulphonyl Fluoride (PMSF) to block protease activity and 0.05 mg/ml DNase (Sigma Aldrich). The latter was added after pH adjustment. Cells were lysed, each time 5 ml of cells in a 50 ml Falcon tube, as explained for RSAD2. Next, lysed cells were centrifuged at 4696 × g at 4 °C for 40 minutes to remove cell debris, and the supernatant containing soluble proteins was collected. Purification of the CYB5R proteins was performed aerobically using His-tag affinity chromatography. A gravity column (Thermo Fisher Scientific) was packed with His-Pur™ Ni-NTA resin (Thermo Fisher Scientific), 500 µL of resin per 500 mL of bacterial culture. First, the resin was washed with equilibration buffer (50 mM Tris-Base, 300 mM NaCl, 10 mM Imidazole, 0.2% v/v Triton X-100 at pH 7.5) (5 steps, twice the volume of the resin). Then, the resin was mixed with a supernatant containing a CYB5R protein, and the mixture was incubated using a rotating mixer at 4 °C for 2 hours. This solution was then poured into the gravity column and washed with wash buffer (50 mM Tris-Base, 300 mM NaCl, 50 mM Imidazole, 0.2% v/v Triton X-100 at pH 7.5) for 5 times the resin volume. At the end, each protein was eluted with an elution buffer (50 mM Tris-Base, 300 mM NaCl, 500 mM Imidazole, 0.2% v/v Triton X-100, pH 7.5). Finally, the elution buffer was exchanged into 50 mM Tris at pH 7.5, containing 300 mM

NaCl, using a PD10 desalting column (Cytiva). All purification steps were performed under aerobic conditions. The purity of the proteins was confirmed using SDS-PAGE (SureCast™ Handcast System, Thermo Fisher), and the proteins' concentrations were determined using the BCA Protein Assay Kit (Millipore).

Purification of iCAREs. The purification of synthetic iCAREs was performed under anaerobic conditions similar to RSAD2, and the buffer did not contain DDT. After the PD10 desalting column, protein concentration was determined by BCA assay, and proteins were flash-frozen in liquid nitrogen, aliquoted into 1.5 mL Eppendorf tubes, and stored in liquid nitrogen for subsequent studies.

UV-visible spectrophotometry and enzyme kinetics measurements of CYB5R members. The concentrations of the purified CYB5R proteins were measured using the BCA assay. For enzyme kinetic analysis, the buffer was 50 mM MOPS buffer, pH 7.0, containing 300 mM NaCl, pH 7.0. DCIP (Dichlorophenolindophenol) was used as an artificial electron acceptor. The molar extinction coefficient of $DCIP_{OX}$ ($\varepsilon_{600\ nm}$) 22,000 M^{-1} cm^{-1} was used to calculate the concentration of reduced DCIP in various NADH concentrations based on the Lambert-Beer law. Samples for kinetic studies consisted of a stock solution of DCIP (5 mM), a stock solution of CYB5R (1.14 µM), and freshly prepared various NADH concentrations (0.1 – 30 mM). All materials were kept on ice during measurements. The 1 mL reaction consisted of 992 µL PD-10 column buffer (50 mM MOPS-300 mM NaCl pH 7.0), 2 µL DCIP, 5 µL NADH, and an aliquot of CYB5R to reach a final concentration of 0.0014 (CYB5R1-3) or 0.0048 µM (CYB5R4). The measurements with the UV-visible spectrophotometer were set on TimeDrive mode (continuous measurement) for 5 minutes at 600 nm using a LAMBDA 365 UV/VIS spectrophotometer (PerkinElmer). Initially, a mixture of DCIP and NADH in 50 mM MOPS, pH 7.0, containing 300 mM NaCl, was measured for 60 seconds to ensure baseline stability. Subsequently, the cuvette was removed, and a CYB5R member was added; the solution was mixed quickly by inverting the cuvette twice. After approximately 10 seconds, the cuvette was returned to the spectrophotometer, and progress curves of DCIP reduction (decrease in absorbance at 600 nm) were recorded. For each NADH concentration, measurements were repeated three times. The initial slope of the progress curve (circa 50 seconds) was used to obtain the initial rate and enzyme unit (µmol of DCIP formed per min per mg of enzyme). The results were plotted as a function of NADH concentration, and OriginLab and Microsoft Excel were used to obtain the best fit to the data using the Michaelis-Menten equation.

Pull-down assay and Western blot analysis. His-Ent-RSAD2 (RSAD2 with an enterokinase cleavage N-terminus 6x-His tag) was subject to enterokinase cleavage inside an anaerobic glovebox (O_2 < 5 ppm). The RSAD2 stock was diluted to a final concentration of 20 or 15 µM using anaerobic buffer, 50 mM MOPS, 100 mM NaCl, pH 7.0. Next, 10 µl of the stock solution of enterokinase was added to 1 ml of RSAD2 solution, and the mixture was incubated overnight at room temperature inside the anaerobic glovebox (O_2 < 5 ppm). The next day, 100 µl of Ni-resin was washed five times, each time with 200 µl of anaerobic buffer (50 mM MOPS, 100 mM NaCl, pH 7.0). A stock solution of a CYB5R was thawed in the glove box and diluted to a concentration of 20 µM using anaerobic MOPS buffer (50 mM MOPS, 100 mM NaCl, pH 7.0). Next, 500 µl of a CYB5R (assay) or buffer (control) (50 mM MOPS, 100 mM NaCl, pH 7.0) was added to the resin, and the solution was incubated for 1 hour. After incubation, the resin was added to a gravity column and washed ten times, each time with 500 µl of buffer (50 mM MOPS, 100 mM NaCl, pH 7.0) to remove any unbound CYB5R. Subsequently, 300 µl of the overnight enterokinase-treated RSAD2 was added to the column, and the mixture was incubated for another 1 hour under anaerobic conditions. The resin was then washed 10 times, each time with 500 µl of buffer (50 mM MOPS, 100 mM NaCl, pH 7.0). The first and the last washes were collected for further analysis. Finally, the proteins were eluted using an elution buffer (50 mM Tris, 300 mM NaCl, 500 mM Imidazole, 0.2% Triton, pH 7.5). The samples collected after the first and last washes, as well as the elution, were subjected to Western blot analysis.

Western blot of protein expression and imaging. Either Novex 8-16% Tris-Glycine Plus WedgeWell Gel (Invitrogen) was used, or 12% SDS-PAGE gels were prepared using the SureCast Gel Handcast System (Invitrogen), according to the manufacturer's protocol. Two ladders were used: 1-3 µl of MagicMark™ XP Western Protein Standard (Thermo Fisher) and 3-5 µl of PageRuler™ Plus Pre-stained Protein Ladder (Thermo Fisher), 10 µl of sample and control were loaded into the gel. Following electrophoresis, the proteins were transferred to a PVDF Membrane (Thermo Fisher) using the Power Blotter system (Thermo Fisher). Antibody incubation was performed using the iBind Flex system (Thermo Fisher). The primary antibodies were RSAD2 Rabbit polyclonal Antibody (Proteintech) (1:1000), as confirmed previously using RSAD2 knockout cells,[5] CYB5R3 rabbit polyclonal antibody (Proteintech) (1:1000) (as confirmed using purified protein), HRP-conjugated 6x-His Tag Monoclonal Antibody (Invitrogen, MA1-21315-HRP) (1:1000), GAPDH HRP-conjugated monoclonal antibody (GA1R) (Invitrogen, MA5-15738-HRP) (1:1000). When secondary antibody was used, it was Anti-rabbit IgG HRP-linked Antibody (Cell Signalling) (1:2000). The Clarity Western ECL Substrate was used for staining. The membrane was imaged using the iBright imaging system (ThermoFisher), and quantification was performed with iBright software.

In vitro activity measurements of RSAD2 or TtSAND. An anaerobic buffer (50 mM MOPS, pH 7.0, containing 100 mM NaCl) was used to prepare stock solutions of SAM, CTP, and sodium dithionite. An aliquot of chemicals was transported into the glovebox and mixed with the buffer under anaerobic conditions. The buffer was prepared a day prior to the experiments and stored in the glovebox overnight to ensure complete removal of dioxygen. All samples were prepared under anaerobic conditions in a glovebox (O_2 < 5 ppm) at room temperature. The reaction consists of a mixture of CYB5R (12.5-28 µM), RSAD2 (28-38 µM), NADH (5.6-14 µM), SAM (11-14 mM), and CTP (2.8-3.5 mM). A positive control sample was prepared in which CYB5R and NADH were replaced with sodium dithionite (2.8 mM), and the volume was adjusted to match the complete reaction volume using anaerobic buffer (50 mM MOPS, pH 7.0, containing 100 mM NaCl). Two negative control samples were also prepared: in one, RSAD2, SAM, and CTP were omitted; in the other, RSAD2 and CYB5R were omitted. The volume of the negative controls was adjusted to that of the complete reaction using anaerobic buffer (50 mM MOPS, pH 7.0, containing 100 mM NaCl). We replaced RSAD2 with TtSAND in the reaction and controls and used UTP instead of CTP to test CYB5R3 activity with TtSAND. The complete reaction and control samples were incubated anaerobically overnight (circa 17-18 hours). The following day, 450 µl of each sample was added to a 0.5 ml Amicon Ultra Centrifugal Filter (3 kDa) (Merck). The samples were centrifuged under aerobic conditions at 16250 x *g* for 30 minutes. The resulting flow-through was then carefully transferred to a recovery HPLC vial (Fisher Scientific) and sealed tightly with a polypropylene cap (Fisher Scientific). All samples were immediately analysed by LC-MS after preparation. Each experiment was repeated at least twice with different protein batches to assess reproducibility.

In vitro activity of iCAREs. The *in vitro* activity of ***iCAREs*** was measured by LC-MS and samples were prepared as described above for RSAD2. Four reactions (125 µl, final volume) were set up. Two complete reactions, each containing iCARE-01 or iCARE-02 (30 µM), CTP (1.5 mM), SAM (2 mM), and NADH (2 mM); A control reaction containing RSAD2 (14 µM) and CYB5R3 (14 µM), CTP (1.5 mM), SAM (2 mM), and NADH (2 mM); and a negative control lacking the enzymes but containing all other components.

Metabolomic studies using *E. coli* cells. Metabolites were extracted from three conditions. *E. coli* BL21-AI were transformed with a plasmid expressing RSAD2 only, a plasmid expressing a CYB5R member only, or two plasmids, one expressing RSAD2 and one expressing CYB5R. Transformation was performed according to the Thermo Fisher Scientific protocol. After transformation, cells were spread on the LB-agar plates containing either ampicillin (100 µg/ml) (RSAD2) or chloramphenicol (25 µg/ml) (CYB5R transformed cells) or ampicillin (100 µg/ml) and chloramphenicol (25 µg/ml) (RSAD2/CYB5R double-transformed cells). A single colony from each condition was picked and inoculated into 5 ml LB medium containing 100 µg/ml ampicillin (RSAD2), 25 µg/ml chloramphenicol (CYB5R), or 100 µg/ml ampicillin and 25 µg/ml chloramphenicol (RSAD2 and CYB5R). The glass vials were incubated in a shaker at 37 °C and 185 rpm for 24 hours. Subsequently, the mini culture was inoculated in 50 ml LB medium, and immediately 0.04% (final concentration) of L-arabinose

(RSAD2), 1 mM (final concentration) IPTG (CYB5R), or 1 mM (final concentration) IPTG and 0.04% (final concentration) of L-arabinose (CYBB5R and RSAD2) was added. This culture was incubated at 37 °C in a shaker at 185 rpm overnight. The following day, cells were collected by centrifugation at 4696 x *g* at 4 °C for 10 minutes. The medium was discarded, and cells were washed twice with 50 mL ice-cold Tris buffer (50 mM Tris, 150 mM NaCl, pH 7.4), then centrifuged at 4696 x *g* at 4 °C for 10 minutes to remove the buffer. After discarding the buffer, cells were stored in a -80 °C freezer. To extract metabolites, cells were thawed on ice, and 500 µl of dry-ice cold 100% methanol was added to the cell pellet in a 50 ml Falcon tube and mixed well. Sonication was then performed on ice for 2 minutes with 40% amplitude, each cycle lasted for 10 seconds and was followed by a 10-second break. After sonication, three cycles of 10 minutes of freeze and 10 minutes of thaw were performed using dry ice. Subsequent steps were done on ice. Lysed cells were added to 1.5 ml Eppendorf tubes and centrifuged for 10 minutes at 16250 x *g* to remove cell debris and denatured proteins. After centrifugation, the supernatants were added to new 1.5 ml tubes, and the protein concentration was measured using the BCA assay. The concentrations of RSAD2 and RSAD2/CYB5R samples were adjusted to be the same. All samples were filtered with the Amicon Ultra-15 Centrifugal Filter (3 kDa MWCO) from Millipore (Sigma-Aldrich), and the flowthrough from each sample was collected and added to HPLC recovery vials for LC-MS analysis. Samples were analysed using an Agilent InfinityLab G6160A LC/MSD iQ mass spectrometer, as explained below. OpenLab CDS software was used to analyse data. The area under the extracted ion chromatograms (EICs) for ddhCTP $[M-H]^{-1}$, m/z of 464.0, was used to calculate the fold change in ddhCTP formation. Equation 1 was used to estimate the amount of ddhCTP generated and correct this amount based on the relative expression of a CYB5R as determined using western blot analysis.

$$Fold\ change = \left(\frac{A_S - Ave.A_{CYB5R}}{Ave.A_{RSAD2}}\right) / \left(\text{relative vol. intensity } \left(\frac{\text{CYB5R}}{\text{CYB5R3}}\right)\right) \quad (1)$$

In which

- A_S: is the area under the EICs for each sample obtained from cells expressing CYB5R alone, RSAD2 alone, or both RSAD2 and a CYB5R member.
- $Ave.A_{CYB5R}$: is the average of the area under the EICs for all samples obtained from cells expressing a CYB5R alone.
- $Ave.A_{RSAD2}$: is the average area under EICs for all samples obtained from cells expressing RSAD2 alone.
- Relative vol. intensity (CYB5R/CYB5R3): is the intensity of the western blot band for the expression of a CYB5R member relative to the intensity of the western blot band for the expression of CYB5R3.

Expression of RSAD2 and CYB5R3 in HEK293 cells. The cells were obtained internally for another research group. The human codon-optimized synthetic gene encoding a soluble form of CYB5R3 and RSAD2, lacking the N-terminal hydrophobic domain, cloned into pcDNA 3.1(+), was purchased from GeneArt (Thermo Fisher). These synthetic plasmids were used for the transfection of human embryonic kidney (HEK293) cells. These cells were cultured in a T75 flask using Dulbecco's Modified Eagle Medium (1X) (DMEM; Thermo Fisher Scientific) supplemented with 1 mM L-glutamine (Gibco), 10% fetal bovine serum (FBS; Gibco), and 1% penicillin-streptomycin (Pen/Strep; Thermo Fisher Scientific). Cells were incubated at 37°C in a humidified incubator with 5% CO_2 until they reached approximately 80-90% confluency. The cells were then split and transferred into four T25 flasks, each receiving fresh media to a total volume of 5 ml. The seeding density was adjusted to 0.7×10^6 cells per T25 flask. The cells were incubated at 37°C for 24 hours to allow them to adhere and divide. The following day, cell confluency was assessed, ensuring that the cells covered approximately 50-60% of each T25 flask. Two hours before transfection, the old medium was replaced with fresh DMEM (Thermo Fisher Scientific) medium supplemented with 1 mM L-glutamine, 10% FBS, and 1% Pen/Strep. Transfection was performed using Lipofectamine 3000 Transfection Kit (Thermo Fisher Scientific) as described below:

- Reagent A: 16.5 µl of Lipofectamine™ 3000 (Thermo Fisher Scientific) was diluted in 250 µl of Opti-MEM™ (Thermo Fisher Scientific). The solution was mixed thoroughly by pipetting up and down.
- Reagent B: For cells transfected with one plasmid (either expressing RSAD2 or CYB5R3), a master mix of 14 µg of the plasmid DNA was diluted in 250 µl of Opti-MEM™. To this, 22 µl of P3000™ reagent (Thermo Fisher Scientific) was added, and the solution was mixed thoroughly by pipetting. For cells transfected with both plasmids, 14 µg, each plasmid DNA was added to the solution.

Reagent A (250 µl) was combined with Reagent B (250 µl), and the mixture was incubated at room temperature for 15 minutes. After incubation, 500 µl of the reagent mixture was added to each T25 flask. Three conditions were prepared:

- RSAD2-only expression: Transfected with a plasmid encoding RSAD2.
- CYB5R3-only expression: Transfected with a plasmid encoding CYB5R3.
- Double expression: Co-transfected with plasmids encoding RSAD2 and CYB5R3.

After adding transfection reagents, cells were incubated for 24 hours at 37°C in a 5% CO_2 incubator. After 24 hours, cells were transferred into four T75 flasks, each receiving fresh medium to a final volume of 15 ml. The cells were then incubated for an additional 24 hours at 37°C in a humidified incubator with 5% CO_2. Transfection efficiency was monitored using GFP and was circa 60-70%. HEK293T cells were incubated for 48 hours post-transfection, and metabolites were extracted as described below.

Preparation of metabolomic samples using HEK293 cells. The cell culture medium was carefully removed from each flask. Next, 25 mL of phosphate-buffered saline (PBS; Gibco) at pH 7.5 was added to each flask. The flasks were gently shaken by hand to wash out any residual media and remove non-adherent cells. The PBS was then removed. Immediately after washing, 25 ml of liquid nitrogen was added to each flask to rapidly freeze the cells. Next, after approximately 1 minute, when the liquid nitrogen had evaporated, 350 µl of ice-cold 100% methanol was added to each flask. An L-shaped cell spreader was used to carefully scrape the frozen cells from the flask surface. The cell suspension was then transferred into pre-chilled vials using a pipette. These vials were immediately placed on ice. The collected cell suspensions were centrifuged at 16250 x *g* for 10 minutes at 4°C. Following centrifugation, the supernatant was carefully transferred into new pre-labelled vials for further analysis. The DNA concentration in each vial was quantified using a NanoDrop spectrophotometer (IMPLEN). Then, an aliquot of 100% methanol was used to adjust the DNA concentration of each sample to that of the sample with the lowest concentration. Next, 350 µl of each sample was added to a 0.5 mL Amicon Ultra Centrifugal Filter 3 kDa (Merck) and centrifuged at 16250 x *g* for 30 minutes at room temperature. The resulting flow-through was then carefully transferred to a recovery HPLC vial (Fisher Scientific) and sealed tightly with a Polypropylene cap (Fisher Scientific). The samples were stored at -80°C and later analysed by HR LC-MS.

Liquid Chromatography-Mass spectrometry (LC-MS). Measurements were performed using an Agilent InfinityLab G6160A LC/MSD iQ mass spectrometer equipped with an Agilent 1260 Infinity II liquid chromatography system. Instrument control and data processing were performed using OpenLab CDS. The system was calibrated on the day of the analysis. Electrospray source conditions were adjusted to maximise sensitivity, and the detection mode was set to detect negative (-) ions. For each run, 10, 30, or 50 µl of solution was injected with a flow rate of 0.2 mL/min and an oven temperature of 45 °C:

- Buffer A: 90 vol.% MeCN (Fisher, 99.9%): 10 vol.% LC-MS grade water (Fisher), 20 mM ammonium acetate, pH 7.4-7.5.
- Buffer B: 20 mM ammonium acetate made using LC-MS grade water (Fisher), pH 7.4-7.5.
- Column: Agilent ZORBAX RR HILIC Plus Column, 2.1 x 100 mm, 3.5 µm
- Flow rate: 0.2 mL/min

- Gradient: [0-1 min]: 100:0 (A:B); [1-10 min] 100:0 (A:B) linearly changed to 10:90 (A:B); [10-15 min] 10:90 (A:B); [15:17] linearly changed to 100:0 (A:B) and [17-35 min] 100-0 (A:B).

Analysis of the LC-MS data was performed using OpenLab CDS software.

High-resolution LC-MS. An Agilent 6546 LC/Q-TOF was coupled to a 1290 Infinity II Bio LC (Agilent Technologies, Santa Clara, CA) with an Agilent Jet Stream electrospray ionisation source (AJS). The instrument was run in negative-ion mode with an m/z range of 100-1100 and a scan rate of 1 spectrum per second in MS1 mode. Source parameters were as follows; gas temperature, 225°C; gas flow, 9 L/min; Nebulizer, 30 psig; sheath gas temperature, 375 °C, sheath gas flow 12 L/min; capillary voltage, 3 kV; nozzle voltage 500 V and fragmentor voltage 175 V. For each run, 5 µl of sample was injected with a flow rate of 0.2 mL/min and an oven temperature of 45 °C:

- Buffer A: 90 vol.% MeCN (Fisher, 99.9%): 10 vol.% LC-MS grade water (Fisher), 20 mM ammonium acetate, pH 7.4-7.5.
- Buffer B: 20 mM ammonium acetate prepared using LC-MS grade water, pH 7.4-7.5.
- Column: Agilent ZORBAX RR HILIC Plus Column, 2.1 x 100 mm, 3.5 µm
- Flow rate: 0.2 mL/min
- Gradient: [0-1 min]: 100:0 (A:B); [1-10 min] 100:0 (A:B) linearly changed to 10:90 (A:B); [10-15 min] 10:90 (A:B); [15:17] linearly changed to 100:0 (A:B) and [17-35 min] 100-0 (A:B).

VITAS assay for RSAD2 and its variants. We previously developed and described the VITAS assay.[2] This assay was modified by introducing the plasmid expressing CYB5R3. Briefly, *E. coli* BL21-AI competent cells were used and transformed with plasmids expressing RSAD2, LdrD, CYB5R3, or their combinations. To transform *E. coli* cells, 3 µl of a 100 ng/µl mini-prep stock of each plasmid was used. After transformation, 1% arabinose (final concentration) was added to the cells, which were then spread on an agar plate containing appropriate antibiotics. The RSAD2 plasmid (pBAD/ His C), LdrD plasmid (pET18b), and CYB5R3 plasmid (pACYC-Duet 1) are ampicillin, kanamycin, and chloramphenicol resistant, respectively. After overnight incubation at 37 °C, a single colony was picked the next day and inoculated into 2 ml of LB media containing 0.01% arabinose. pET and pACYC-Duet 1 plasmids are leaky, and thus, the expression of toxin LdrD and CYB5R3 does not require the addition of IPTG.[2] Cells were grown for an additional 5-6 hours in a shaker (200 rpm) at 37 °C, and bacterial growth was subsequently monitored by measuring OD at 600 nm in 1.5 mL plastic cuvettes. The growth of each RSAD2 construct (wild-type (WT) or variant) was plotted relative to its live control cells, i.e., cells not transformed with LdrD but transformed with WT and CYB5R3 or an RSAD2 variant and CYB5R3. Chain-termination activity of WT-SAND and its variants was determined using equation 2:

$$(\%)\ \boldsymbol{of\ live\ cells\ relative\ to\ control} = \left(\frac{(\boldsymbol{OD_{600nm}})_{\boldsymbol{assay}}\times \boldsymbol{100}}{(\boldsymbol{OD_{600nm}})_{\boldsymbol{live\ control}}}\right) \div \left(\frac{\boldsymbol{Vol.\ intensity_{Enzyme}}}{\boldsymbol{Vol.\ intensity_{WT-RSAD2}}}\right) \qquad (2)$$

In which

- OD_{600nm} (assay) is the optical density of cells transformed with RSAD2 (wild-type (WT), V1, or V2), CYB5R3, and LdrD.
- OD_{600nm} (live control) is the optical density of cells transformed with WT-RSAD2 and CYB5R3.
- Vol. intensity (Enzyme) is the vol. intensity of western blot bands for the expression of WT-RSAD2, RSAD2-V1, or RSAD2-V2 corrected for the Vol. intensity of their GAPDH loading control.
- Vol. intensity (WT-RSAD2) is the vol. intensity of western blot bands for the WT-RSAD2 corrected for the Vol. intensity of its GAPDH loading control.

VITAS assay for iCAREs. Initially, *E. coli* BL21-AI competent cells were transformed with plasmids expressing an iCARE, LdrD, and empty pACYC-Duet 1, or RSAD2, CYB5R3, and LdrD (positive control), or CYB5R3 and LdrD (dead control). For the transformation of *E. coli* cells, 3 µl of 8-100 ng/µl stock mini-prep of each plasmid was used. Subsequent steps were carried out exactly as

explained above for RSAD2. Chain-termination activity of iCAREs were determined using equation 3.

$$\boldsymbol{Chain-termination\ acitivity\ relative\ to\ RSAD2} = \left(\frac{(OD_{600nm})_{assay}}{(OD_{600nm})_{\ CT-control}}\right) \div \left(\frac{Vol.\ intensity_{Enzyme}}{Vol.\ intensity_{WT-RSAD2}}\right) \quad (3)$$

In which

- OD_{600nm} (assay) is the optical density of cells transformed with an ***iCARE*** (iCARE-01or iCARE-02) and LdrD.
- OD600nm (chain-termination control or CT-control) is the optical density of cells transformed with WT-RSAD2, CYB5R3, and LdrD.
- Vol. intensity (Enzyme) is the vol. intensity of western blot bands for the expression of iCARE-01 or iCARE-02 corrected for the Vol. intensity of their GAPDH loading control.
- Vol. intensity (WT-RSAD2) is the vol. intensity of western blot bands for the WT-RSAD2 corrected for the Vol. intensity of its GAPDH loading control.

Preparation of NMR sample and NMR spectroscopy. The NMR samples used to monitor electron transfer between 15N-labelled FAD-reduced CYB5R3 and unlabelled CTP, SAM-bound $[4Fe\text{-}4S]^{2+}$ RSAD2 were prepared in a glove box, exchanging the two proteins in the same degassed buffer containing 50 mM MOPS and 300 mM NaCl, pH 7.5. All the following steps were then performed anaerobically in a glove box using a gas-tight NMR tube. FAD-oxidized ^{15}N-labelled CYB5R3, as obtained upon purification from *E. coli* cells, was first fully reduced by stepwise adding two equivalents of NADH. It was possible to follow the reduction process by recording ^{1}H-^{15}N HSQC NMR experiments at 900 MHz and 298 K, since it occurs in a slow-exchange regime on the NMR timescale. Indeed, we can easily monitor the stepwise decrease of the cross-peaks belonging to the FAD-oxidized species with the concomitant increment of the cross-peaks belonging to the FAD-reduced species along with the additions of 2 eq. of NADH. Then, 1 eq. of $[4Fe\text{-}4S]^{2+}$ RSAD2 and 2 eq. of SAM were added to FAD-reduced ^{15}N-labelled CYB5R3. No chemical shift changes were monitored in the ^{1}H-^{15}N HSQC NMR spectrum of the last mixture, indicating no redox change of the FAD-reduced ^{15}N-labelled CYB5R3 species. Then, 2 eq. of CTP were added to this mixture and a ^{1}H-^{15}N HSQC NMR experiment was acquired, showing chemical shift changes and thus monitoring a redox change of the FAD-reduced ^{15}N-labelled CYB5R3 species.

Paramagnetic 1D ^{1}H NMR spectroscopy was applied to follow hyperfine-shifted signals caused by the presence of the [4Fe-4S] cluster in RSAD2. The 1D ^{1}H paramagnetic NMR spectra were recorded at different temperatures ranging from 288 K to 298 K on a Bruker AV400 MHz spectrometer equipped with a 5 mm ^{1}H selective high-power probe without gradients. Experiments were performed with a WEFT pulse sequence, with acquisition and recycle delays of 30 and 10 ms, respectively, and a dwell time of 3 µs. Squared cosine and exponential multiplications were applied prior to the Fourier transformation. Manual baseline correction was performed using polynomial functions. Several mixtures containing unlabelled $[4Fe\text{-}4S]^{2+}$ RSAD2 and unlabelled FAD-oxidized CYB5R3 were prepared in glove-box in degassed 50 mM MOPS, 300 mM NaCl, pH 7.5. In all these mixtures, 2 eq. of SAM was first added to $[4Fe\text{-}4S]^{2+}$ RSAD2 to obtain SAM-bound $[4Fe\text{-}4S]^{2+}$ RSAD2. Then, the latter species was mixed with CTP (2 eq. or 0.4 eq.), FAD-oxidized CYB5R3 (0.5 eq. or catalytic amounts), and NADH (5 eq. or 10 eq.) following different orders of additions of these species, thus generating several mixtures, which were all investigated by paramagnetic 1D ^{1}H NMR spectroscopy. $[4Fe\text{-}4S]^{2+}$ RSAD2 was also mixed with 2 eq. or 50 eq. of Met and 5'-dAH and 1D ^{1}H NMR spectra were acquired. The addition of two equivalents of Met to $[4Fe\text{-}4S]^{2+}$ RSAD2 does not modify the paramagnetic NMR signals of the cysteine ligands. Similarly, the addition of two equivalents of 5'-dAH does not affect the NMR spectrum. This indicates that Met and 5'-dAH do not interact in the catalytic pocket. The addition of fifty equivalents of Met induces significant changes in the NMR spectrum. Two very broad new signals at 18.5 and 24.5 ppm appeared, while the signals of $[4Fe\text{-}4S]^{2+}$ RSAD2 at 16.5 and 11.5 ppm remained unchanged in chemical shift but less intense, and the other signal at 13.8 ppm disappeared. The signals at 12.0 ppm likely shift to 11.2 ppm. These results indicate that Met is bound to the cluster of RSAD2 and that an equilibrium between Met-cluster bound and Met-cluster unbound species is likely present in the solution. The following addition of fifty equivalents of 5'-dAH results in a dramatic change of the signals that become much

sharper than when only Met was present in the solution, indicating that when both reaction products are in large excess, they remain stably bound to the active site. No signal at 11.4 ppm, typical of free $[4Fe\text{-}4S]^{2+}$ RSAD2, is indeed present.

The solution NMR experiments for backbone resonance assignment were performed on a ^{13}C,^{15}N-labelled FAD-oxidized CYB5R3 sample, in a buffer solution containing 50 mM MOPS, 300 mM NaCl, pH 7.5. HNCA, HN(CO)CA, HN(CA)CO, HNCO, HNCACB, CBCA(CO)NH 3D triple resonance experiments were acquired at 900 and 950 MHz spectrometers at 309 K. 3D NMR spectra were processed using the standard Bruker software (Topspin 4.4) and analysed with CARA program.[6] Secondary structure analysis was performed by TALOS-N.[7] Backbone HN resonance assignment obtained at 309 K was transferred at 298 K using a series of ^1H-^{15}N HSQC NMR experiments recorded at incremental temperatures between 309 K and 298 K. The observed chemical shift changes, observed comparing ^1H-^{15}N HSQC spectra shown in Extended Data Fig. 3b were reported as backbone-weighted average chemical shift differences, that is, $\Delta\delta_{avg}(HN) = (((\Delta H)^2 + (\Delta N/5)^2)/2)^{1/2}$, where ΔH and ΔN are chemical shift differences for backbone amide ^1H and ^{15}N nuclei, respectively. The estimate of the chemical shift threshold value to define meaningful chemical shift differences was obtained by averaging $\Delta\delta_{avg}(HN)$ values plus one standard deviation (1σ), following the standard procedure used in NMR protein-protein interaction studies.[8]

Computational methods

Structure processing: The FASTA sequences of the 3D structures of proteins (CYB5R and RSAD2) were employed to obtain AlphaFold predictions. The protein prediction was obtained by running AlphaFold v2.0.1.[9]The structures were then processed with the AlphaFill web-server interface (https://alphafill.eu)[10] to insert missing molecules that are coordinated to the protein. These include flavin adenine dinucleotide (FAD), the [4Fe4S]-cofactor, S-adenosyl methionine (SAM), haem c cofactor, cytosine 5'-triphosphate (CTP), and ions, employing experimentally determined structures. The donor crystal structures were selected based on the highest sequence identities, which ranged from 60% to 70%. The geometry of the transplanted cofactors was optimised through the AlphaFold web server, yielding a transplant clash score (TCS) below 0.50, which is indicative of high confidence. The resulting holo-protein structural models underwent thermal equilibration via molecular dynamics (MD) simulations, enabling a detailed assessment of the conformational stability and dynamic behaviour of the bound cofactors (*vide infra*).

Cofactor preparation. Several of the cofactors present within the protein, including CTP, FAD and SAM, have already been parametrised and incorporated into the CHARMM 36 FF. However, two non-standard cofactors, haem c and [4Fe-4S], required appropriate force field parameters for non-bonded interactions, as these were not available in the force field and had to be derived from quantum-mechanical data. Accordingly, they were sourced from the scientific literature. In the case of the heme, the partial charges were taken from the work of Authenrieth *et al.*[11] While the atomic partial charge for the [4Fe-4S] cofactor, which is located within the core of the RSAD2 structure, was obtained from work published by Smith et al.[12] To address the absence of accurate force field parameters for bonded interactions, the FeS cluster was treated as a nearly rigid body by applying harmonic constraints to its internal coordinates: bonds and angles with a force constant of 100 kcal/mol, and dihedral angles with a barrier of 1 kcal/mol. Since this study focuses on the state preceding electron transfer, all cofactors were modelled in their oxidised forms: [4Fe–4S] with a total charge of +2, FAD as neutral, and haem c with a charge of +3.

Modelling protein-protein complexes: Protein-protein complex docking was performed using the High Ambiguity-Driven DOCKing (HADDOCK) 2.4 web server (haddock.science.uu.nl/services/HADDOCK), a tool that provides both a structural model and an estimate of the binding affinity between the CYB5R and RSAD2 proteins.[13] The input for this flexible docking process consisted of the previously generated Alphafold-modelled protomers, as previously described. All other variables were maintained at their default values. A clustering analysis was conducted based on root-mean-square deviation (RMSD) and number of contacts, with cut-off values set at 7.5Å and 0.75, respectively. The most favourable docked conformers were identified within each of the 10 clusters recommended by HADDOCK, based on an evaluation of the

HADDOCK score. The structure exhibiting the greatest binding affinity was selected for subsequent MD simulations.

Molecular dynamics simulations. We performed molecular dynamics simulations to investigate the thermal stability and dynamics of protein-protein complexes, starting from docking structures generated with HADDOCK. All atomic MD simulations were performed with NAMD v2.14 package[14] using the CHARMM36[15,16] force field for describing the protein and the aforementioned parameters for the cofactors. The protein-protein complex was embedded in a simulation box with initial dimensions of 168 × 137 × 90 $Å^3$, with periodic boundary conditions applied using the PSFGEN VMD plugin.[17] The system was solvated with the TIP3P[18] water model and 150 mM NaCl ions to ensure system neutrality. Protonation states were determined using pKa calculations performed with the Karlsberg2+[19] method at a pH of 7.0.

Based on these results, His-261, His-201, and His-290 in the CYB5R proteins were treated as deprotonated, while all histidine residues in RSAD2 were modelled in the d-tautomeric form. All other basic and acidic residues were assigned their standard protonated states at pH 7. The time integration step of 2 fs was enabled by the SHAKE algorithm, freezing all bonds containing hydrogens.[20] Short-range electrostatics and van der Waals (vdW) interactions were treated with a cut-off of 12 Å, with a smooth switching function applied between 10 and 12 Å to reduce cutoff noise, while long-range electrostatics were handled with the particle mesh Ewald (PME) summation scheme.[21,22] The temperature T = 300 K and the pressure p = 1 bar were obtained using Langevin piston dynamics.[20] Prior to the production dynamics, all models were energy minimised for 10,000 steps while constraining the positions of all heavy atoms (position restraints of 25 kcal/mol/å^2) using the conjugate gradient algorithm.

These restraints were sequentially released during the subsequent heating (to T = 300K) and water equilibration of 500 ps. The production run was performed for 50 ns under NPT conditions, applying periodic boundary conditions throughout all simulation steps. During the production run, no constraints were imposed except for those applied on the FeS cluster. To ensure reproducibility, the MD simulations for each model system were performed in triplicate.

The conformational stability of the complex was quantitatively evaluated by calculating the root-mean-square deviation (RMSD) of heavy atoms relative to the initial model structures and the root-mean-square fluctuation (RMSF) of individual protein residues over the course of the simulations. Furthermore, the structural integrity of the cofactors and their surrounding binding environments was examined by analysing coordination geometry, hydrogen-bonding interactions, and local conformational fluctuations, thereby providing additional validation of the overall stability and reliability of the modelled complex.

Analysis. The analysis was conducted using VMD (version 1.9.1), which enabled the visualisation and evaluation of the data based on the final 10 ns of the production trajectories. Furthermore, custom Python scripts were developed to calculate the RMSD, RMSF, and the distance between the donor and acceptor utilising packages such as Numpy1.20.3 and MDAnalysis2.0-0.[23] In order to estimate the binding affinity value, PRODIGY[24] was employed using the final snapshot of the trajectory. The electron transfer pathways and corresponding rates were calculated using the Pathways 1.2 plugin for VMD.[17] To estimate the electronic coupling (T_{DA}) between the cofactors, we employed the pathway model proposed by Beratran and coworkers.[25] In this model, it is assumed that the electron transfer from the donor to the acceptor can be mediated by covalent bonds, hydrogen bonds, and through-space contact. In this study, the ET rate was calculated using the approximation $k_{ET} \approx A|\varepsilon|^2$, where the A prefactor is assumed to be 10^{14} s^{-1}, following the same steps as previous work published by the group.[26] Average ET-rate values were estimated using the 1000 snapshots from the final 10 ns of the MD trajectories.

Supplementary Figures

a) CYB5R1

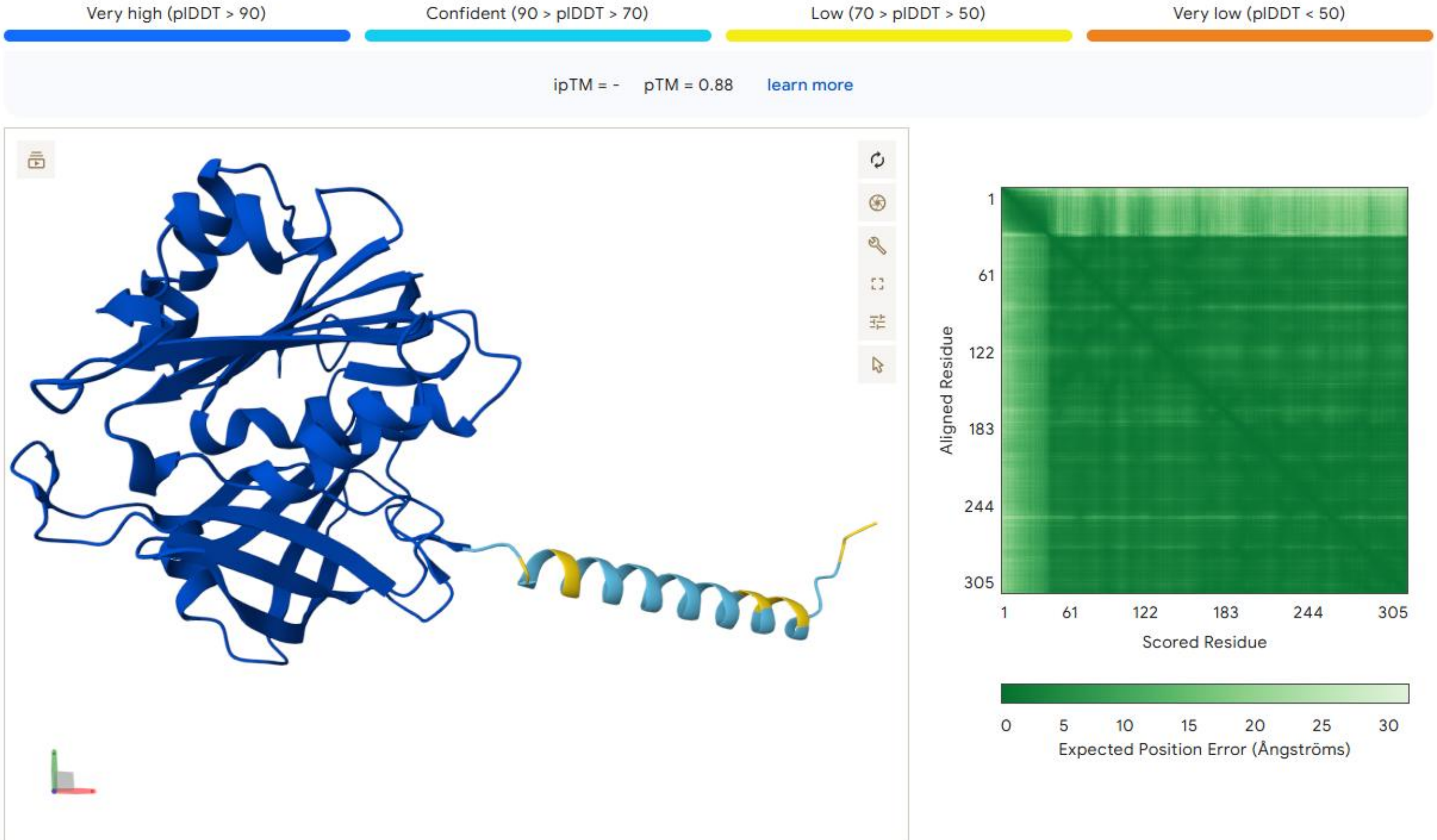

b) CYB5R2

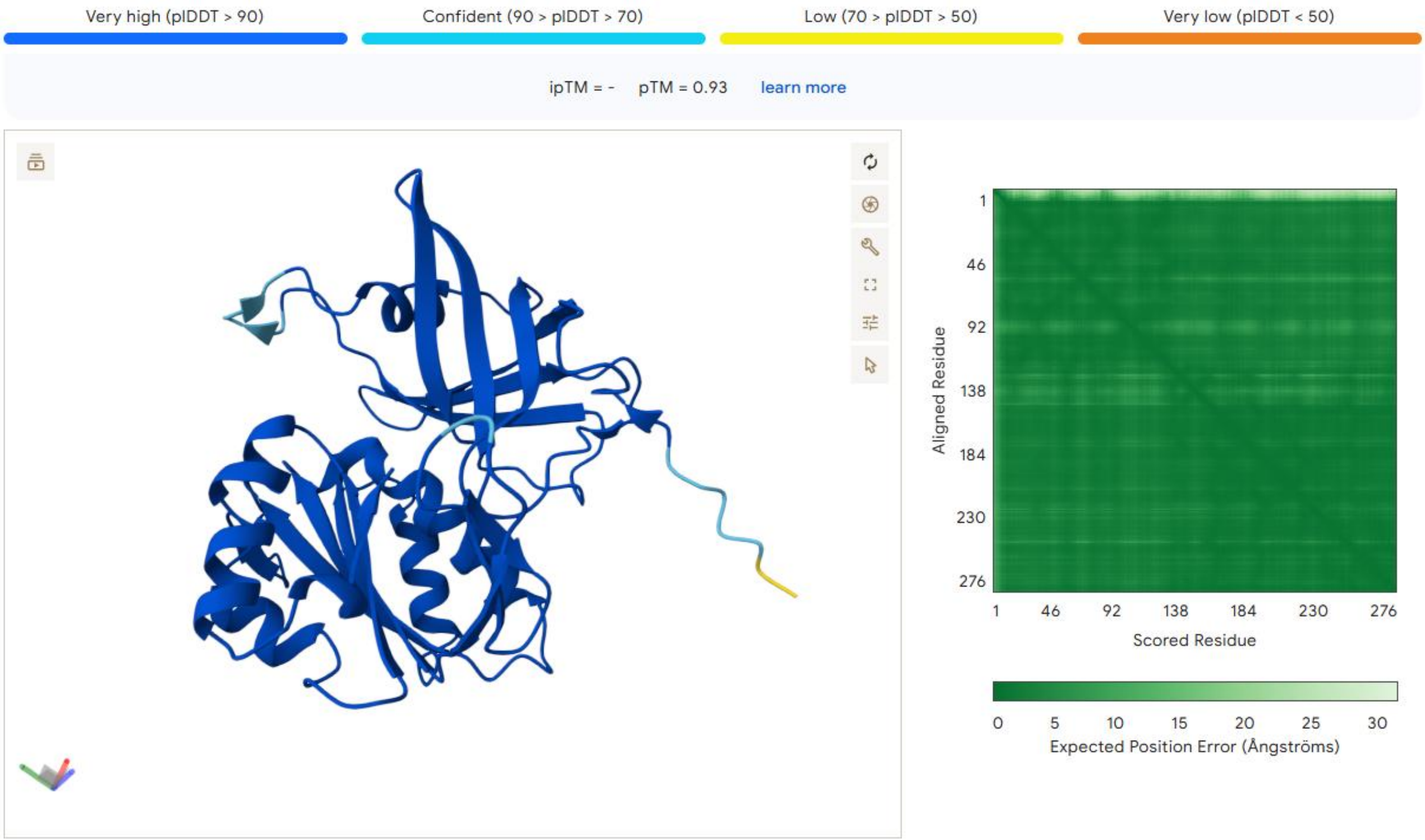

c) **CYB5R3**

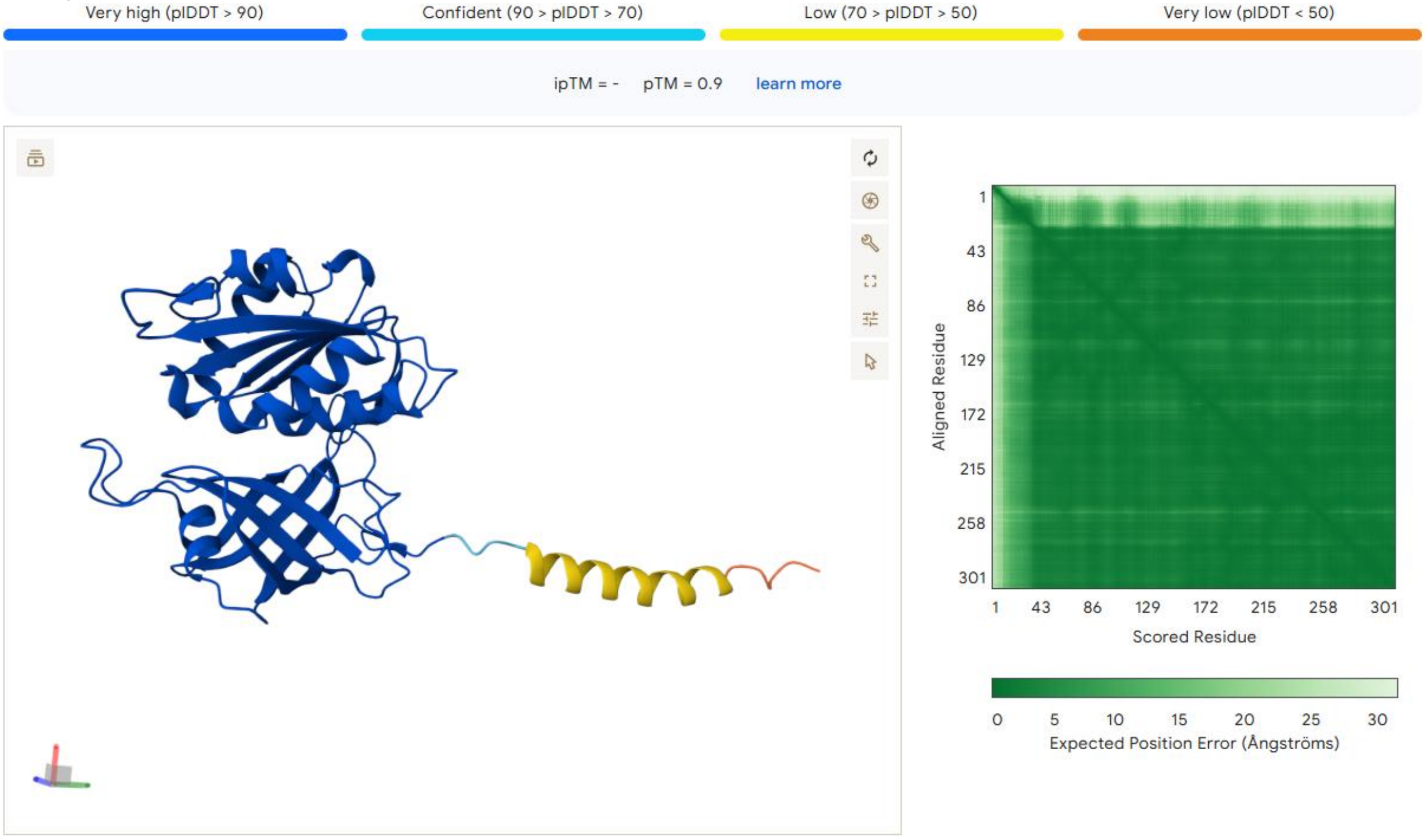

d) **CYB5R4**

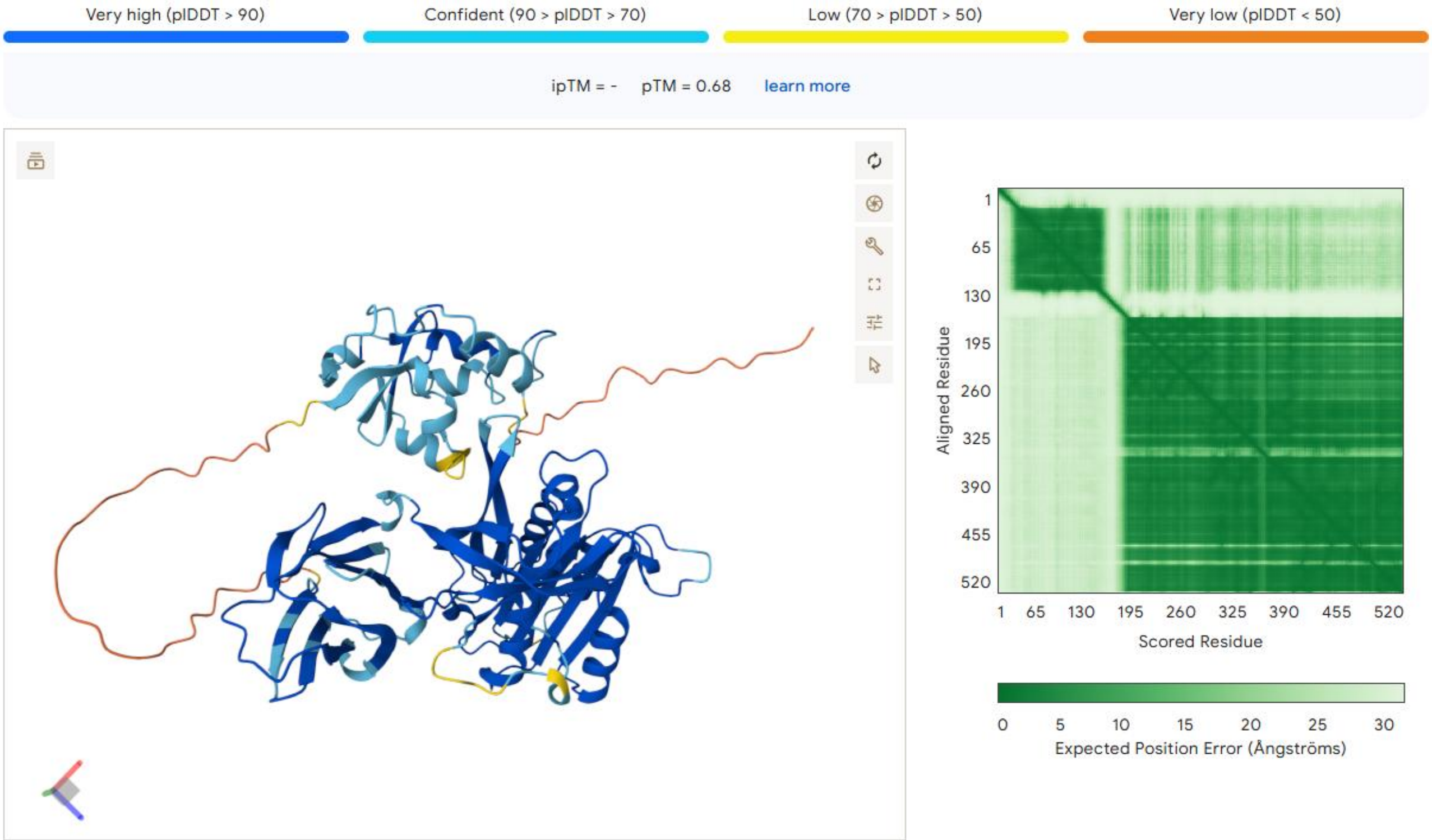

e) RSAD2

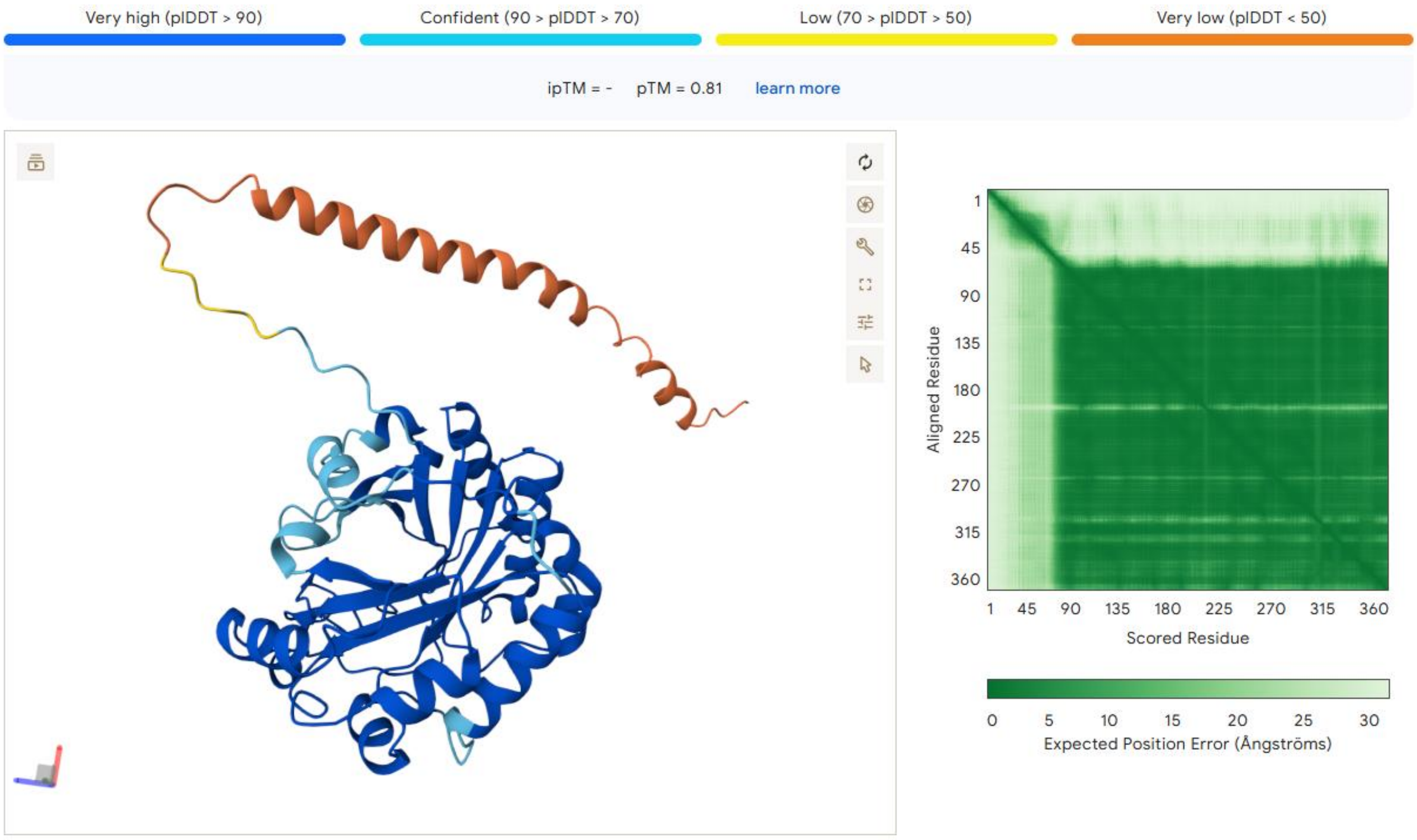

Figure S1. The results of AlphaFold-predicted models for all **(a-d)** CYB5R members and **(e)** RSAD2 (hSAND), including the pLDDT graphs. The charts show that the predictions have low or very low confidence for the N-terminal membrane-anchoring domains of RSAD2, CYB5R1, and CYB5R3, and for the flexible peptide between the Cyt-b5 and the CYB5R domain in CYB5R4.

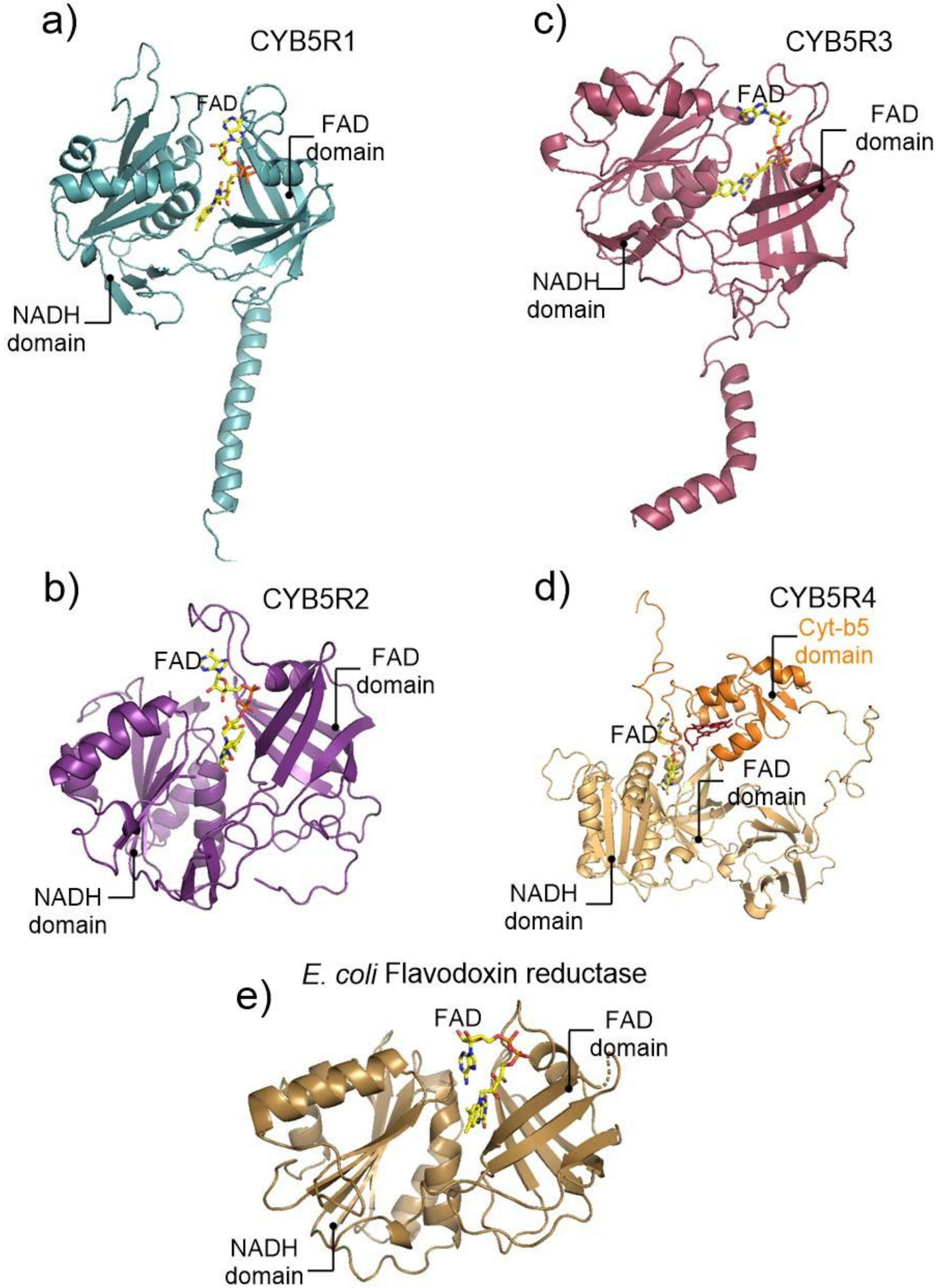

Figure S2. The predicted structures of all CYB6R members are compared to the structure of *E. coli* flavodoxin reductase. **(a)** Structure of CYB5R1, **(b)** CYB5R2, **(c)** CYB5R3, and **(d)** CYB5R4. **(e)** The X-ray crystal structure of E. coli flavodoxin reductase (PDB Code: 1FDR).

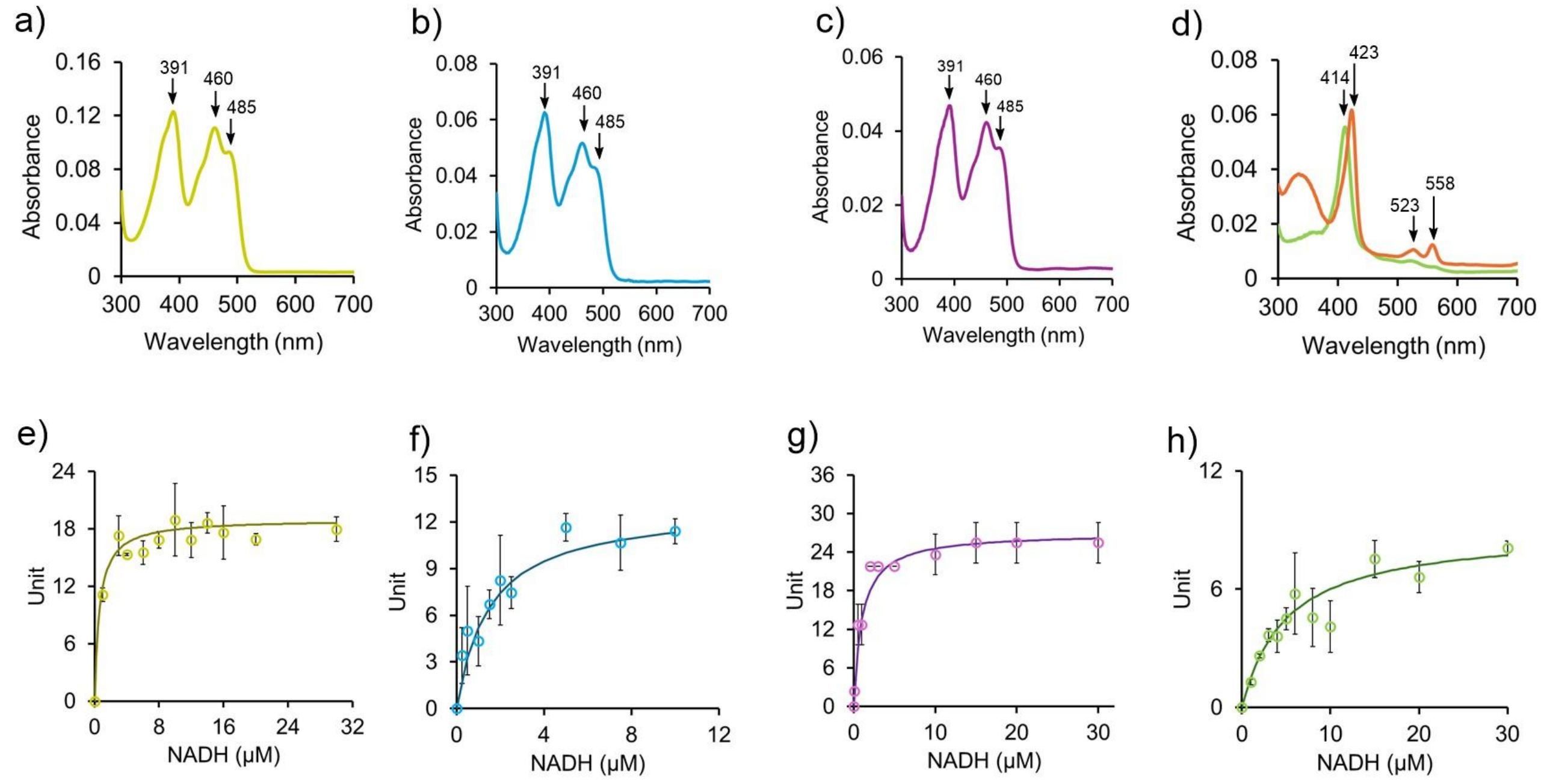

Figure S3. Characterisation of CYB5R members. UV-visible absorbance spectrum of purified **(a)** CYB5R1, **(b)** CYB5R2, **(c)** CYB5R3, and **(d)** oxidised and reduced CYB5R4. Michaelis-Menten kinetics of **(e)** CYB5R1, **(f)** CYB5R2, **(g)** CYB5R3 and **(h)** CYB5R4. Concentrations of (a) CYB5R1, (b) CYB5R2, (c) CYB5R3 and (d) CYB5R4 were 1.1, 1.1, 1.4, and 1.1 μM. **(e-h)** The concentrations of CYB5R1 and R3 were 0.0011 μM, and (f) that of CYB5R4 was 0.0048 μM. The DCIP concentration was 5 μM. Unit activity, μmol of DCIP reduced per min per mg of the enzyme. Data are averages of three independent measurements ± standard deviation. Final buffer was 50 mM Tris at pH 7.5, containing 300 mM NaCl. All measurements were performed at room temperature, 25 °C.

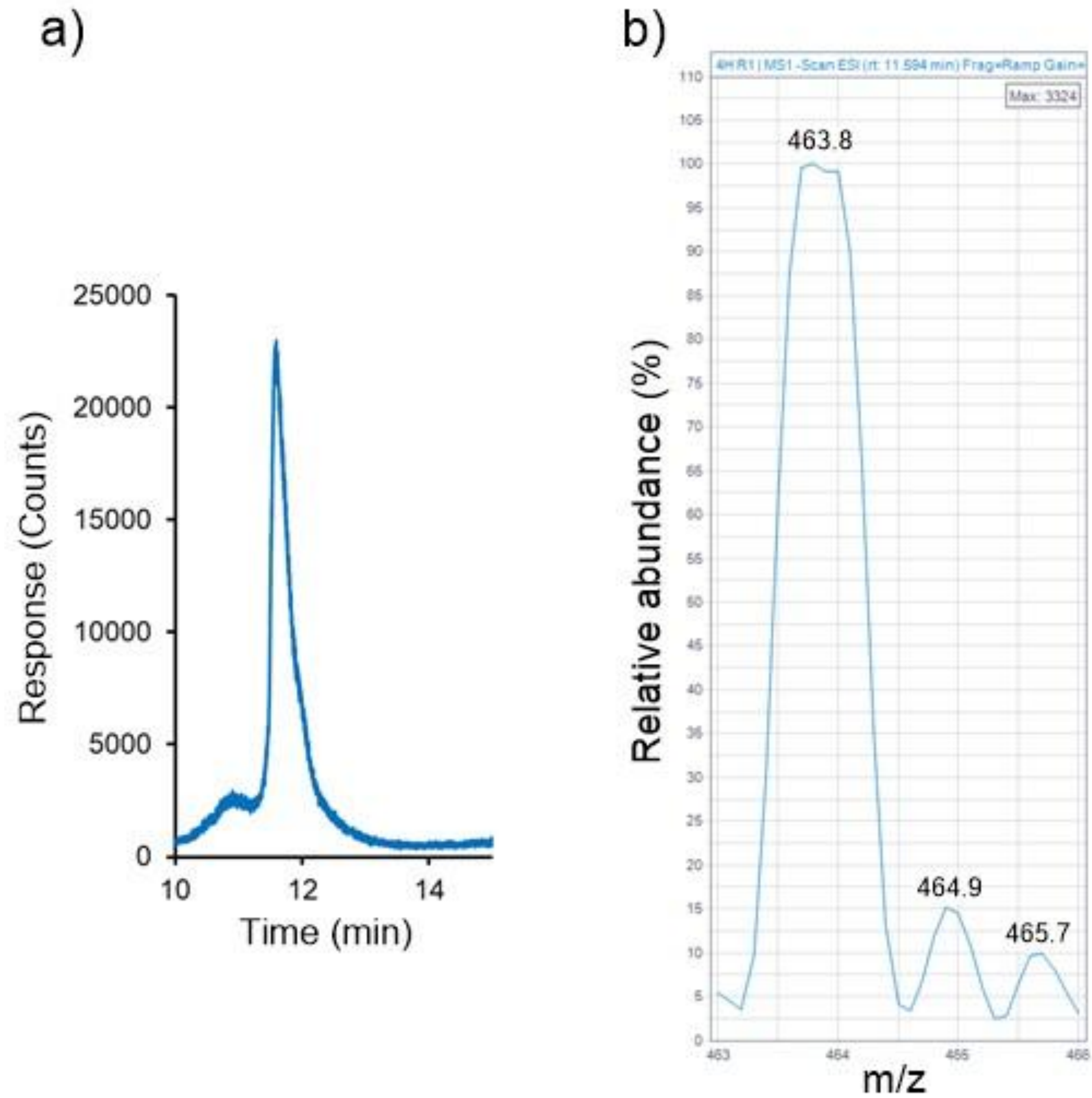

Figure S4. LC-MS analysis of ddhCTP. **(a)** Example of extracted ion chromatogram (EIC) of **(b)** ddhCTP with m/z $[M\text{-}H]^{-1}$ of 464.0± 0.1.

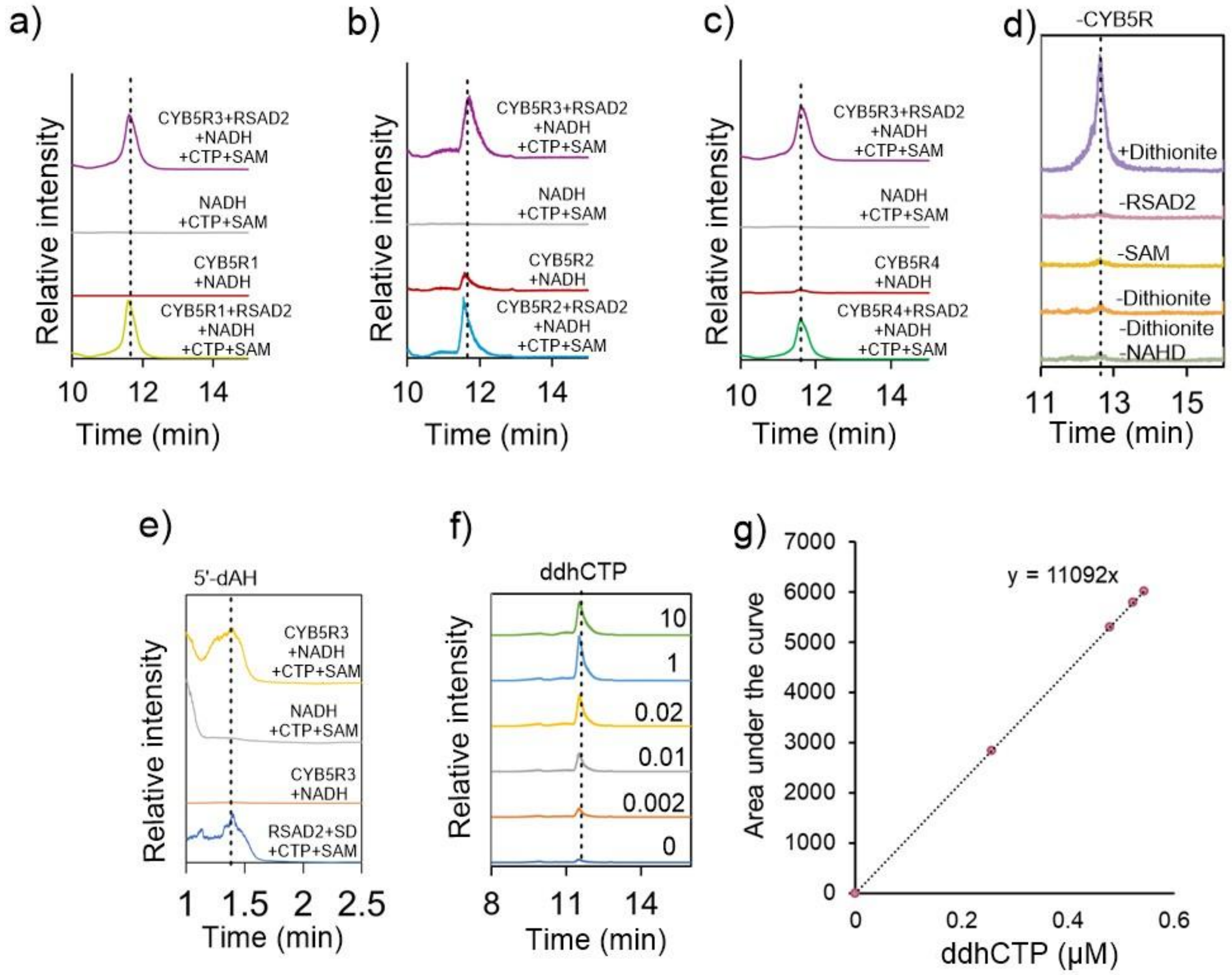

Figure S5. In vitro activity of RSAD2 with each CYB5R member. The relative intensity of the EIC for ddhCTP $[M\text{-}H]^{-1}$ with a m/z of 464.0 for **(a)** CYB5R1 (11 μM), **(b)** CYB5R2 (10 μM), and **(c)** CYB5R4 (11 μM) relative to that for CYB5R3 (11 μM). The concentrations of RSAD2, SAM, CTP, NADH, and dithionite were 0.055 mM, 11 mM, 5.5 mM, 22 mM, and 5.5 mM, respectively. **(d)** The presence of the CYB5R member is required to provide electrons from NADH to RSAD2. The relative intensity of the EIC of ddhCTP $[M\text{-}H]^{-1}$ with a m/z of 464.0 for samples lacking a CYB5R member. Besides CYB5R, the reaction component not added to each sample is highlighted next to each chromatogram. The top chromatogram (+dithionite) contained all components except CYB5R. The final concentrations of RSAD2, CTP, SAM, NADH, and dithionite were 29 μM, 1.4 mM, 3.4 mM, 11 mM, and 11 mM, respectively. **(e)** LC-MS analysis of the formation of 5′-dAH byproduct of the conversion of CTP to ddhCTP by RSAD2 in the presence of CYB5R3. Each trace shows the extracted ion chromatogram (EIC) of 5′-dA $[M+H]^{+}$ with a m/z of 252.0, as previously confirmed using high-resolution LC-MS. Concentrations were: RSAD2 (104 μM), CTP (10.4 mM), SAM (10.4 mM), CYB5R3 (12.8 μM), and NADH (20.9 mM). Experiments were repeated at least three times to confirm reproducibility. **(f)** Extracted ion chromatograms (EIC) for ddhCTP formation after the addition of different amounts of NADH. The number next to each chromatogram shows the NADH/CTP ratio. Each trace shows the ddhCTP extracted ion chromatogram $[M\text{-}H]^{-1}$ (m/z) = 464.0. The NADH concentrations were 0, 0.004, 0.02, 0.04, 2, and 20 mM; the concentration of RSAD2 was 36 μM, that of CYB5R3 was 50 μM, and those of SAM and CTP were 8 and 2 mM. **(g)** Standard curve of ddhCTP by plotting the area under the curve for the extracted ion chromatogram (EIC) of ddhCTP $[M\text{-}H]^{-1}$ with a m/z of 464.0 as a function of ddhCTP concentration. The slope of a linear fit to the data points was used to estimate ddhCTP concentration in different experiments.

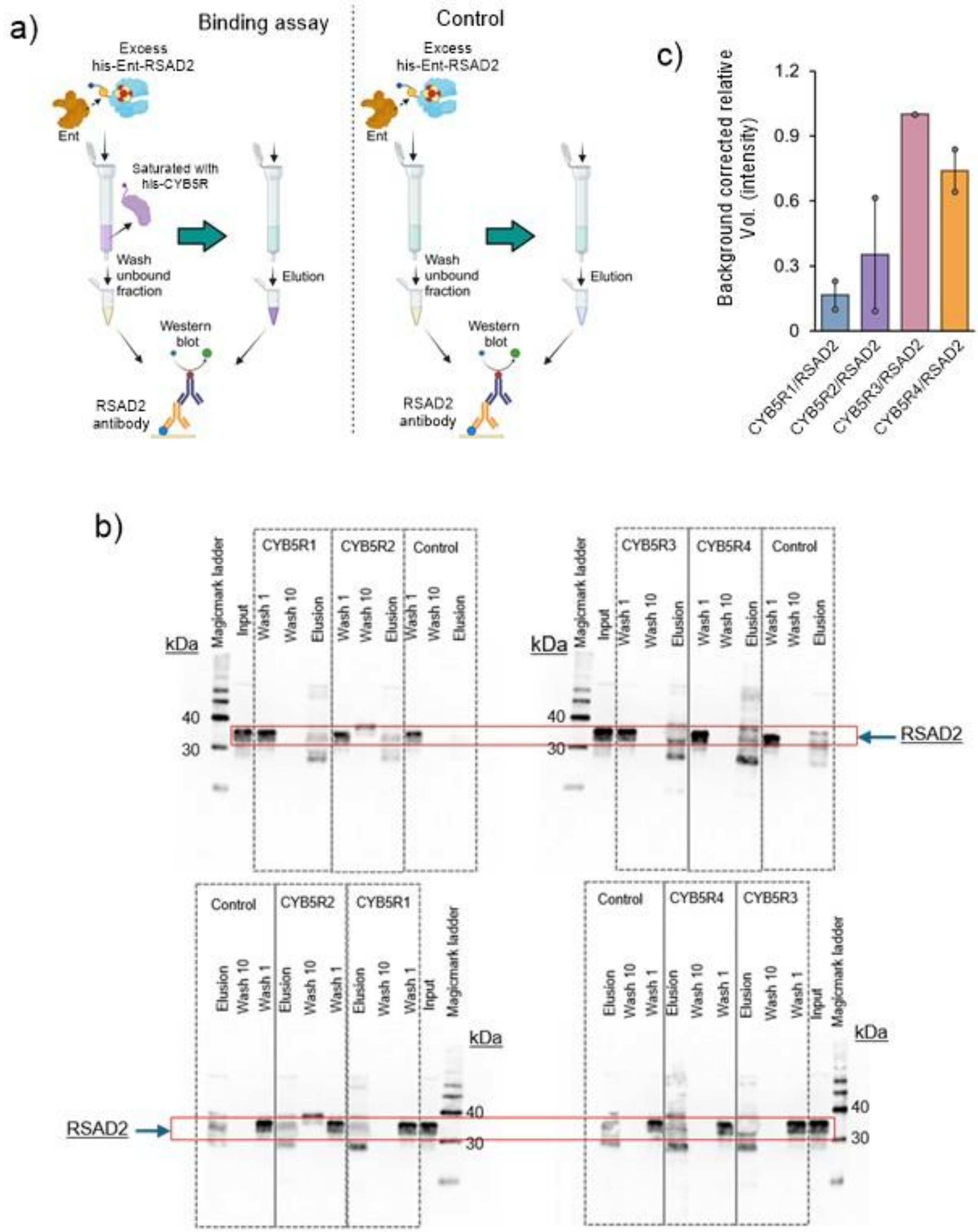

Figure S6. The interaction of CYB5R members with RSAD2 is weak and transient (non-specific) in vitro. (a) Schematic presentation of the pulldown assay to analyse the interaction of a CYB5R member with RSAD2. **(b)** Western blot images of CYB5R interaction with RSAD2. After enterokinase cleavage, an excess amount of RSAD2 treated with enterokinase to remove the his-tag was added to a Ni^{2+}-resin preloaded and saturated with one of the CYB5R members. Control is RSAD2 treated with enterokinase added to Ni^{2+}-resin in the absence of CYB5R. Input is purified

RSAD2 treated with enterokinase. Experiments were performed twice. MagicMark™ XP Western Protein Standard. 12% SDS-PAGE gels were prepared using the SureCast Gel Handcast System. The primary antibody was RSAD2 Rabbit polyclonal Antibody (Proteintech) (1:1000), and the secondary antibody was Anti-rabbit IgG HRP-linked Antibody (Cell Signalling) (1:2000). Note that there are non-specific bands with a higher and lower molecular weight than RSAD2 (circa 35-37 kDa) in the input and elution fractions. Because the molecular weight of some bands is higher than that of RSAD2, they are unlikely to result from protein degradation. They are likely due to non-specific antibody binding to additional impurities arising from other present impurities (e.g., His-tagged enterokinase, which includes a ~115 kDa heavy chain and a 22–31 kDa light chain). **(c)** Quantification of Western blot bands relative to CYB5R3. The volumetric (vol.) intensities of the bands were obtained using iBright software. Data are the average of four biologically independent expressions of each CYB5R3 member ± standard deviation.

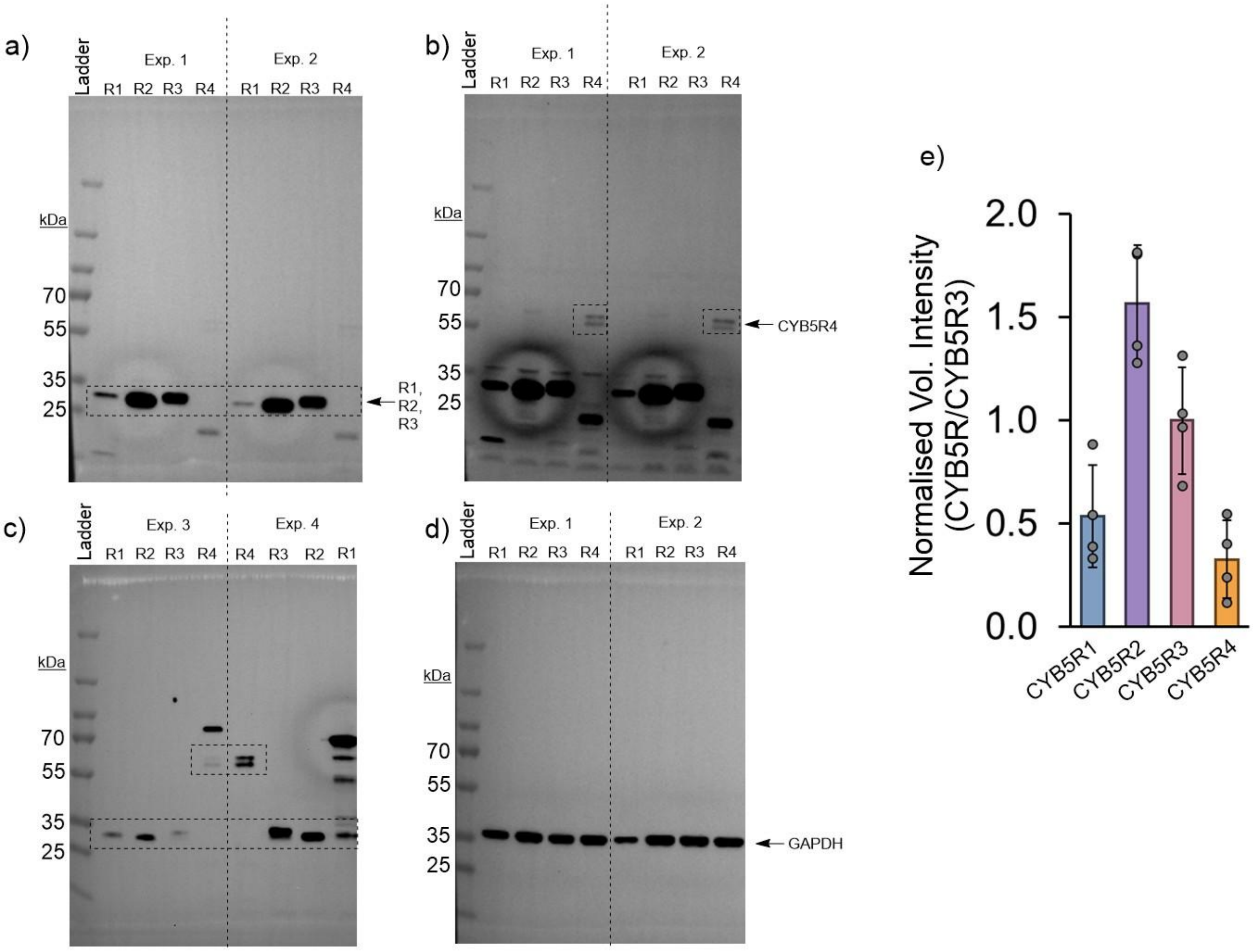

Figure S7. Western blot analysis of the expression of CYB5R members in *E. coli*. Western blot analysis of four different expressions of each CYB5R member. **(a-b)** Two expressions were imaged at different exposure times. The left image is used for analysis of CYB5R1-3, and the right image is used for analysis of CYB5R4. An HRP-conjugated His-tag antibody was used for protein detection. **(c)** Two other expressions of proteins were imaged at the same exposure time. **(d)** Western blot analysis of GAPDH as a loading control. Each protein was expressed in BL21 cells after the addition of 1 mM (final) concentration of IPTG to 5 ml of LB media. Cells were collected and washed twice with 10 mL of PBS. Cells were lysed by adding lysis buffer (method) and then sonicated. The total protein concentration was determined for each sample and normalised to the lowest concentration. For Expression (Exp.) 1, 2, 3, and 4, the normalised total protein concentrations were 1.0±0.2, 1.1±0.02, 0.40±0.02, 0.44+0.02 mg/ml, respectively. For Western blot analysis, 20 µl of protein was loaded onto each well. The antibody was a 6x-His tag monoclonal antibody-HRP conjugated (Thermo Fisher, MA1-21315-HRP). The observed molecular weight of each protein with His-tag peptide is CYB5R1, 32 kDa (lacking N-terminus), CYB5R2, 31 kDa, CYB5R3, 32 kDa (lacking N-terminus), and CYB5R4, 60 kDa. The ladder was Thermo Scientific™ PageRuler™ Plus Prestained Protein Ladder, 10 to 250 kDa. Novex 8-16% Tris-Glycine Plus WedgeWell Gel was used (Invitorogen). **(e)** Quantification of Western blot bands relative to CYB5R3. The volumetric (vol.) intensities of the bands were obtained using iBright software. Data are the average of four biologically independent expressions of each CYB5R member ± standard deviation.

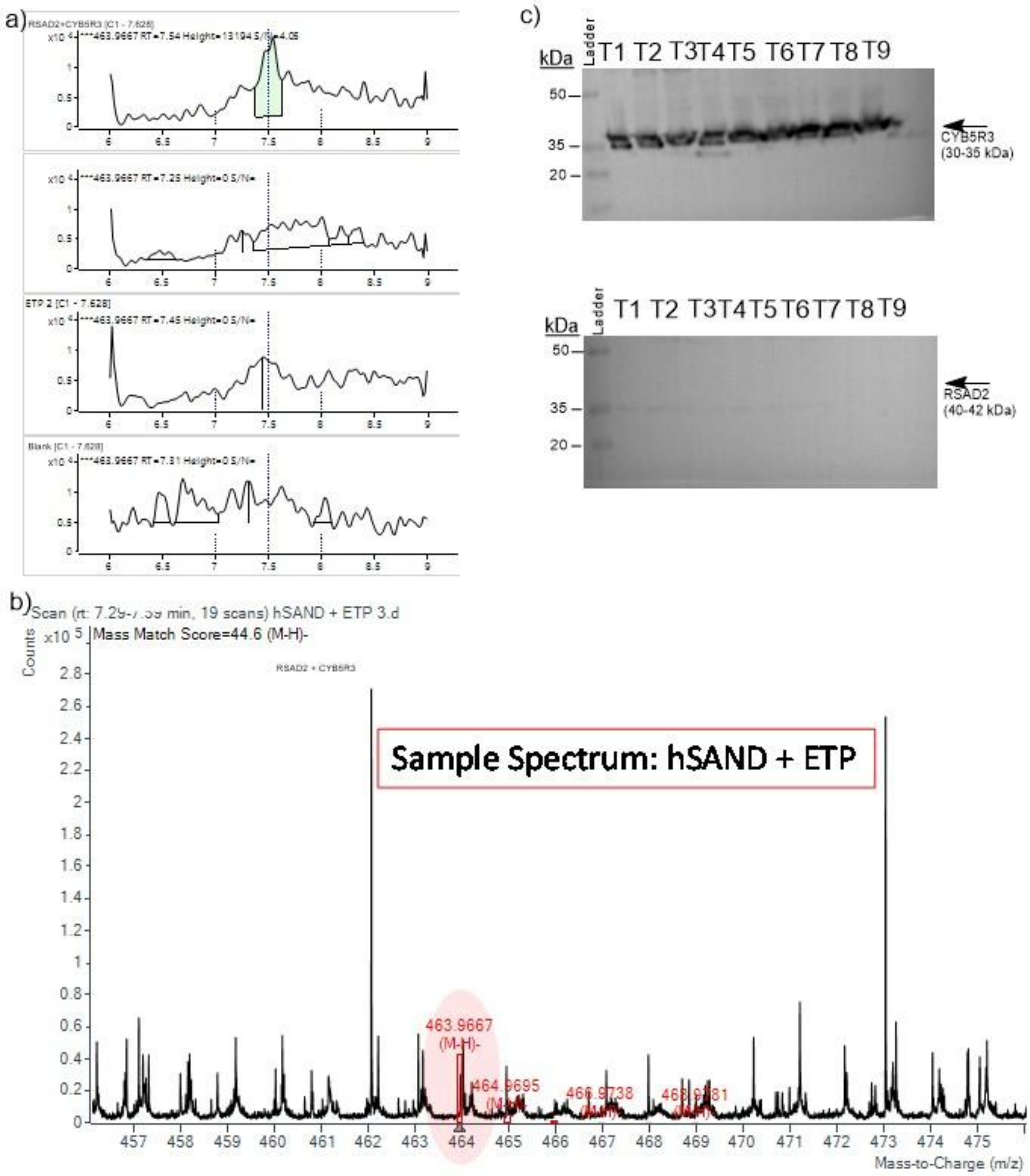

Figure S8. HEK293 cells co-expressing soluble RSAD2 (human SAND (hSAND)) and soluble CYB5R3 produce ddhCTP. **(a)** The graph shows the extracted ion chromatogram (EIC) of ddhCTP $[M-H]^{-1}$ with an accurate m/z of 463.9667 for cells (from top to bottom) expressing RSAD2 and CYB5R3, RSAD2 alone, or CYB5R3 alone. The bottom spectrum is for blank solvent. **(b)** An example of the MS spectrum of ddhCTP. **(c)** Western blot analysis of the expression of endogenous full-length CYB5R3 (with N-terminus membrane anchoring domain) (~35-37 kDa) and endogenous full-length RSAD2 (with N-terminus membrane anchoring domain) (~41 kDa) in HEK293 cells. The image shows an overlay of the Western blot and gel images to demonstrate the correct size of CYB5R3. Experiments were repeated with nine independent biological samples (T1-T9) to ensure reproducibility. The ladder was Thermo Scientific™ PageRuler™ Plus Pre-stained Protein Ladder, 10 to 250 kDa.

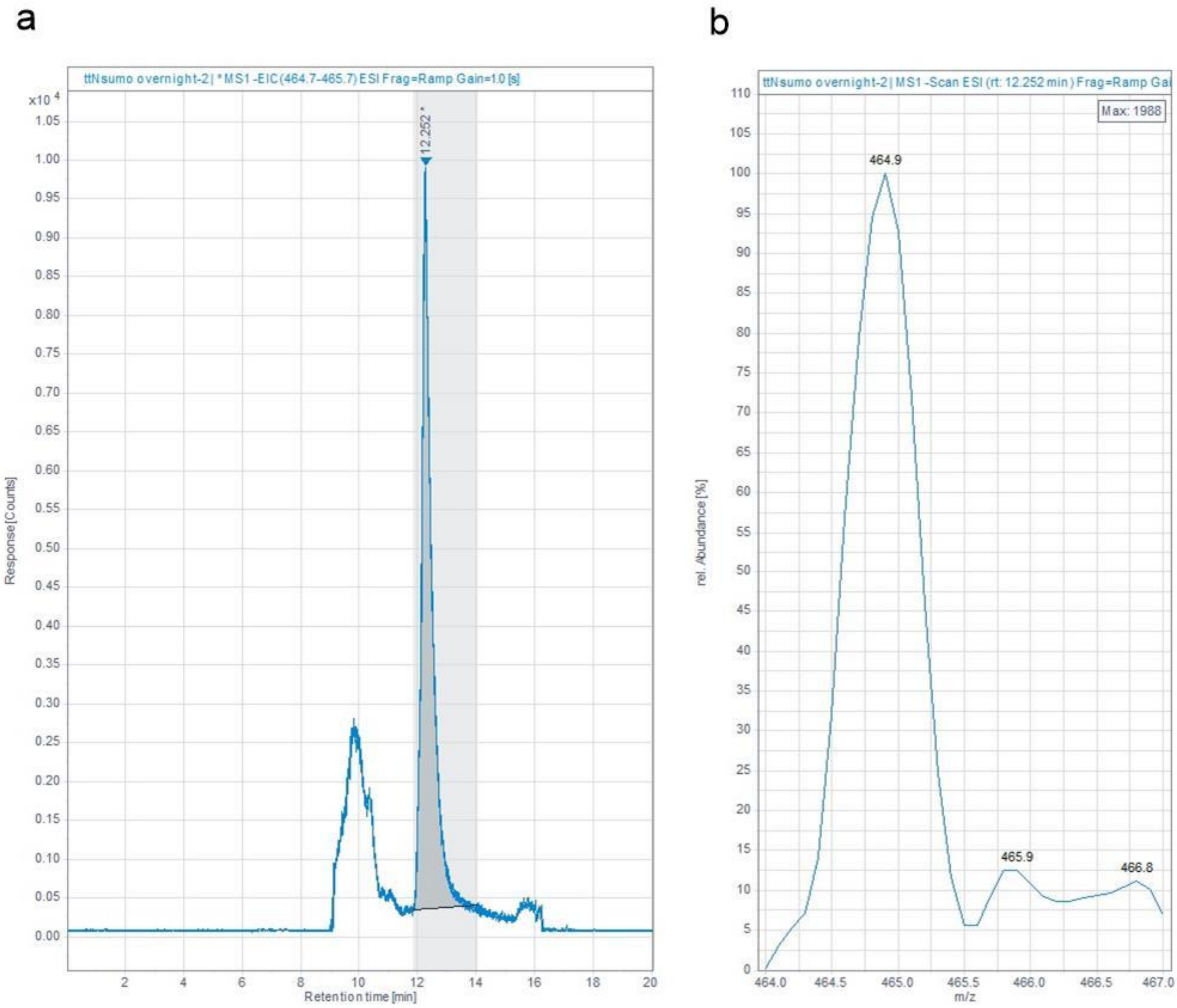

Figure S9. LC-MS analysis of ddhUTP formation. **(a)** Example of extracted ion chromatogram (EIC) of **(b)** ddhUTP with m/z $[M-H]^{-1}$ of 465.0± 0.1.

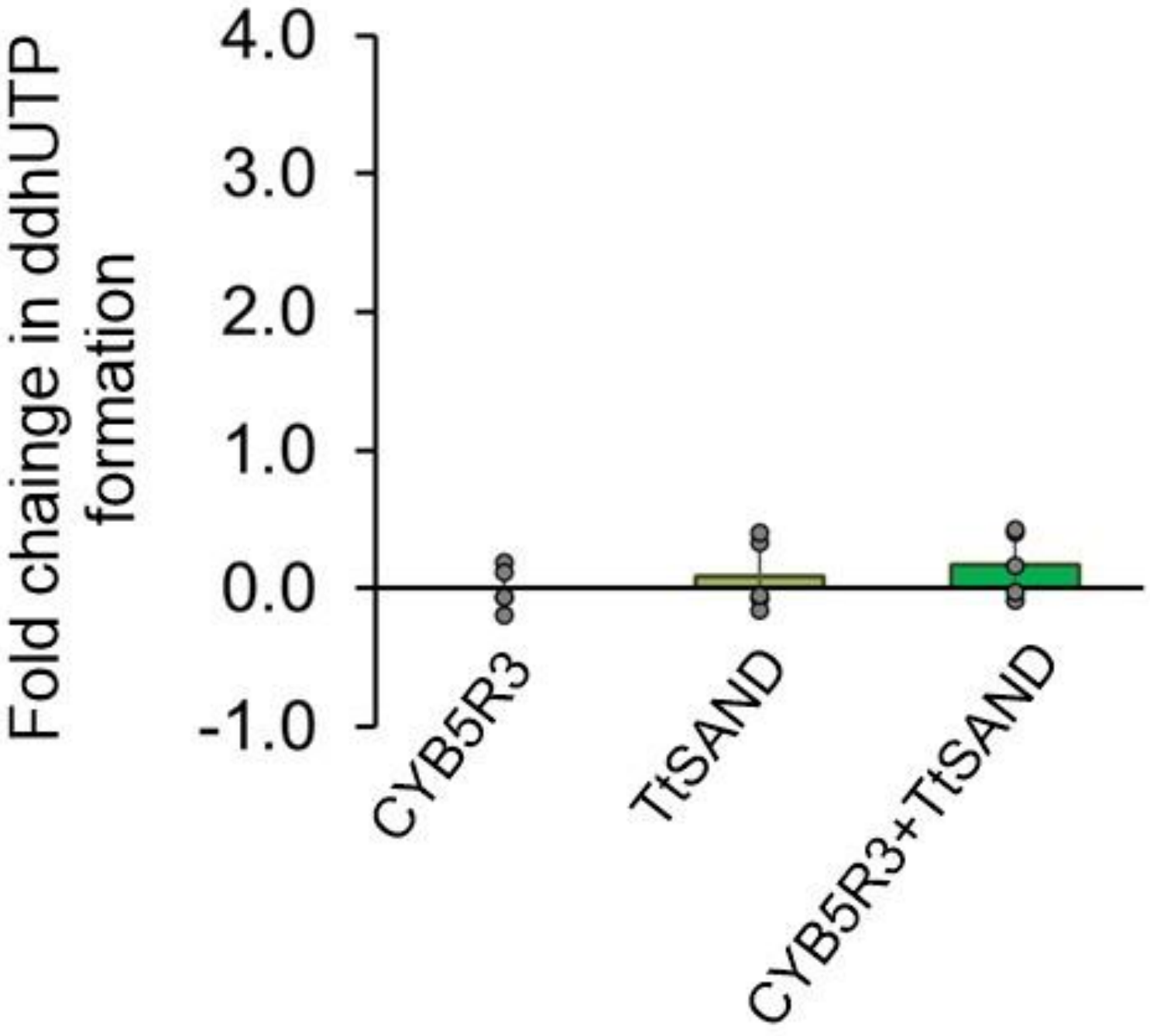

Figure S10. Targeted metabolomics to analyse formation of ddhCTP in E. Coli cells shows that co-expression of TtSAND and CYB5R3 did increase synthesis of ddhUTP in *E. coli* relative to the TtSAND-alone control. Data are the average of five measurements ± standard deviations.

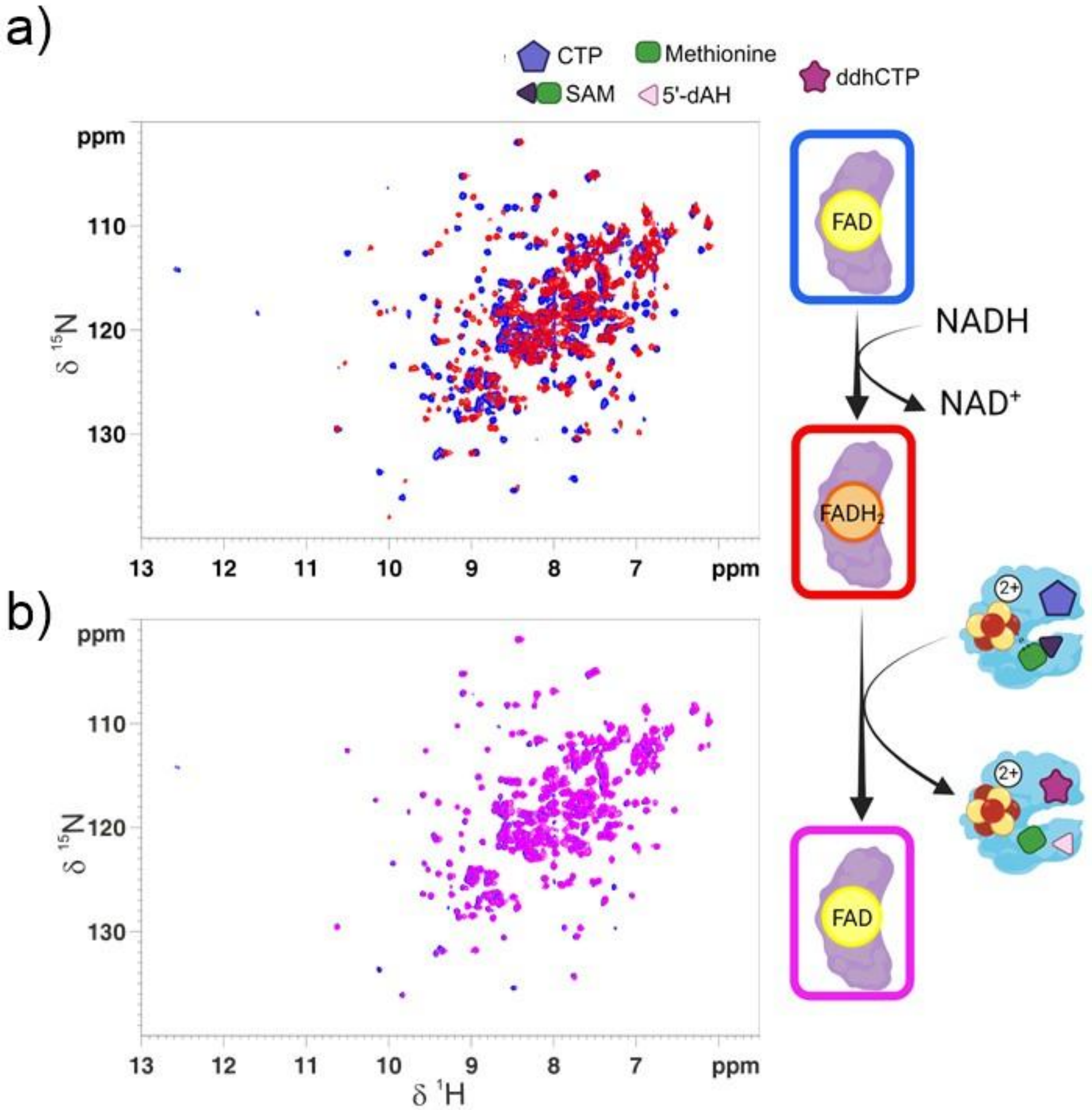

Figure S11. Monitoring the FAD oxidation state. **(a)** ^{1}H-^{15}N HSQC NMR spectra of ^{15}N-labelled CYB5R3 before and after the addition of NADH. **(b)** ^{1}H-^{15}N HSQC NMR spectra of ^{15}N-labelled CYB5R3 before the addition of NADH and after the addition of NADH, followed by the addition of unlabelled $[4Fe\text{-}4S]^{2+}$ RSAD2 (1 eq. to CYB5R3), CTP, and SAM. The same amount of NADH, SAM, and CTP was added (each 2 eq. vs. 1 eq. of the two proteins).

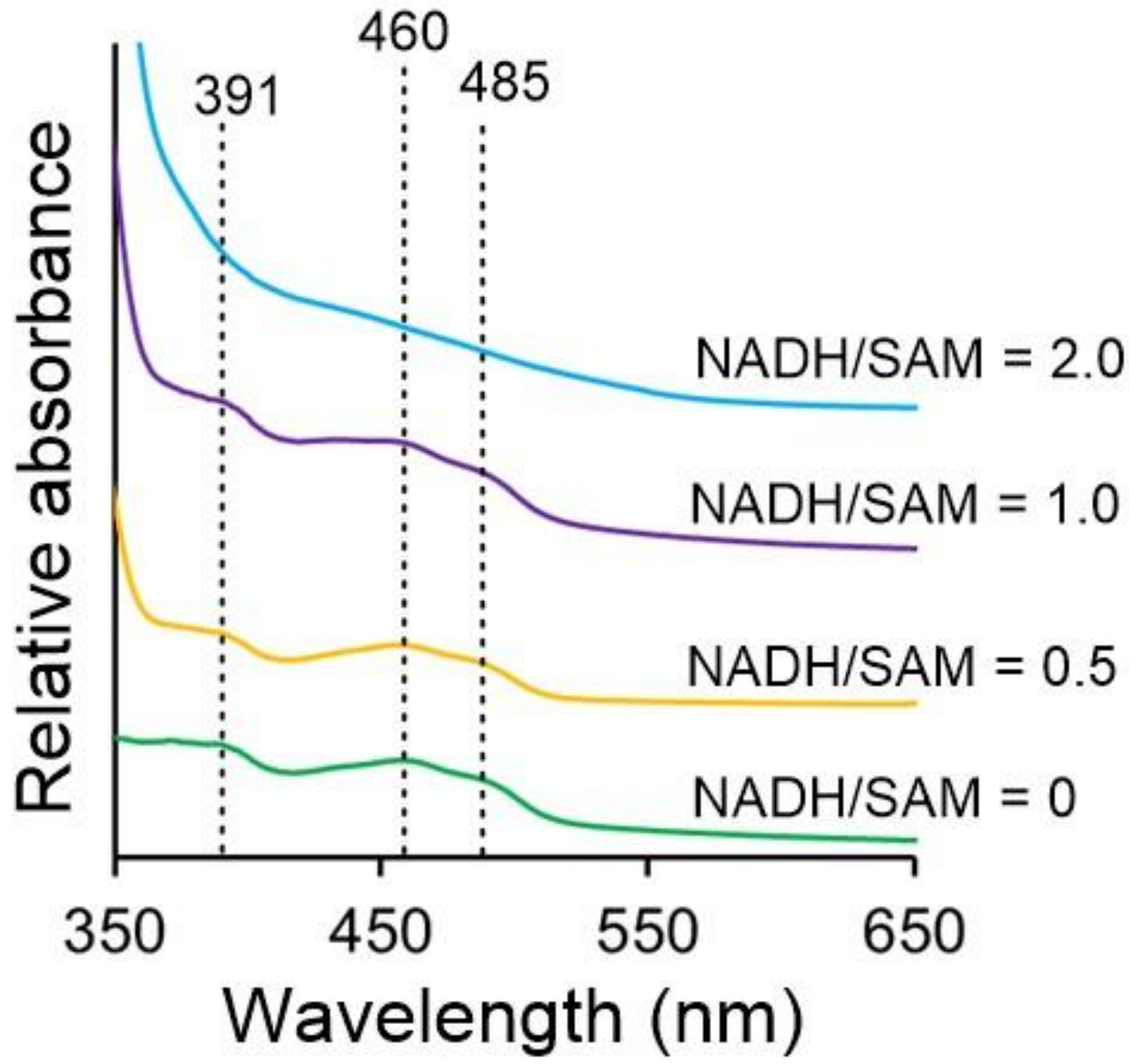

Figure S12. The presence of SAM is required for electron transfer from reduced FAD in CYB5R3 to the oxidised $[4Fe\text{-}4S]^{2+}$ cluster in RSAD2. Stacked UV-visible absorbance spectrum of CYB5R3 (1.8 µM) FAD cofactor in the presence of RSAD2 (9 µM), CTP (3.6 mM), SAM (3.6 mM), and four different NADH concentrations (0, 1.8, 3.6, and 7.2 mM). We first used UV-visible spectroscopy to follow the absorbance peak of oxidised FAD, which exhibited peaks at 391, 460, and 485 nm. We recorded UV-visible absorbance of CYB5R3 in the presence of RSAD2, CTP, SAM, and NADH. We tested how changing the NADH/SAM ratio affects the FAD absorbance peaks. The oxidised FAD peak was observed at NADH/SAM ratios below one and disappeared at ratios above one. The absence of the oxidised FAD peaks suggests that it was in the reduced $FADH_2$ state. Therefore, after SAM was fully consumed, the excess NADH reduced the FAD cofactor. The reduced $FADH_2$ could not provide electrons to the RSAD2 $[4Fe\text{-}4S]^{2+}$ cluster in the absence of SAM.

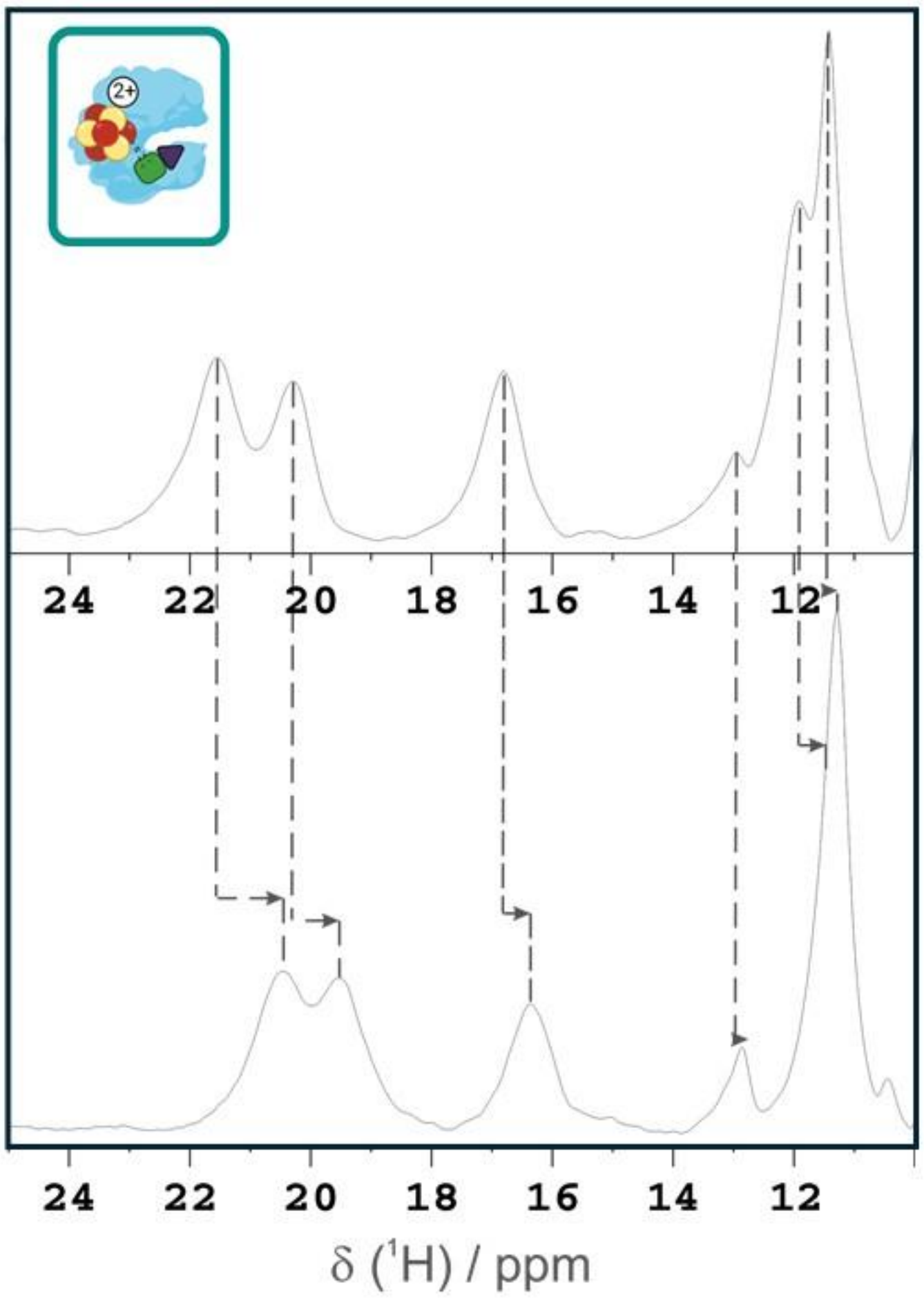

Figure S13. The anti-Curie temperature dependence of the hyperfine-shifted proton NMR signals indicates the presence of an oxidised $[4Fe\text{-}4S]^{2+}$ cluster in SAM-bound $[4Fe\text{-}4S]^{2+}$ RSAD2 (upper spectrum at 298 K, lower spectrum at 288 K). The SAM to $[4Fe\text{-}4S]^{2+}$ RSAD2 ratio was two. The concentration of $[4Fe\text{-}4S]^{2+}$ RSAD2 was 120 μM.

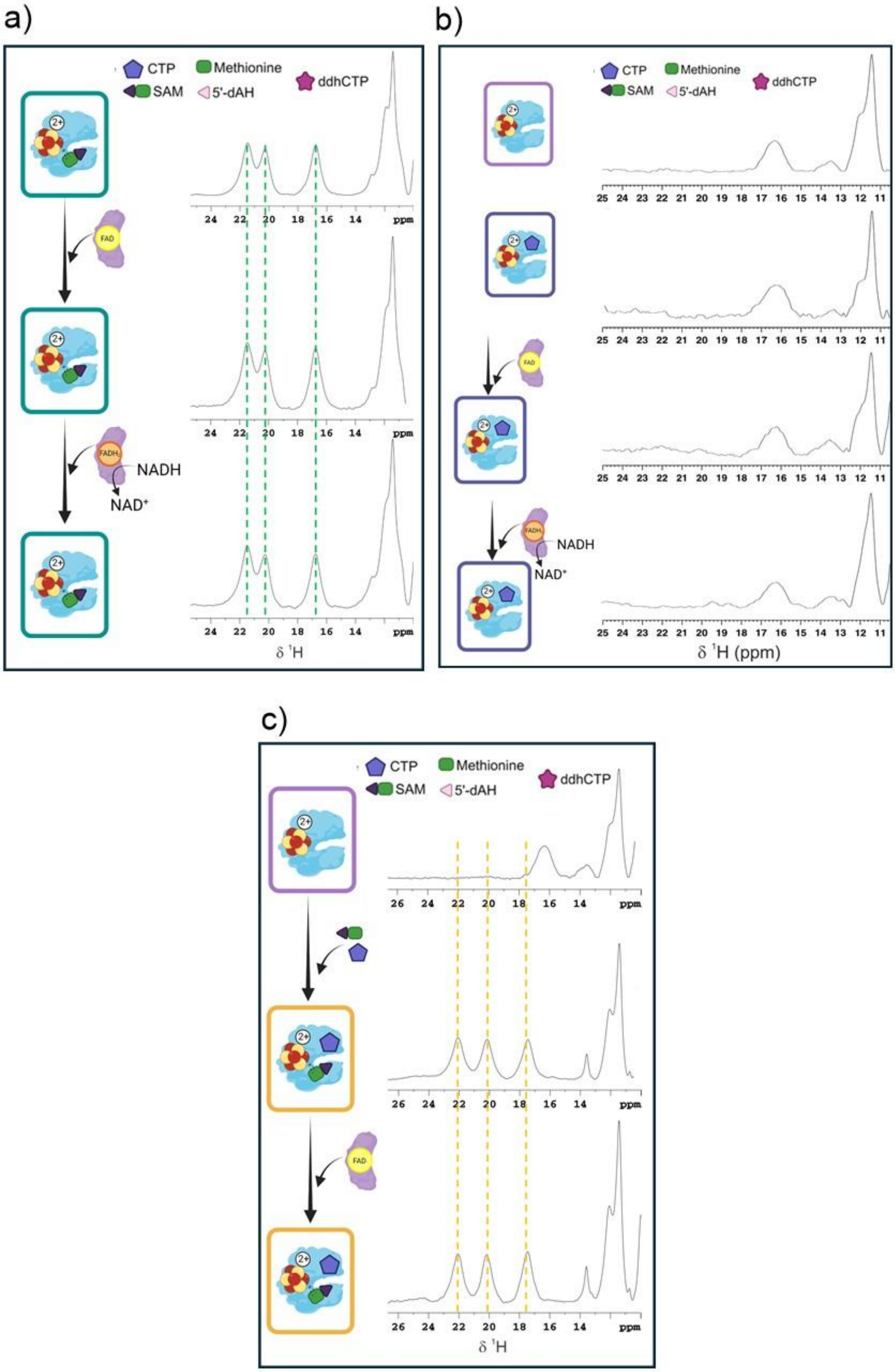
a)
b)
c)
CTP
Methionine
SAM
5'-dAH
ddhCTP
FAD
FADH2
NADH
NAD+
δ 1H
δ 1H (ppm)
ppm

Figure S14. Concomitant binding of SAM and CTP to RSAD2 is required for ET. The binding of **(a)** SAM or **(b)** CTP alone to $[4Fe\text{-}4S]^{2+}$ RSAD2 is not sufficient for ET. **(a)** The hyperfine shifts of the ligand protons of the SAM-bound $[4Fe\text{-}4S]^{2+}$ cluster do not change when CYB5R3 is added with or without NADH. **(b)** The CTP binding does not change the hyperfine shifts of the ligand protons of the cluster. The addition of CYB5R3 alone or in the presence of NADH does not change the hyperfine shifts of the ligand protons of the cluster. **(c)** The binding of SAM and CTP to $[4Fe\text{-}4S]^{2+}$ RSAD2 changes the hyperfine shifts of the ligand protons of the cluster. The addition of CYB5R3 in the absence of NADH does not change the spectra, confirming that no reaction occurred. The ratio of SAM and CTP to $[4Fe\text{-}4S]^{2+}$ RSAD2 was two. The ratios of CYB5R3 and NADH to $[4Fe\text{-}4S]^{2+}$ RSAD2 were 0.5 and 5 eq., respectively. The concentration of $[4Fe\text{-}4S]^{2+}$ RSAD2 was 120 μM.

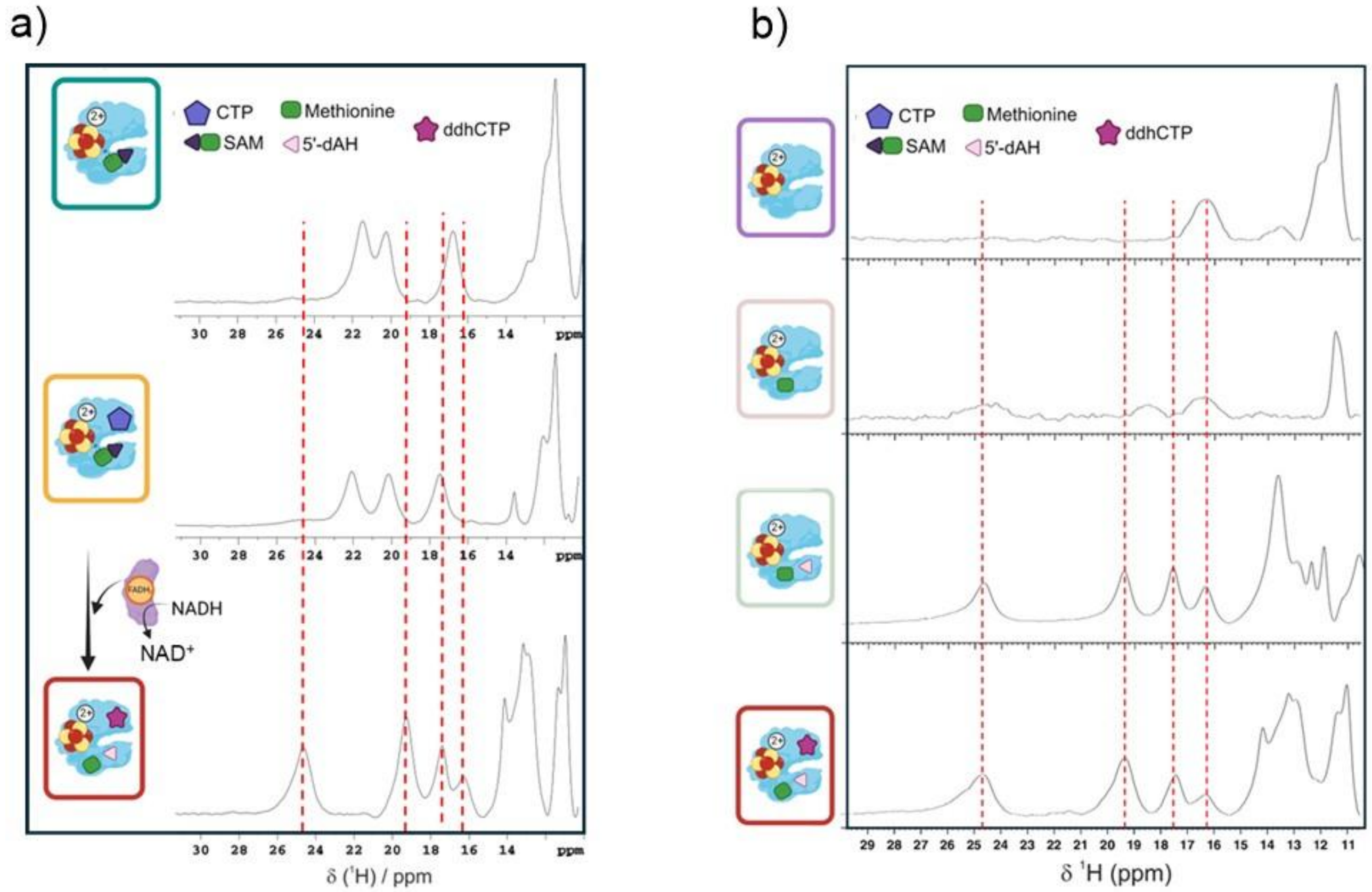

Figure S15. Electron transfer to CTP- and SAM-bound [4Fe-4S]$^{2+}$ RSAD2 generate RSAD2 bound to 5´-dAH and methionine products of catalysis. **(a)** Electron transfer happens in the presence of SAM and CTP. Paramagnetic NMR spectra of (top) [4Fe-4S]$^{2+}$ RSAD2 in the presence of SAM (2 equivalents), (middle) [4Fe-4S]$^{2+}$ RSAD2 in the presence of CTP and SAM (both at 2 equivalents), and (bottom) addition of CYB5R3 (0.5 equivalents) plus NADH (5 equivalents). The equivalent values are relative to RSAD2 concentration (120 μM). **(b)** The ET from CYB5R3 to CTP- and SAM-bound [4Fe-4S]$^{2+}$ RSAD2 generates the products of radical-SAM catalysis. From top to bottom: (i) Paramagnetic 1D ^{1}H NMR spectra of [4Fe-4S]$^{2+}$ RSAD2, and in the presence of (ii) 50 eq. of Met, (iii) 50 eq. of Met and 5'-dAH, or (iv) of the mixture containing 0.5 eq. CYB5R3, 10 eq. NADH, [4Fe-4S]$^{2+}$ RSAD2, 2 eq. SAM and 2 eq. CTP. The different signals in the region 15-10 ppm comparing spectrum (iii) vs. (iv) can be due to the presence of ddhCTP bound to the catalytic pocket (spectrum (iv)), which is absent in spectrum (iii).

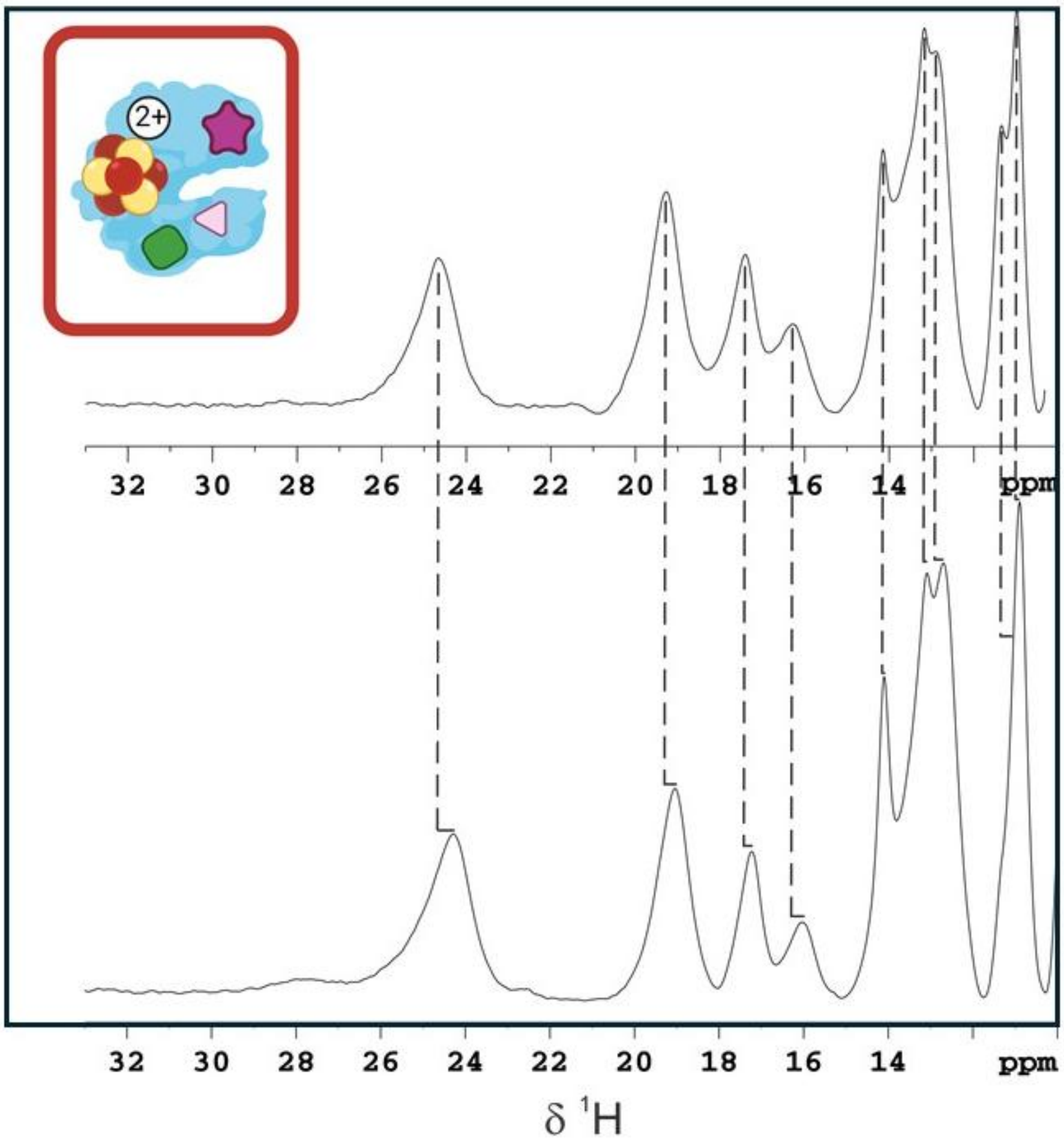

Figure S16. The anti-Curie temperature dependence of the hyperfine-shifted proton NMR signals indicates the presence of an oxidised $[4Fe\text{-}4S]^{2+}$ cluster in the reaction mixture containing $[4Fe\text{-}4S]^{2+}$ RSAD2, CYB5R3, CTP, SAM and NADH (upper spectrum at 298 K, lower spectrum at 288 K). The SAM and CTP to $[4Fe\text{-}4S]^{2+}$ RSAD2 ratio was two, the CYB5R3 to $[4Fe\text{-}4S]^{2+}$ RSAD2 ratio was 0.5 eq. or catalytic amounts of CYB5R3 and the NADH to $[4Fe\text{-}4S]^{2+}$ RSAD2 ratio was 5. The concentration of $[4Fe\text{-}4S]^{2+}$ RSAD2 was 120 mM.

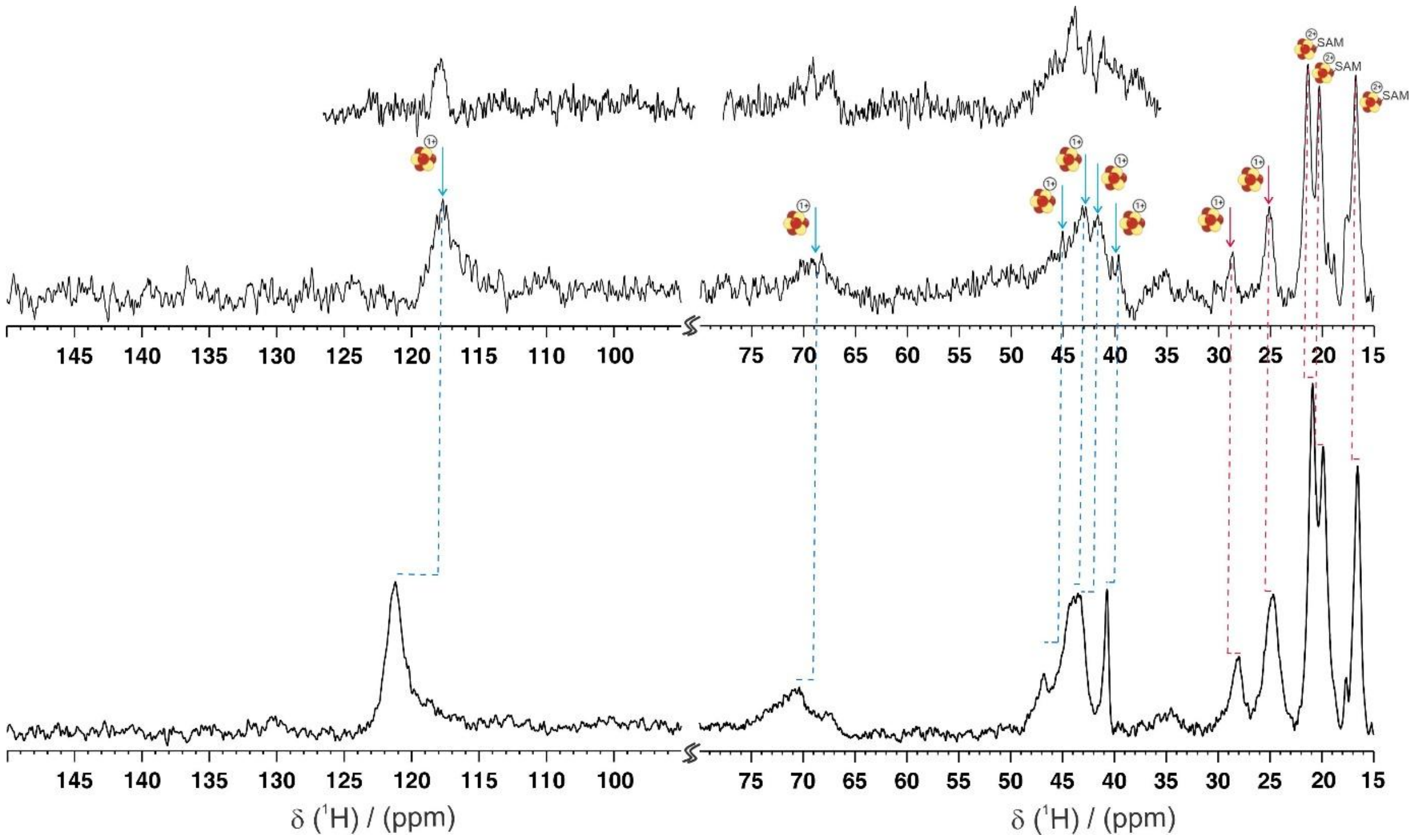

Figure S17. $[4Fe\text{-}4S]^{2+}$ cluster reduction of RSAD2 upon the electron transfer from reduced CYB5R3 to CTP- and SAM-bound $[4Fe\text{-}4S]^{2+}$ RSAD2. Paramagnetic NMR spectra of $[4Fe\text{-}4S]^{2+}$ RSAD2 in the presence of 0.4 eq. CTP, 2 eq. of SAM, 0.5 eq. of CYB5R3 and 5 eq. of NADH at 298K (top) and at 288K (bottom). The equivalent values are relative to RSAD2 concentration (120 μM). In the inset of the (top) spectrum, the down-field shifted paramagnetic NMR signals obtained by reducing the SAM-bound species of $[4Fe\text{-}4S]^{2+}$ RSAD2 with 5 mM dithionite are shown. The temperature dependence, together with the observation of the signals present in the dithionite-reduced state of the cluster of RSAD2, indicates the formation of the reduced $[4Fe\text{-}4S]^{1+}$ form of RSAD2 in the mixture containing the SAM, CTP-$[4Fe\text{-}4S]^{2+}$ RSAD2 and FAD-reduced CYB5R3. The cyan arrows identify the signals of the reduced [4Fe-4S]1+ form of RSAD2 with a Curie-type temperature dependence; the red arrows identify the signals of the reduced [4Fe-4S]1+ form of RSAD2 with anti-Curie-type temperature dependence. The signals of the SAM-bound $[4Fe\text{-}4S]^{2+}$ RSAD2 with anti-Curie-type temperature are also indicated.

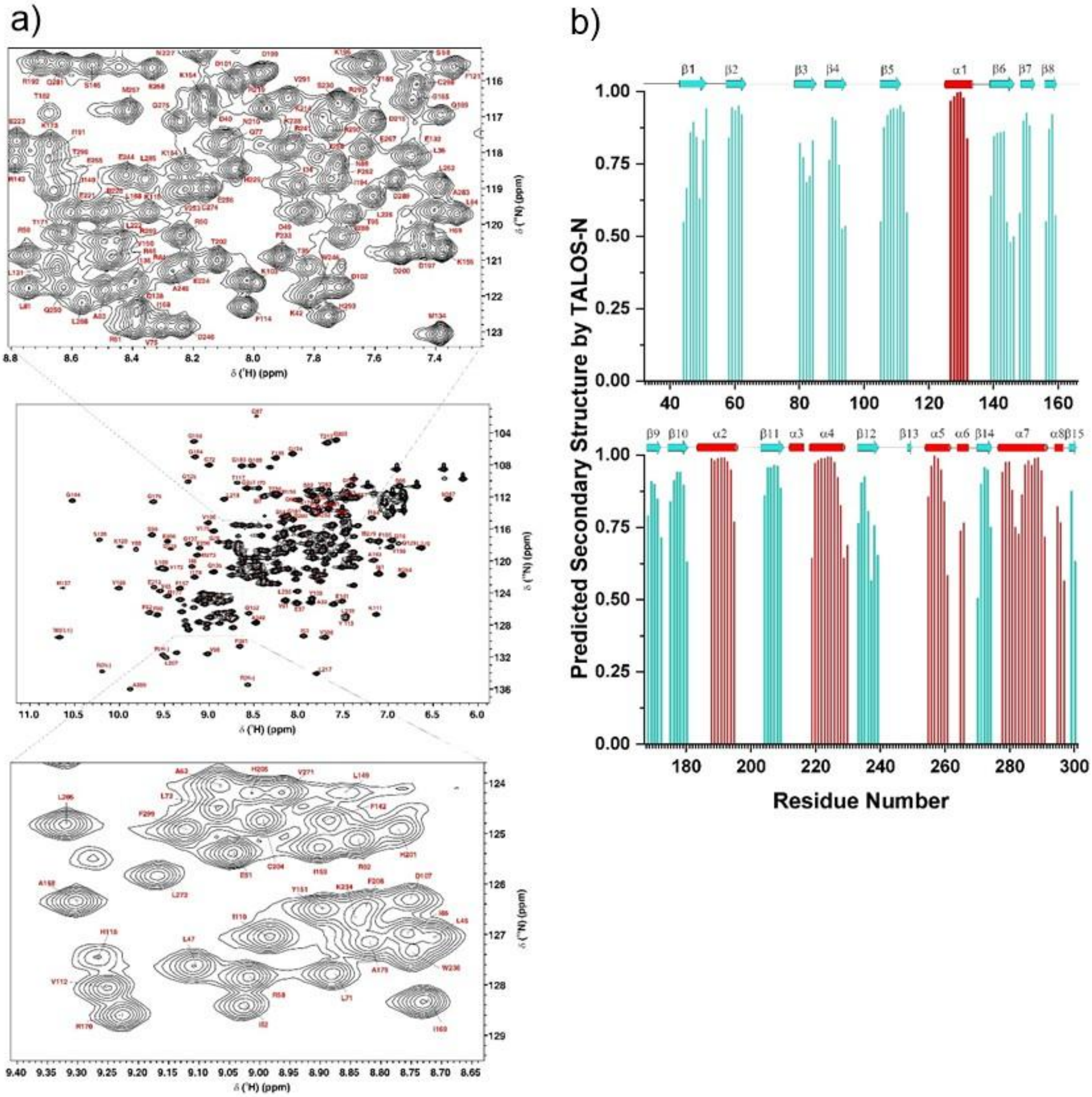

Figure S18. Backbone assignment and secondary structure analysis of FAD-oxidized CYB5R3. **(a)** ^{1}H-^{15}N HSQC NMR spectrum of ^{15}N-labelled FAD-oxidized CYB5R3, recorded at 309 K in 50 mM MOPS, 300 mM NaCl, pH 7.5, is marked with the residue-specific assignment. **(b)** Secondary structure prediction using TALOS-N software) Based on the assigned chemical shifts of the FAD-oxidized CYB5R3. Probabilities of α-helix (red) and β-strand (cyan) secondary structure for each residue are shown. The secondary structures along the sequence extracted from the crystal structure (PDB ID 1UMK) are indicated at the top, where cylinders represent α-helices and arrows represent β-strands.

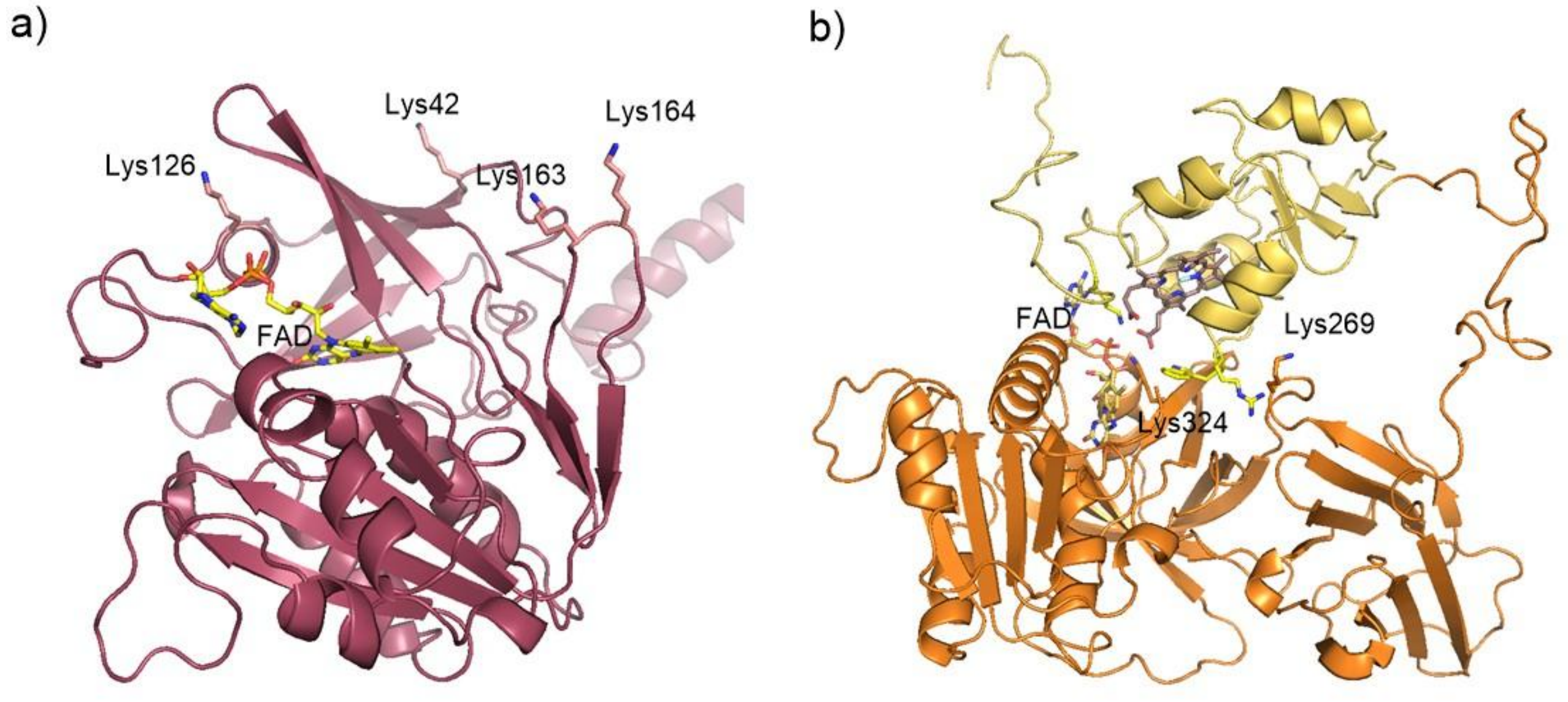

Figure S19. The surface of CYB5R interacting with Cyt-b5. **(a)** The structure shows the surface of CYB5R3 and the residues that interact with Cyt-b5, as confirmed by mutagenesis studies. **(b)** The modelled structure of CYB5R4 shows that the surface above the FAD-binding pocket interacts with the Cyt-b5 domain of CYB5R4.

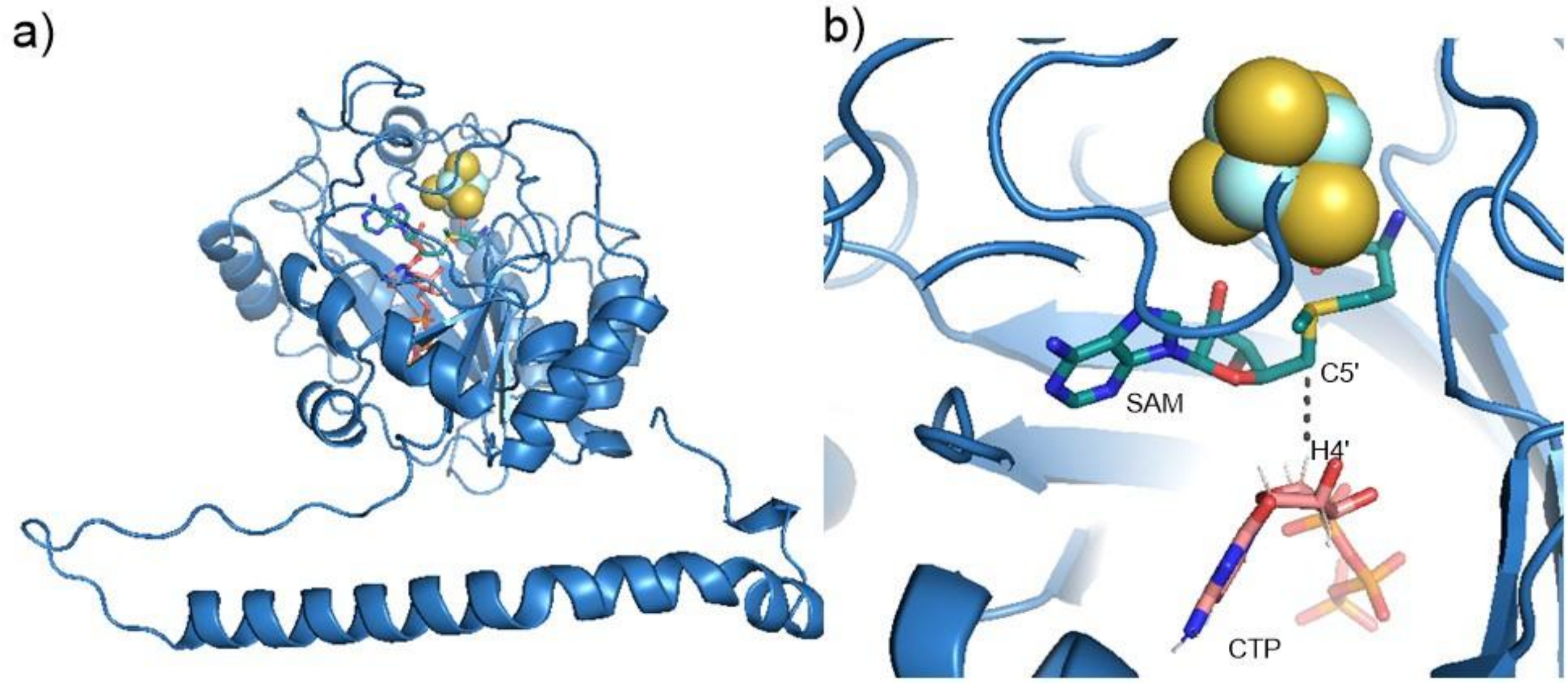

Figure S20. The modelled structure of RSAD2 with CTP and SAM. **(a)** The predicted structure of the RSAD2 with [4Fe-4S] cluster, SAM, and CTP. **(b)** The structure is consistent with the abstraction of H4´ by 5´-dA• radical intermediate.

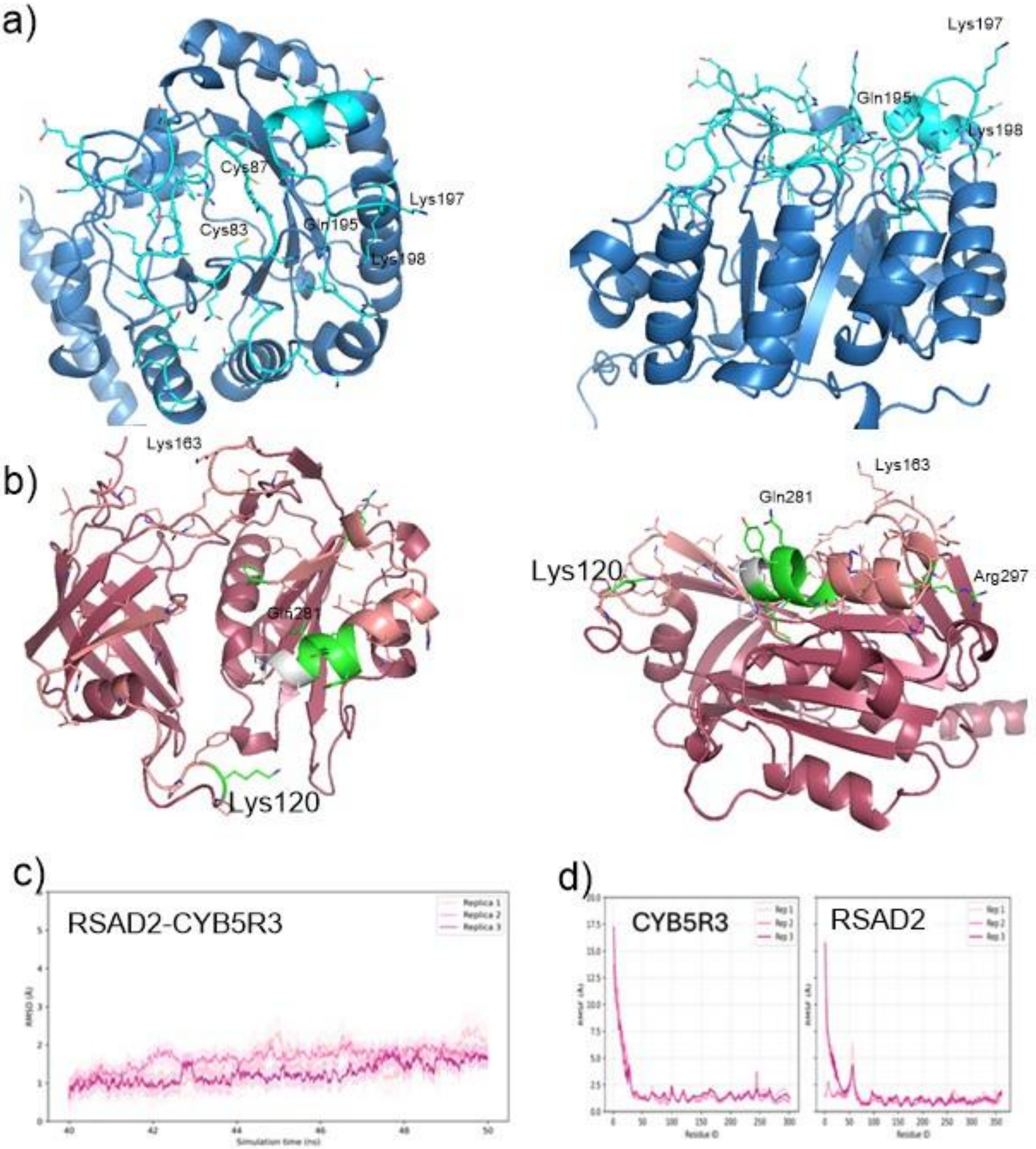

Figure S21. Convergence of three independent MD replicas for the **RSAD2:CYB5R3** complex. **(a)** The surface of RSAD2 above the [4Fe-4S] cluster has the shortest distance to the surface, consistent with electron tunnelling. **(b)** The surface of CYB5R3 interacting with RSAD2, as resolved by NMR spectroscopy. **(c)** RMSD profiles of heavy atoms relative to the initial HADDOCK models over the final 10 ns, computed after backbone alignment (excluding flexible C-terminal tails), illustrate the conformational stability of the complexes. **(d)** Side-chain flexibility of residues in the CYB5R3 and SAND units, represented by the RMSF of heavy atoms, highlights regions of local structural variability within the complexes.

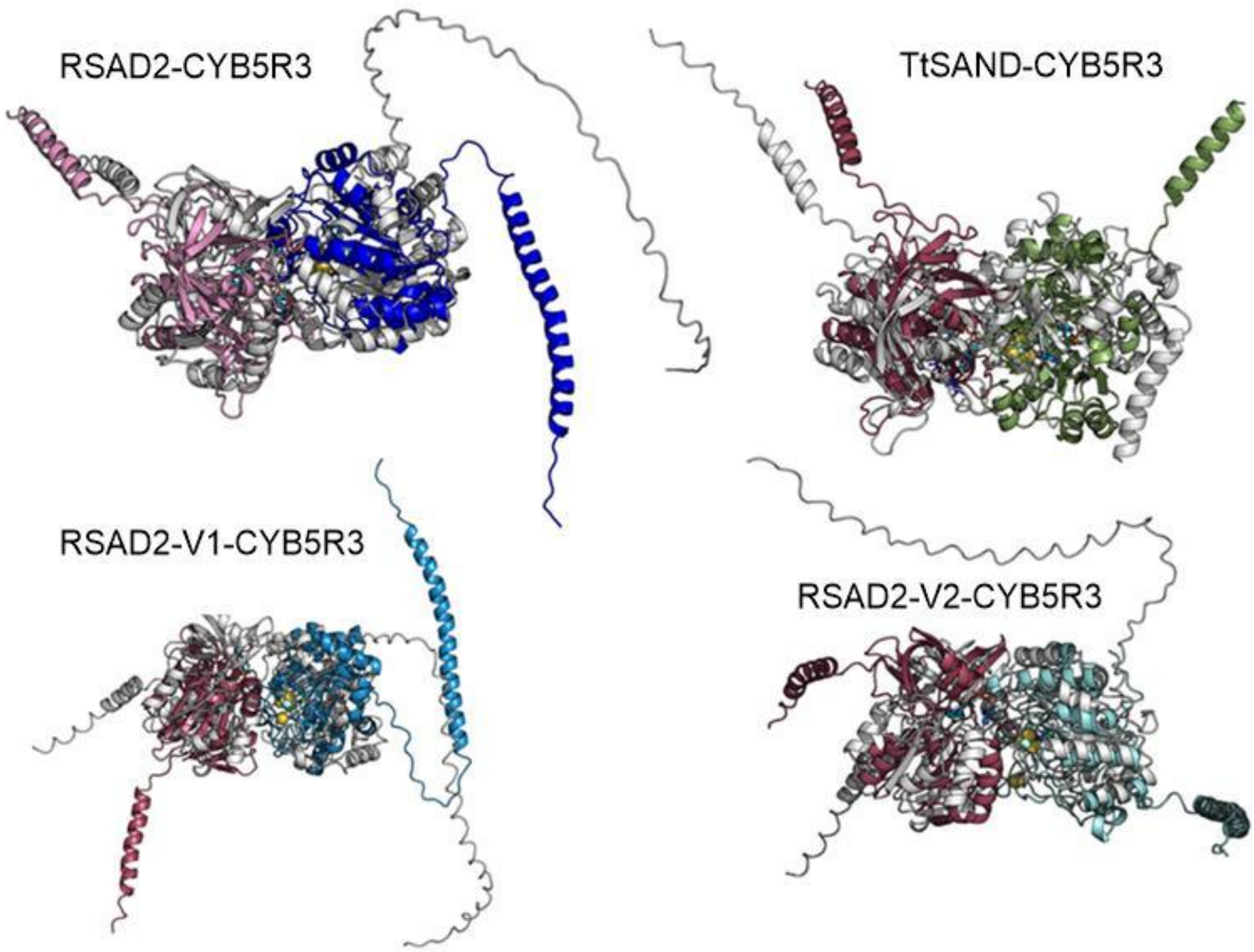

Figure S22. HADDOCK and AlphaFold Multimer predictions are consistent. To assess the HADDOCK predictions, we generated additional structural models of the complexes using AlphaFold Multimer. The following figure compares the AlphaFold Multimer predictions (in grey cartoon representation) and the HADDOCK predictions (coloured cartoon representations) and shows that both approaches predict complexes with similar interaction surfaces.

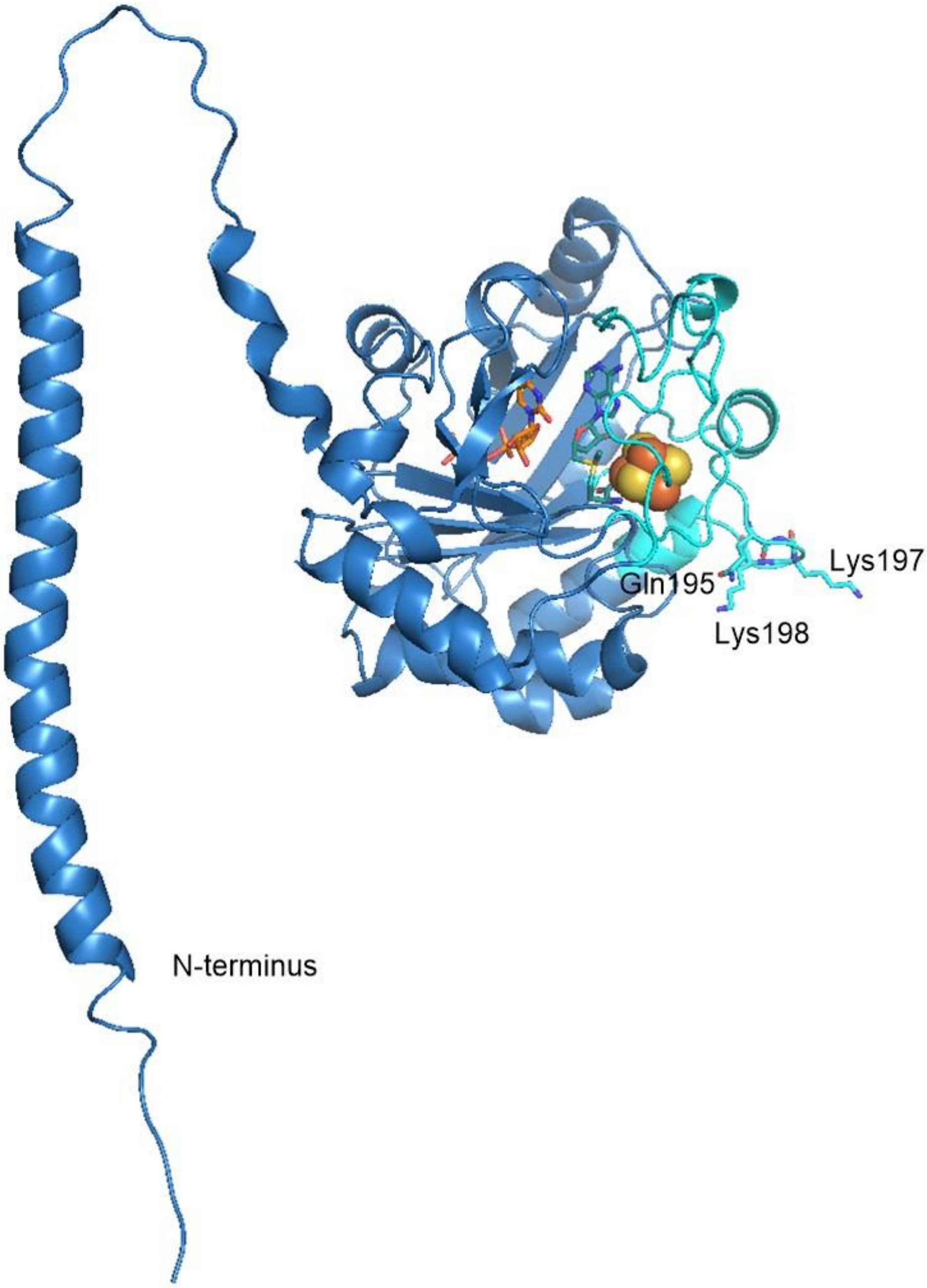

Figure S23. The figure shows the RSAD2 surface predicted to interact with CYB5R3. Considering the location and possible orientations of the N-terminus amphipathic domain localising the protein to the ER membrane, this surface is perfectly available to interact with CYB5R3. It is the surface with the shortest distance to the [4Fe-4S] cluster.

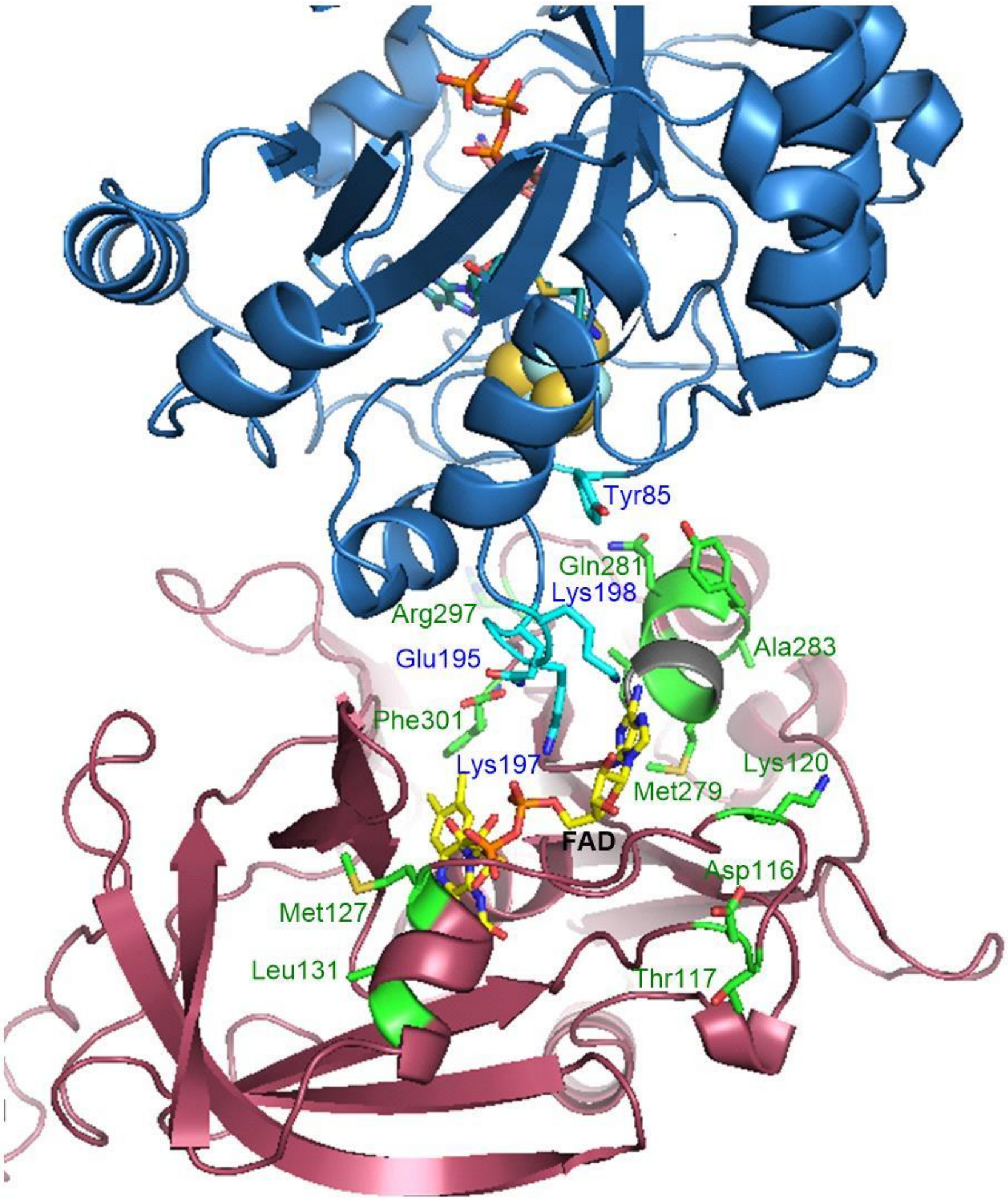

Figure S24. The interaction of CYB5R3 with RSAD2. In the figure we have highlighted the amino acid residues 276-284 (green) in a short α-helix of CYB5R3 that directly interacts with RSAD2. The grey residues are proline.

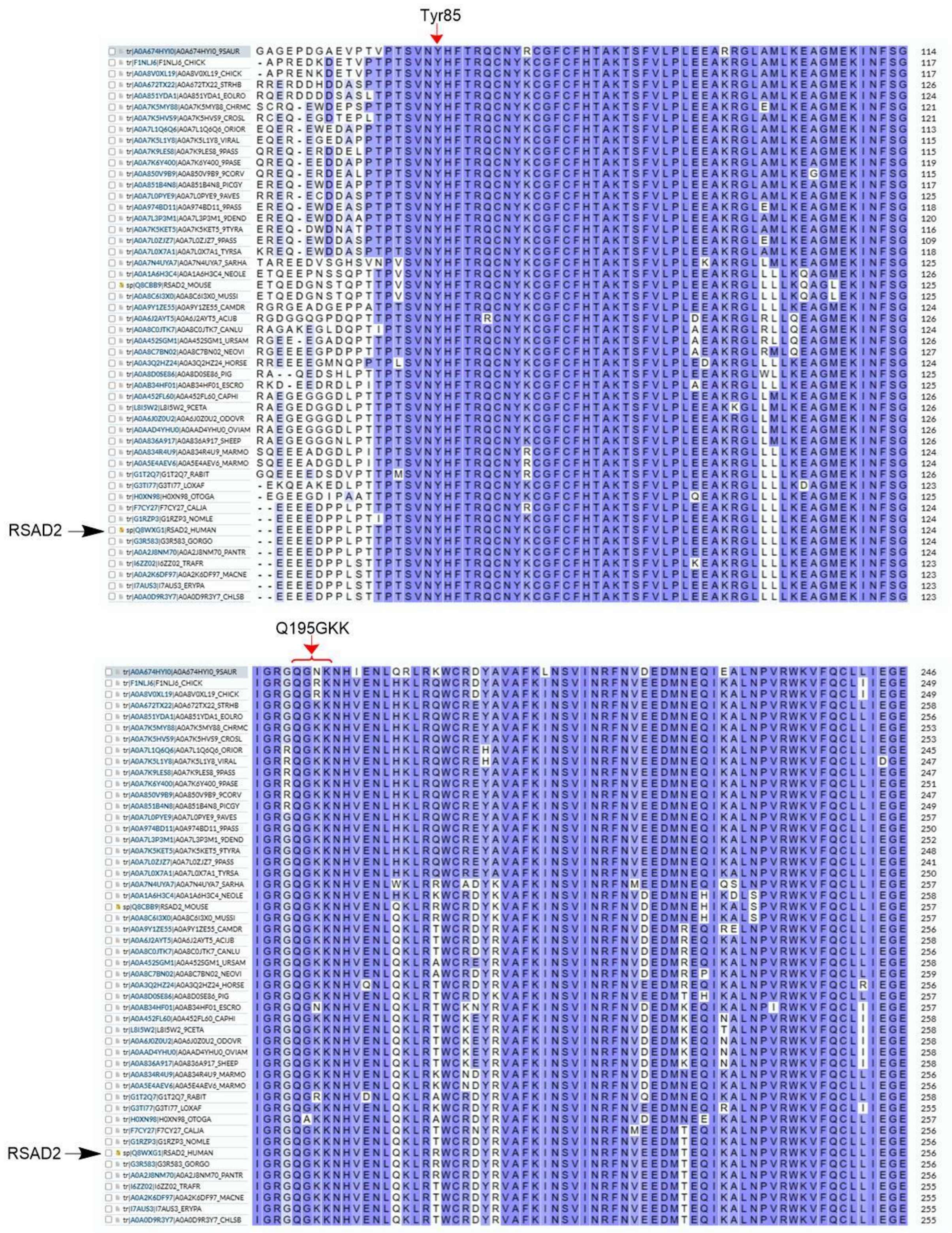

Figure S25. The RSAD2 Tyr85 (top) and surface loop (bottom) are highly conserved in eukaryotes.

a) MoaA

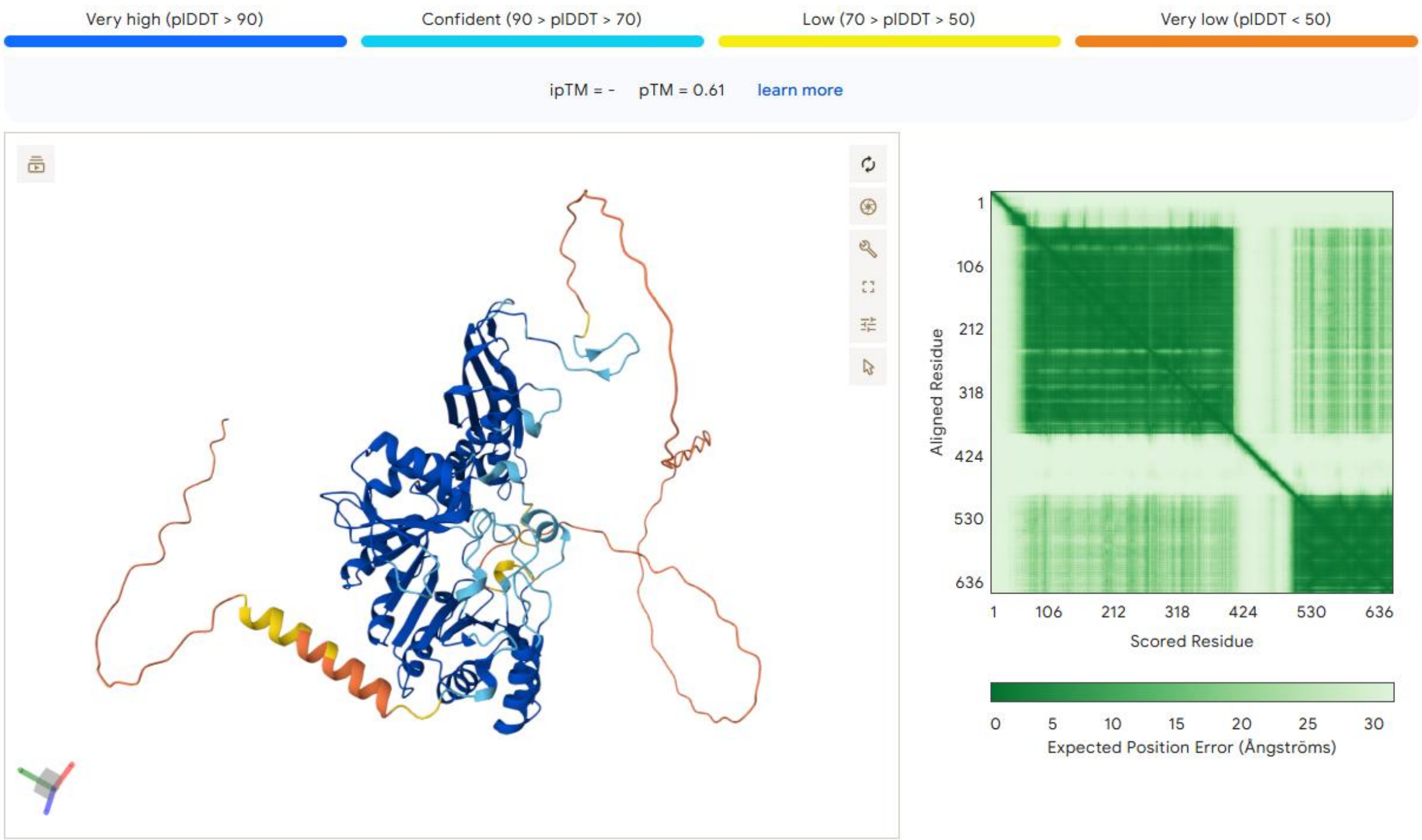
Very high (pIDDT > 90)
Confident (90 > pIDDT > 70)
Low (70 > pIDDT > 50)
Very low (pIDDT < 50)
ipTM = -   pTM = 0.61   learn more
Aligned Residue
1
106
212
318
424
530
636
Scored Residue
0
5
10
15
20
25
30
Expected Position Error (Ångströms)

b) TYW1

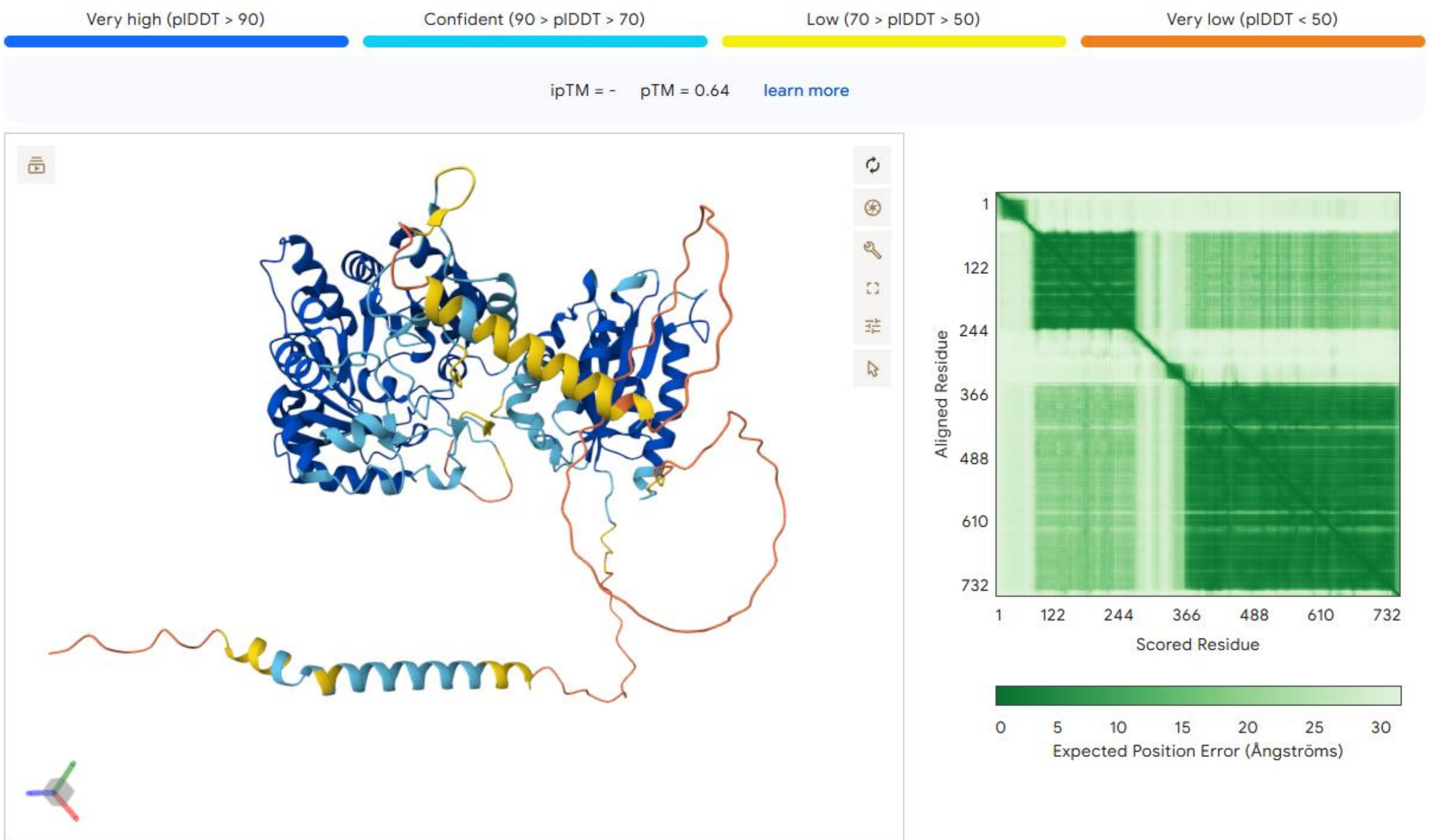
Very high (pIDDT > 90)
Confident (90 > pIDDT > 70)
Low (70 > pIDDT > 50)
Very low (pIDDT < 50)
ipTM = -   pTM = 0.64   learn more
Aligned Residue
1
122
244
366
488
610
732
Scored Residue
0
5
10
15
20
25
30
Expected Position Error (Ångströms)

c) LIAS

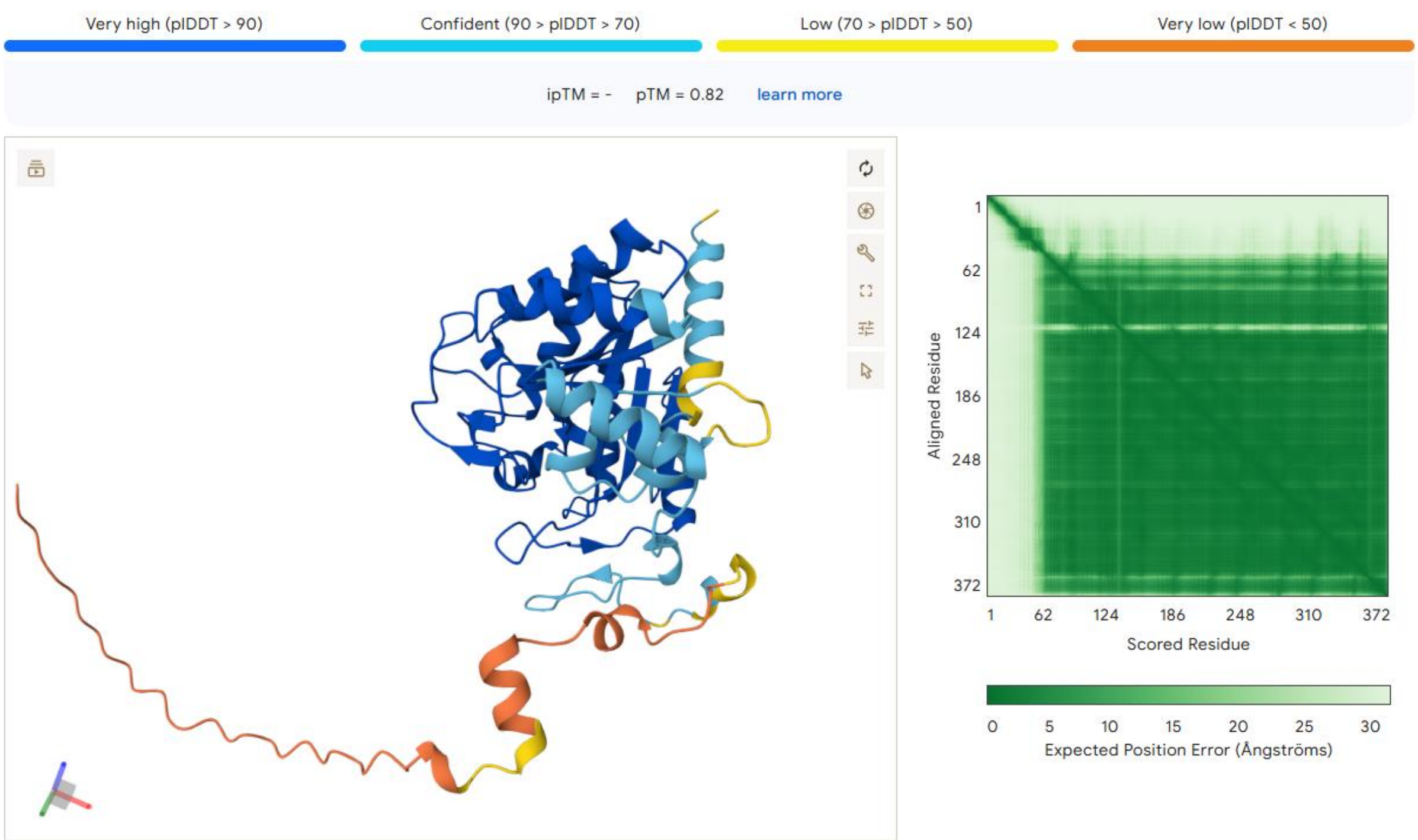

d) RSAD1

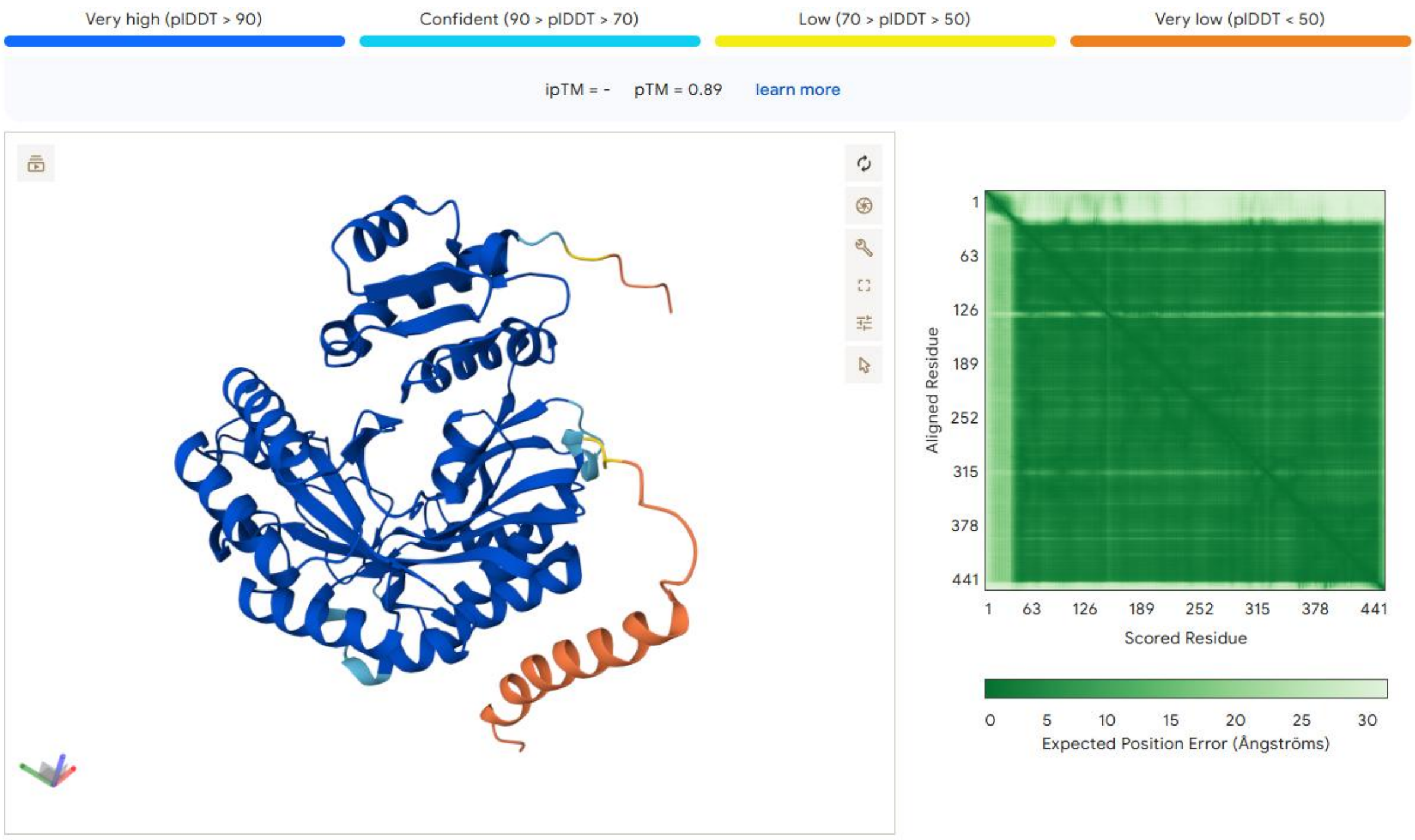

e) MiaB

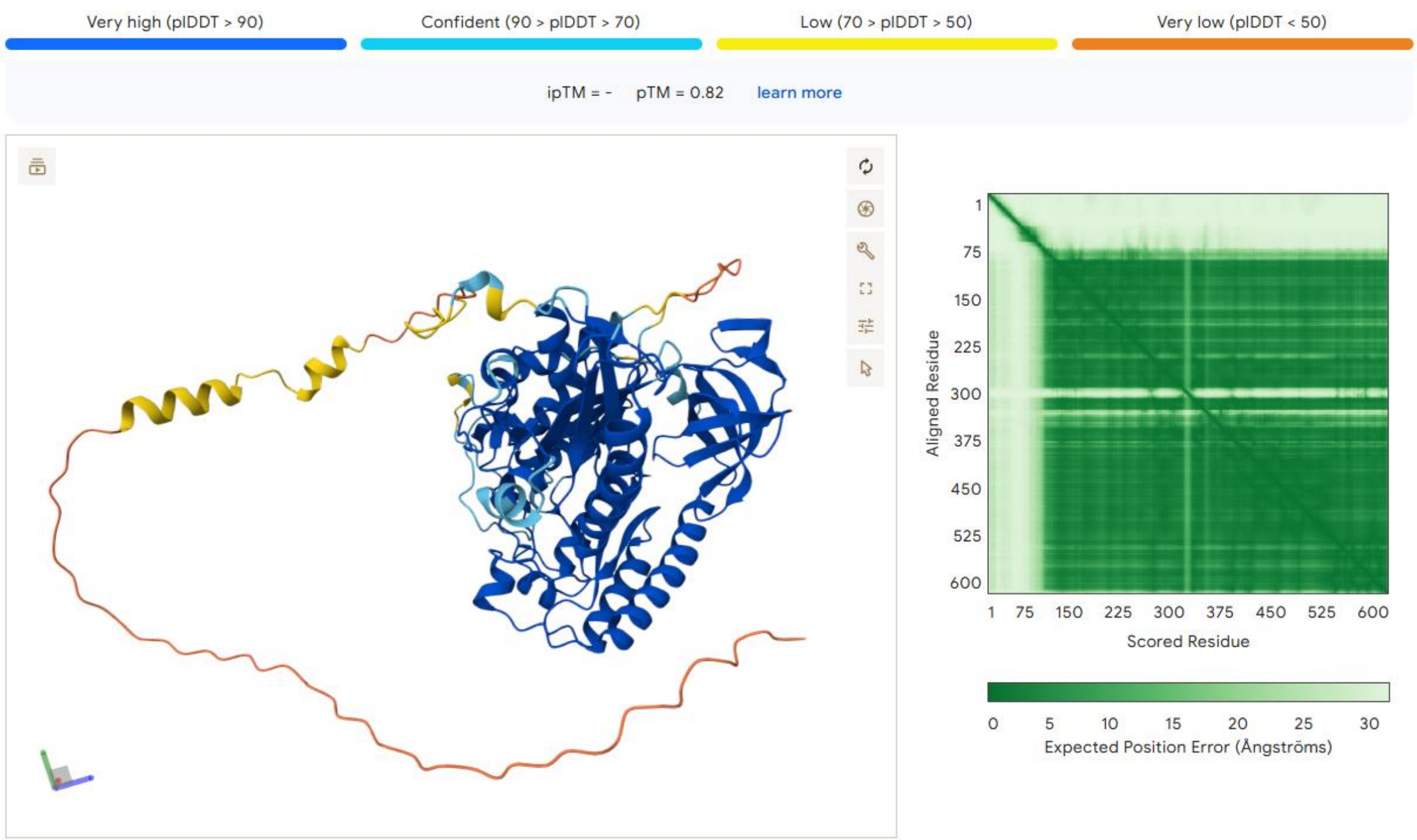

f) MtaB

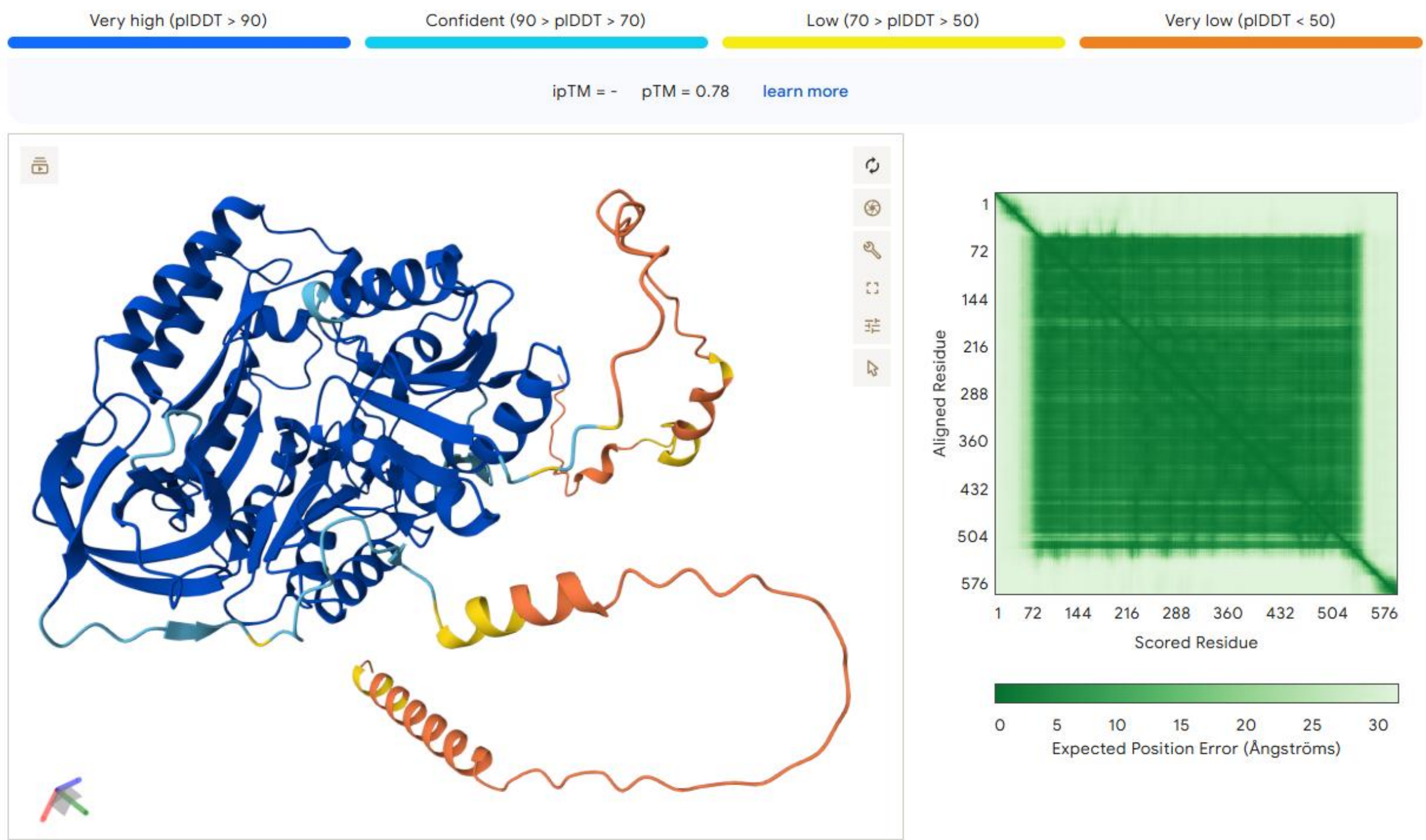

Figure S26. AlphaFold predicted models of human RSAD2, including the pLDDT graphs. The AlphaFold predicted models of **(a)** MoaA (MOCS1A), **(b)** TYW1, **(c)** LipA (LIAS), **(d)** RSAD1, **(e)** MiaB (CDK5RAP1), and **(f)** MtaB (CDKAL1).

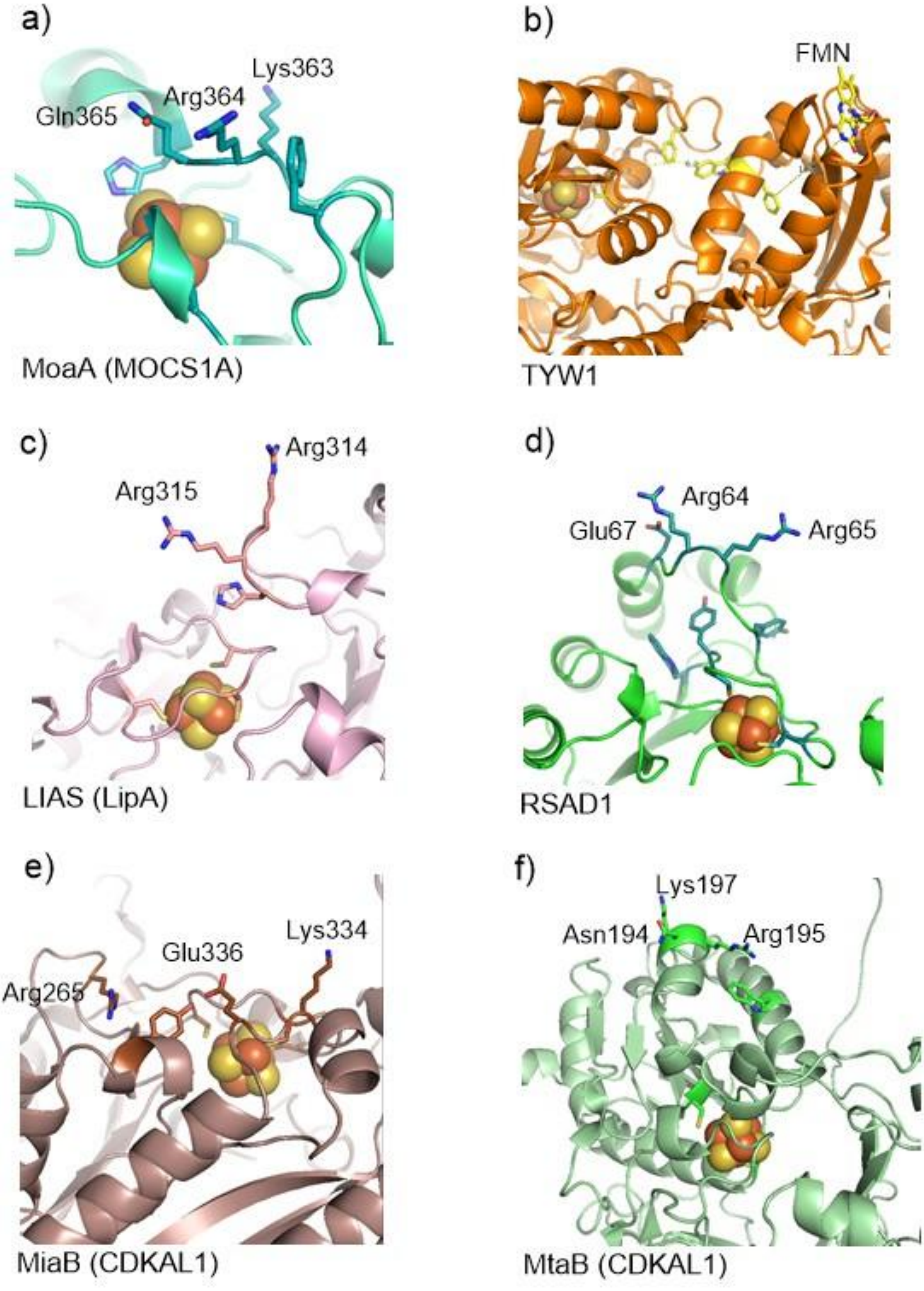

Figure S27. Analysis of the predicted structure of human radical-SAM enzymes. The analysis suggests the presence of a loop and an aromatic residue such as tyrosine, histidine, or phenylalanine. Alternatively, some radical-SAM enzymes, such as TYW1, are predicted to have an FMN-binding domain similar to that of flavodoxin in bacteria. The structures were predicted using AlphaFold and AlphaFill. Six human radical-SAM enzymes are analysed **(a)** MoaA (MOCS1A), **(b)** TYW1, **(c)** LipA (LIAS), **(d)** RSAD1, **(e)** MiaB (CDKAL1), and **(f)** MtaB (CDKAL1).

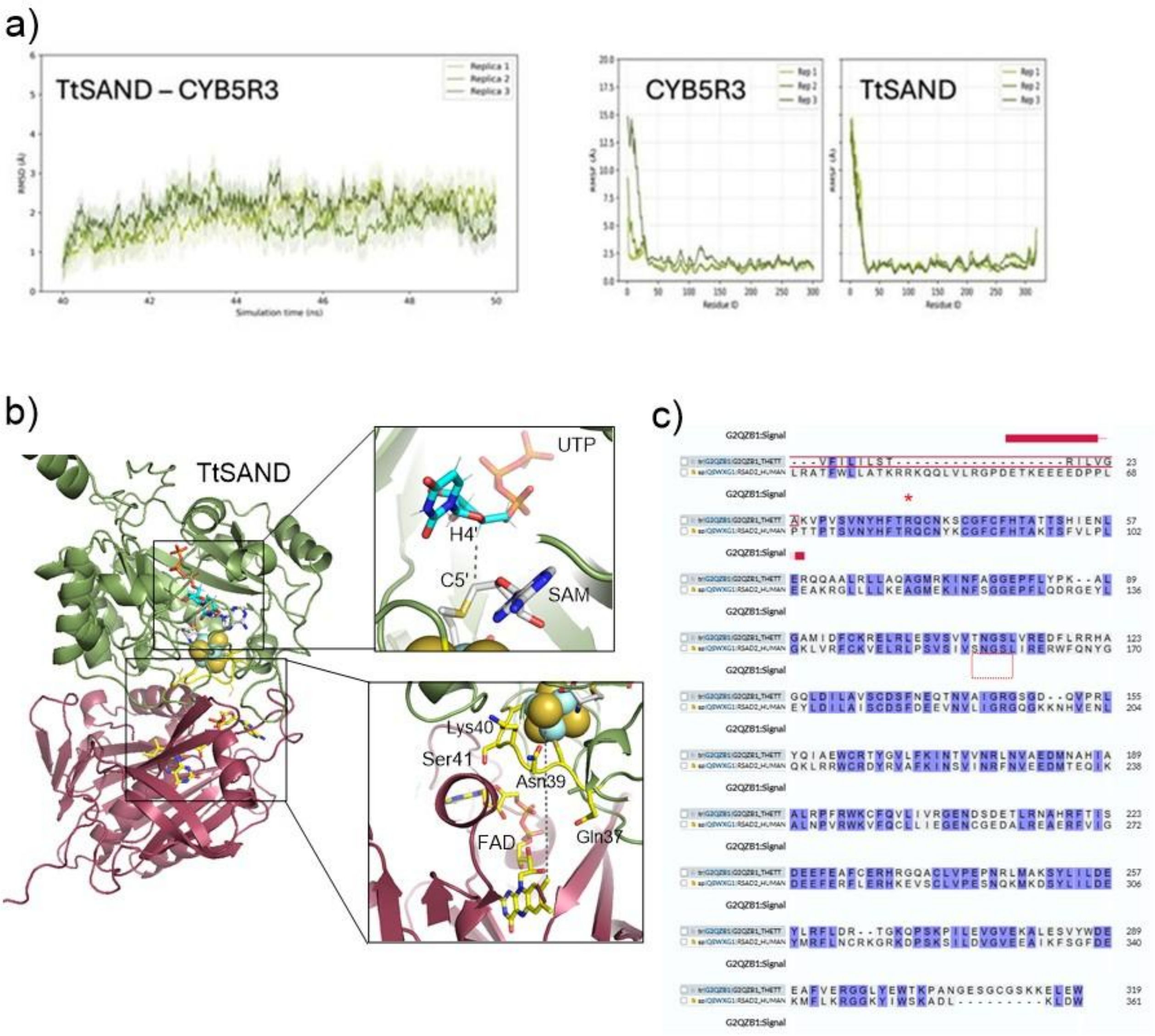

Figure S28. The modelled complex between WT-TtSAND with CYB5R3. **(a)** RMSD profiles of heavy atoms relative to the initial HADDOCK models over the final 10 ns, computed after backbone alignment (excluding flexible C-terminal tails), illustrate the conformational stability of the complexes. **(b)** The modelled structure of fungal TtSAND in complex with CYB5R3. The structure shows the orientation of H4´ in UTP towards C5´ in SAM, which is consistent with the formation of ddhUTP as described previously.[3] The preferred substrate of TtSAND is UTP. The structure shows that the QGKK loop and tyrosine are absent in TtSAND. **(c)** Aligning the amino acid residues of RSAD2 and TtSAND shows that Tyr85 (red star) and Q195GKK loop (red box) in RSAD2 are absent in TtSAND.

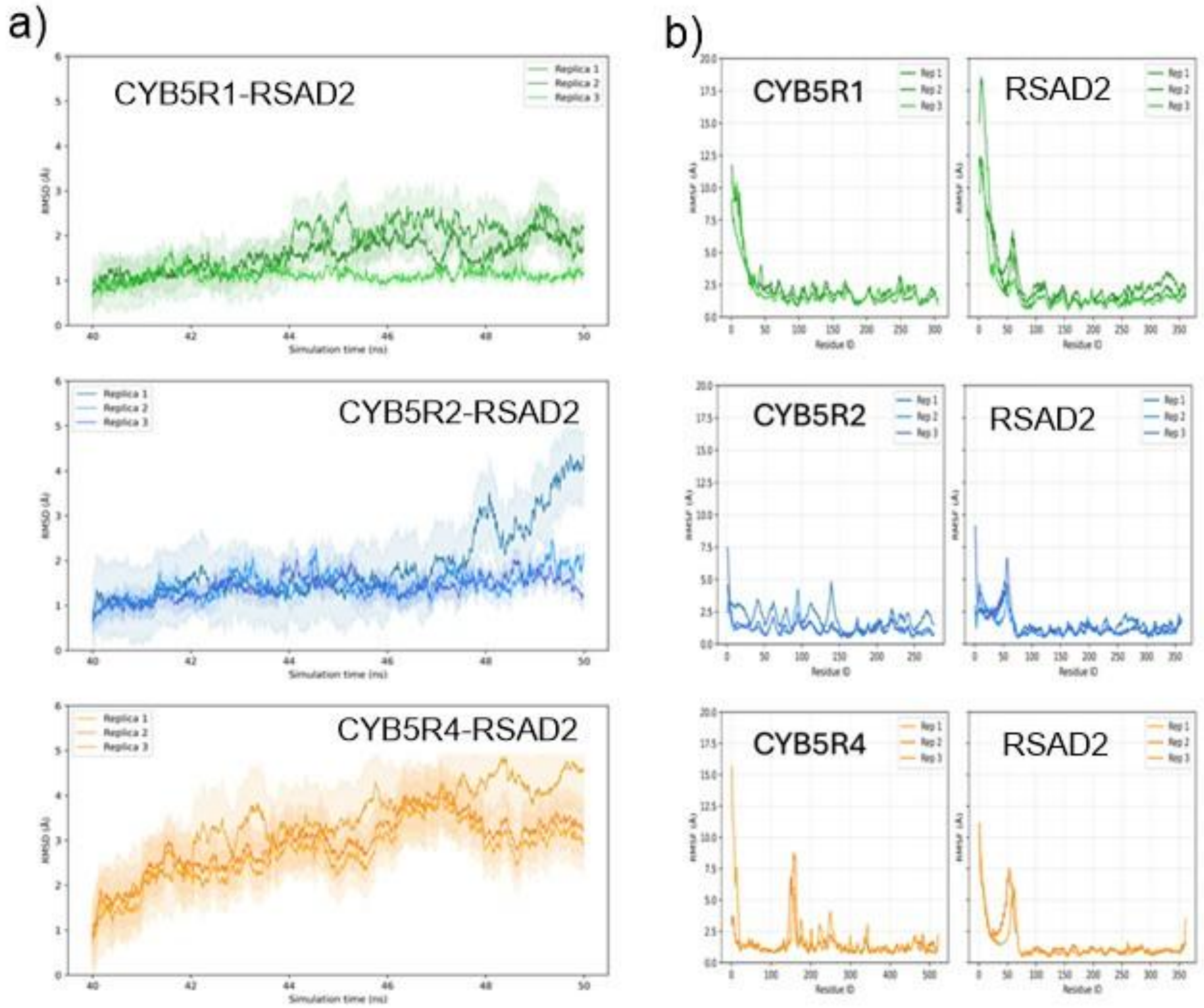

Figure S29. Convergence of three independent MD replicas for the RSAD2:CYB5R1, RSAD2:CYB5R2, and RSAD2:CYB5R4 complexes. **(a)** RMSD profiles of heavy atoms relative to the initial HADDOCK models over the final 10 ns, computed after backbone alignment of the three models (excluding flexible C-terminal tails), illustrate the conformational stability of the complexes. **(b)** Side-chain flexibility of residues in the CYB5Rs and RSAD2 units, represented by the RMSF of heavy atoms, highlights regions of local structural variability within the complexes.

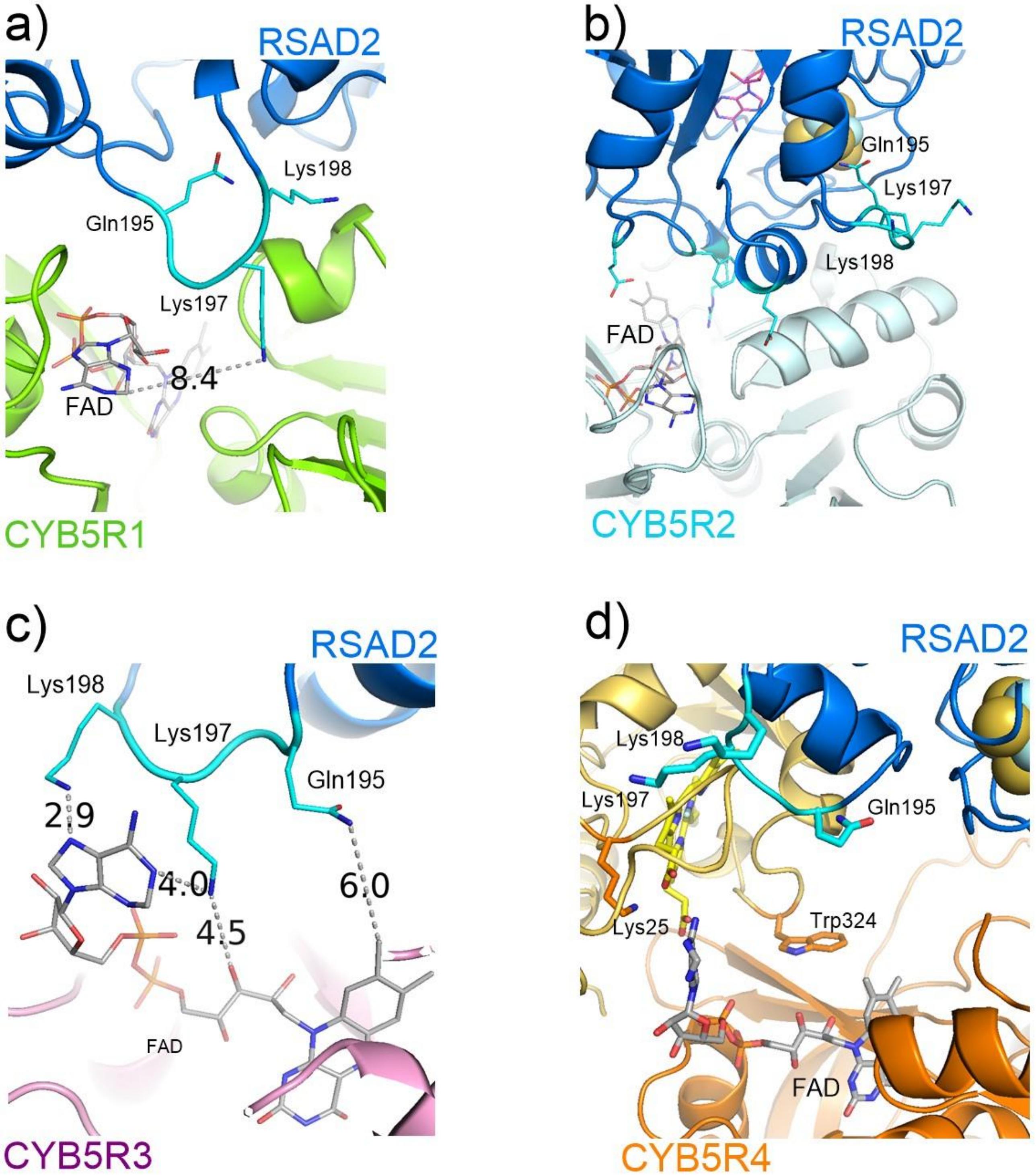

Figure S30. The RSAD2 $Q_{195}GKK$ surface loop interacts with the FAD cofactor in CYB5R3 but not with the FAD cofactor in other CYB5R members. The energy-optimized structure of **(a)** CYB5R1/RSAD2, **(b)** CYB5R2/RSAD2, **(c)** CYB5R3/RSAD2, and **(d)** CYB5R4/RSAD2.

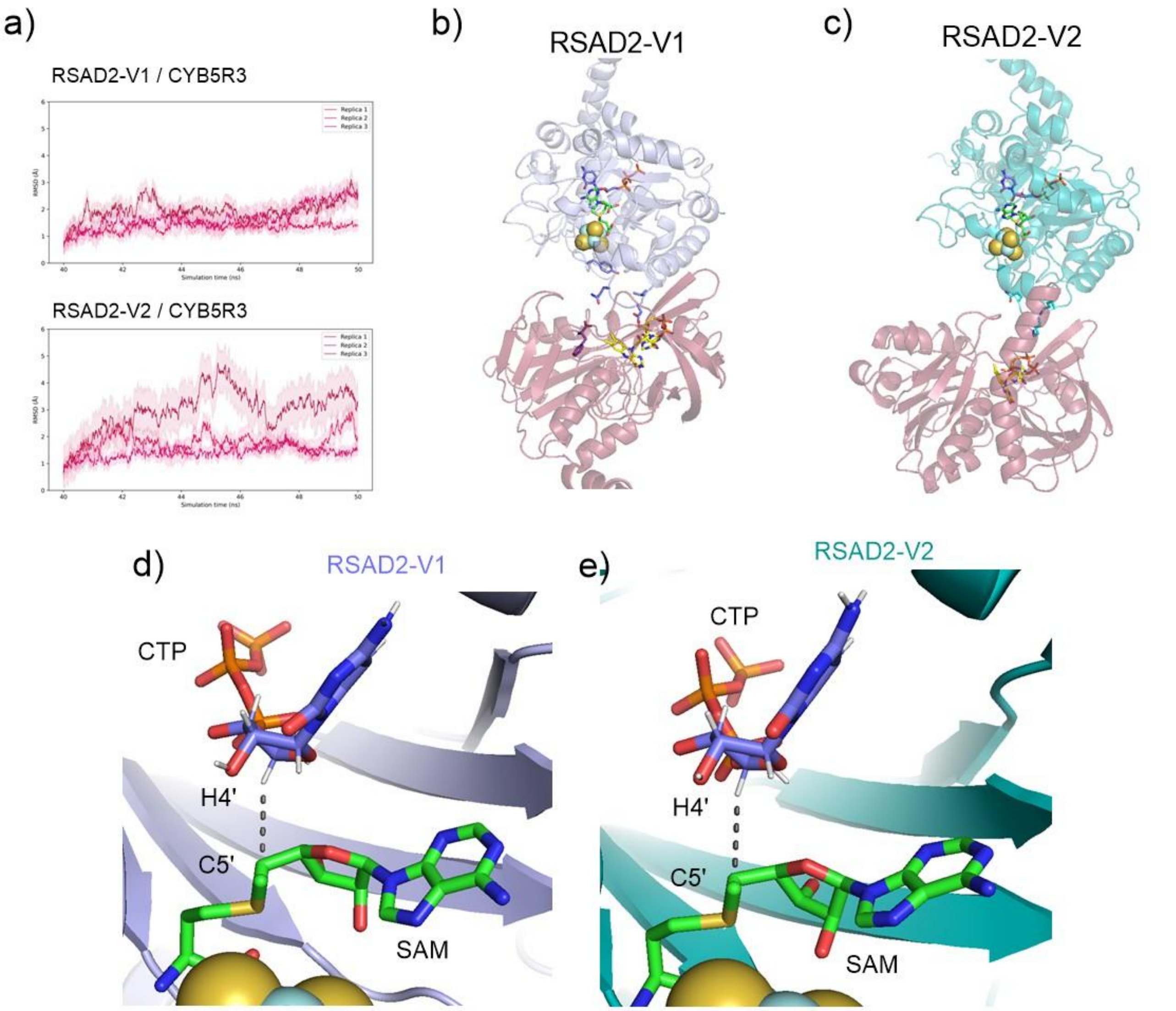

Figure S31. Convergence of three independent MD replicas for the RSAD2-V1:CYB5R3 and RSAD2-V2:CYB5R3 complexes. **(a)** RMSD profiles of heavy atoms relative to the initial HADDOCK models over the final 10 ns, computed after backbone alignment of the three models (excluding flexible C-terminal tails), illustrate the conformational stability of the complexes. **(b)** The modelled structure of CYB5R3/RSAD2-V1 and **(c)** CYB5R3/RSAD2-V2 (Tyr85A) complexes. **(d)** The predicted position of CTP in CYB5R3/RSAD2-V1 and **(e)** CYB5R3/RSAD2-V2.

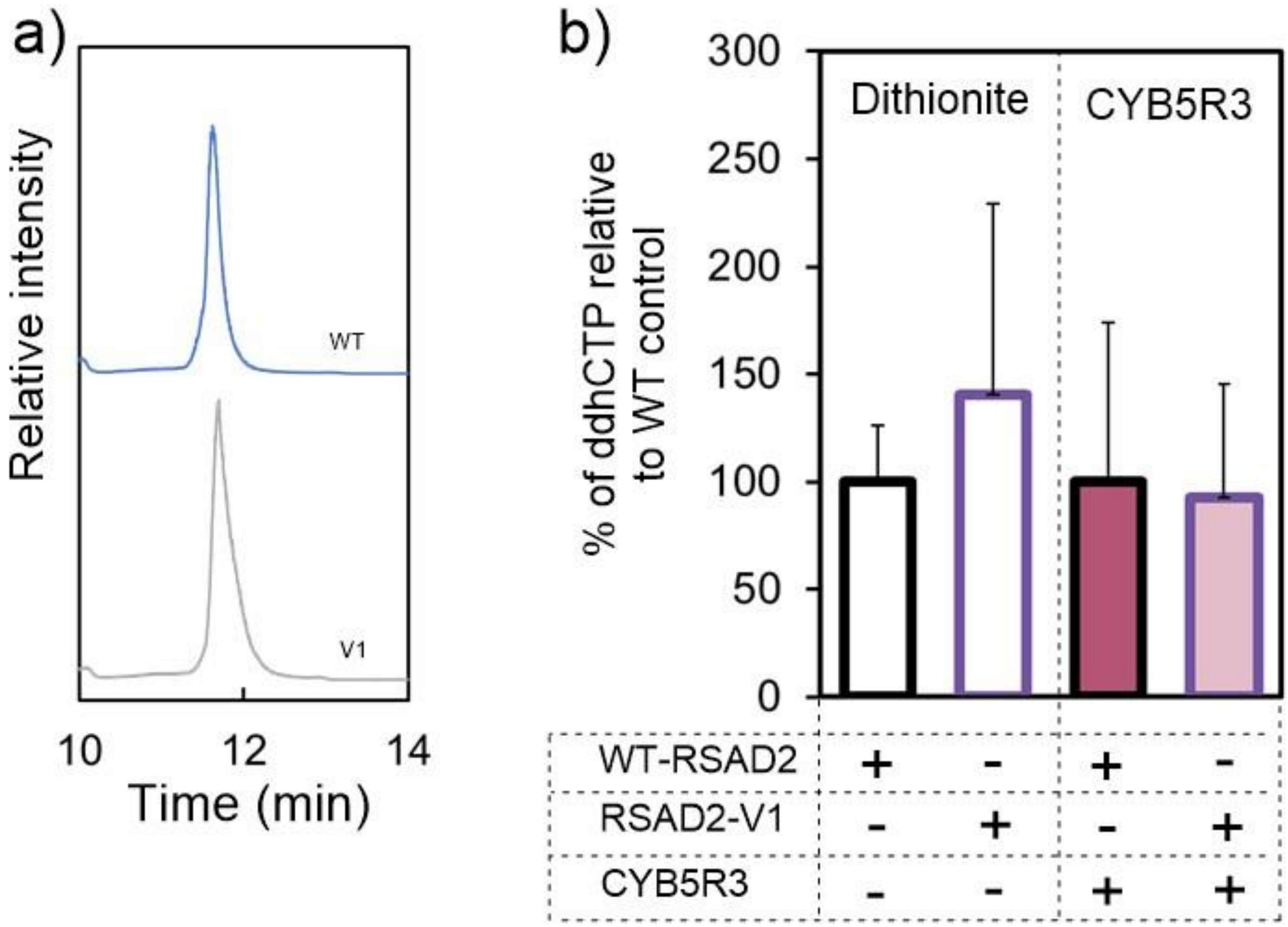

Figure S32. The effect of RSAD2 surface loop or Tyr85 mutations on [4Fe-4S] cluster activity in vitro and inside cells. **(a)** An example of an extracted ion chromatogram (EIC) for ddhCTP $[M\text{-}H]^{-1}$ with a m/z 464.0 using dithionite as a reducing agent. The concentration of the wild-type (WT) or V1 variant (V1) was 53 µM. Each reaction contained CTP (5.3 mM), SAM (21.2 mM), and sodium dithionite (5.3 mM). **(b)** Relative % of ddhCTP formation for WT-RSAD2 and RSAD2-V1 in the presence of dithionite as a reducing agent or CYB5R3 and NADH as an electron donor. The concentration of the wild-type (WT) or V1 variant (V1) was 53 µM. Concentrations of CTP, SAM, and sodium dithionite were 5.3 mM, 21.2 mM, and 5.3 mM, respectively. The final buffer was 50 mM MOPS buffer, pH 7.0, containing 100 mM NaCl. Data are the average of two independent measurements ± errors.

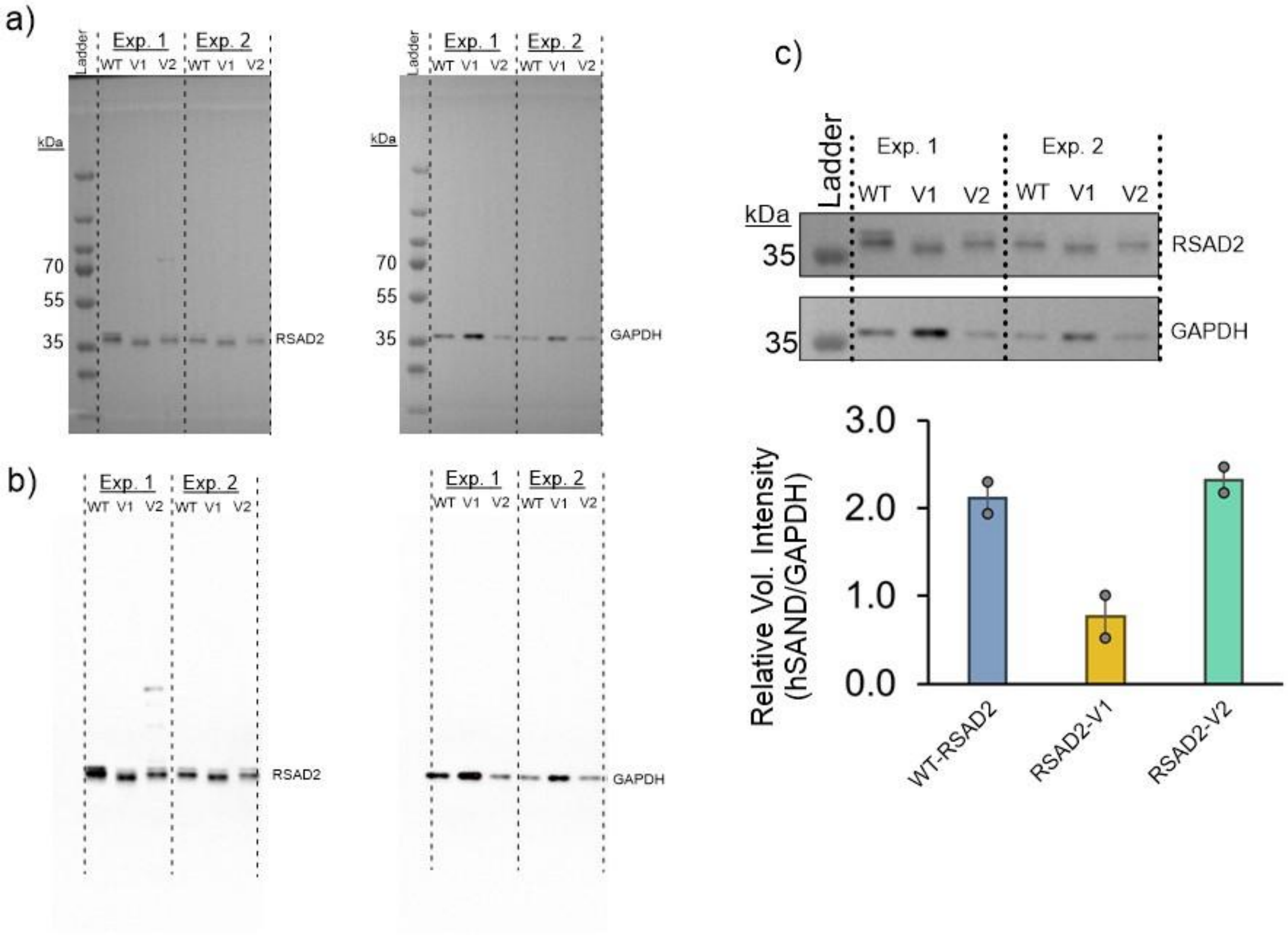

Figure S33. Western blot analysis of the expression of WT-RSAD2 and its variants. **(a)** An overlay of the Chemi-blot and the image of the ladder, and **(b)** the Chemi-blot itself. All images were taken by the iBright system, and the system prepared the overlay. Two independent expressions of proteins (Exp. 1 and Exp. 2) were analysed. The ladder was Thermo Scientific™ PageRuler™ Plus Prestained Protein Ladder, 10 to 250 kDa. **(c)** Western blot analysis of his-tagged WT-RSAD2, RSAD2-V1, and RSAD2-V2 expression. Blots from two biologically independent experiments (Exp.1 and Exp.2) are shown. The volumetric (Vol.) intensity of the bands relative to GAPDH was determined using iBright software and plotted as a bar chart.

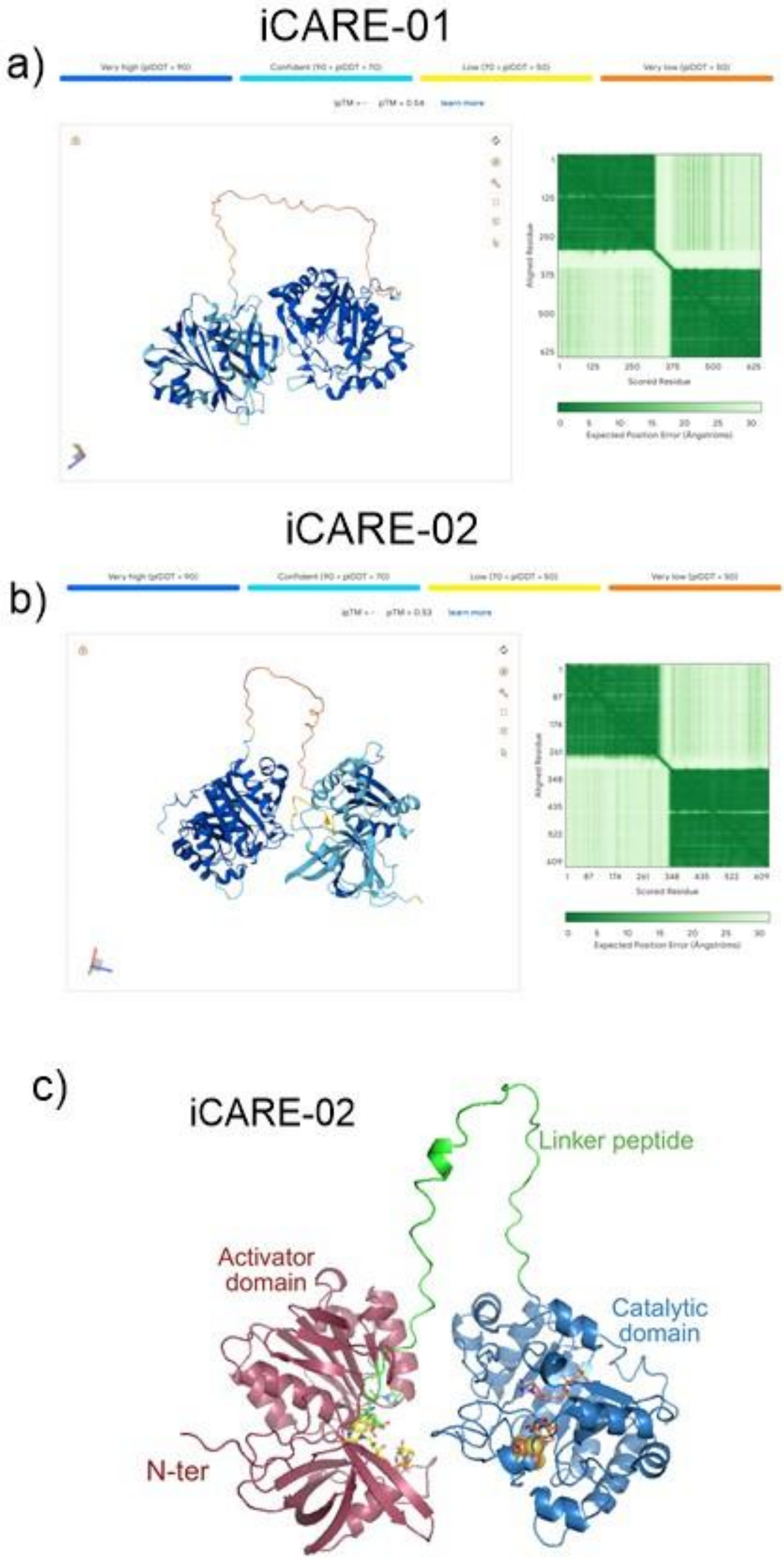

Figure S34. The AlphaFold predicted structure of iCAREs, including pLDDT graphs. The AlphaFold predicted models of **(a)** iCARE-01 and **(b)** iCARE02. The charts show that the predictions have low or very low confidence in the fusion peptide between the two domains (hRSAD2 domain) and (NADH reductase domain). **(c)** The AlphaFold predicted structure of iCARE-02, which was created by fusing the C-terminus of CYB5R3 (activator domain) to the N-terminus of RSAD2 (catalytic domain).

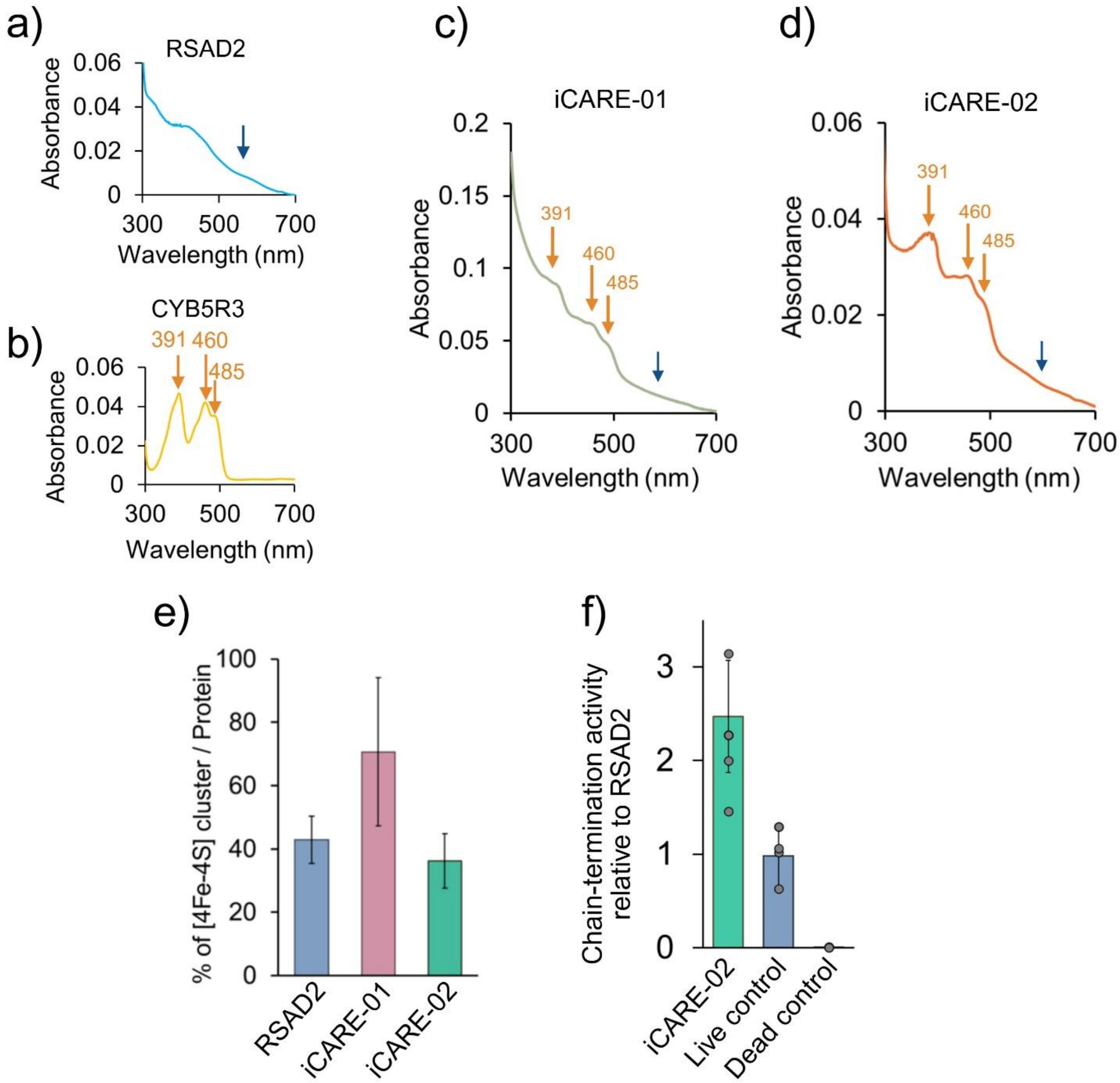

Figure S35. Characterisation of eCAREs. **(a)** UV-visible absorbance spectrum of wild-type RSAD2 (4 µM) and **(b)** that of CYB5R3 (1.1 µM). **(c)** The UV-visible absorbance spectra of iCARE-01 (3.5 µM) and **(d)** that of iCARE-02 (10 µM)**. (e)** Estimation of [4Fe-4S] cluster content per protein for wild-type RSAD2, iCARE-01, and iCARE-02. **(f)** Chain termination activity of iCARE-02 relative to RSAD2 as determined using equation 3, relative to live control (cells co-expressing RSAD2 and CYB5R3, and LdrD) and dead control (cells only expressing LdrD). The data are the average of four biologically independent measurements ± standard deviation.

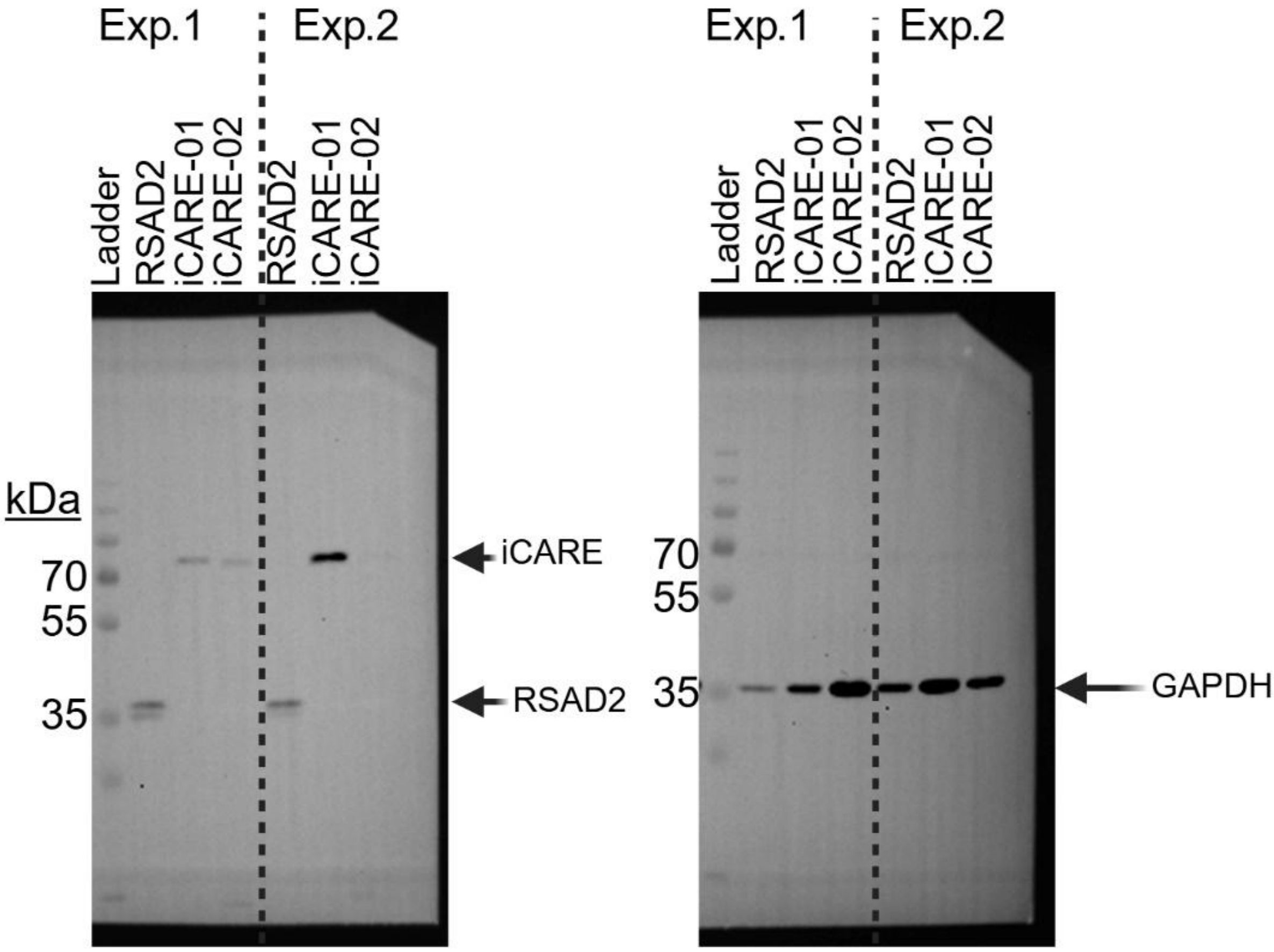

Figure S36. Western blot analysis of the expression of WT-RSAD2 and iCAREs. An overlay of the Chemi-blot and the image of the ladder for two expression (Exp.) repeats. HRP-conjugated His-tag antibody was used to detect proteins. GAPDH antibody was used as a loading control. All images were captured using the iBright system, which prepared the overlay. The ladder was Thermo Scientific™ PageRuler™ Plus Prestained Protein Ladder, 10 to 250 kDa.

Table S1. The confidence metrics, pTM and ipTM, for the predicted AlphaFold structures are described in the manuscript.

AlphaFold Model	Figures	pTM	ipTM
CYB5R1	S1	0.88	null
CYB5R2	S1	0.93	null
CYB5R3	S1	0.90	null
CYB5R4	S1	0.68	null
RSAD2	S1	0.81	null
LipA/LIAS	S25	0.82	null
MiaB/CDK5RAP1	S25	0.82	null
MoaA/MOCS1A	S25	0.61	null
MtaB/CDKAL1	S25	0.78	null
RSAD1	S25	0.89	null
TYW1	S25	0.64	null
RSAD2-CYB5R3	S20	0.571	0.33
RSAD2-V1-CYB5R3	S20	0.55	0.275
RSAD2-V2-CYB5R3	S20	0.52	0.208
TtSAND-CYB5R3	S20	0.565	0.301
iCARE-01	S31	0.54	null
iCARE-02	S31	0.53	null

ipTM is the interface for Predicted Template Modelling and is obtained when the complex of two proteins is modelled. Null: not available because one protein was modelled.

Table S2. ProteinPromp scores were obtained for the interactions between each human radical-SAM enzyme and four members of the CYB5R family.

**Radical SAM**	**CYB5R1**	**CYB5R2**	**CYB5R3**	**CYB5R4**
RSAD2 (viperin)	0.252	0.413	0.416	0.196
Elp3	0.332	0.480	0.516	0.390
LipA/LIAS	0.233	0.385	0.401	0.134
MiaB/CDK5RAP1	0.263	0.481	0.488	0.250
MoaA/MOCS1A	0.270	0.458	0.461	0.214
MtaB/CDKAL1	0.206	0.370	0.425	0.202
RSAD1	0.213	0.396	0.433	0.16
TYW1	0.213	0.382	0.440	0.182

The closer the score to 1, the more likely the binding. CYB5R3 and then R2 show the highest scores for all the radical-SAM enzymes.

Table S3. Analysis of ddhCTP formation in *E. coli* cells.

	CYBR1	RSAD2	R1+RSAD2
test 1	-0.296	0.392	0.734
test 2	0.206	0.538	0.234
test 3	-0.001	0.400	0.632
test 4	0.162	0.590	1.158
test 5	-0.071	0.452	0.777

	CYBR2	RSAD2	R2+RSAD2
test 1	0.354	-0.163	0.346
test 2	-0.286	0.247	-0.089
test 3	0.246	-0.064	0.416
test 4	-0.373	-0.368	0.446
test 5	0.304	0.228	0.772

	CYBR3	RSAD2	R3+RSAD2
test 1	-0.064	0.394	2.954
test 2	-0.064	0.455	2.995
test 3	-0.204	0.394	1.627
test 4	-0.364	-0.539	4.425
test 5	0.550	-0.118	4.726

	CYBR4	RSAD2	R4+RSAD2
test 1	0.038	0.810	1.637
test 2	-0.099	0.425	0.095
test 3	0.210	0.307	0.463
test 4	-0.121	0.440	-0.511
test 5	-0.027	0.047	0.148

ddhCTP formation: retention time 11.5 min and parent ion $[M-H]^{-1}$ m/z = 464.00. The area under the curve is calculated relative to the background area under the curve for the cells expressing only CYB5R, under conditions which ddhCTP is not produced because RSAD2 is absent.

Table S4. Protein–protein interaction energy terms estimated by HADDOCK for the RSAD2:CYB5R complexes, together with the predicted Buried Surface Area (BSA).

	**Binding energy (kcal/mol)**	**Buried Surface Area (Å^2)**	**Van der Waals energy (kcal/mol)**	**Electrostatic energy (kJ/mol)**
**RSAD2 – CYB5R1**	-463.80	2674.19	-47.47	-575.16
**RSAD2 – CYB5R2**	-538.57	2995.66	-91.30	-609.84
**RSAD2 – CYB5R3**	-396.16	3211.04	-82.0	-419.4
**RSAD2-V1-CYB5R3**	-592.241	3698.89	-106.152	-106.152
**RSAD2-V2-CYB5R3**	-426.17	3175.07	-87.44	-481.76
**RSAD2 – CYB5R4**	-465.587	2465.19	-57.2469	-574.05
**TtSANd – CYB5R3**	-393.02	2833.55	-69.19	-450.83

Table S5. Average RMSD values of heavy atoms of the SAND:CYB5R complexes and corresponding monomers calculated relative to the starting geometry (HADDOCK model, AlphaFold/AlphaFill predictions) together with average RMSF values of heavy atoms for each of the models. These metrics were obtained over the final 10 ns of three independent MD simulations, providing an estimate of both the overall structural deviations and the residue-level flexibility of the complexes. All values are reported in Å.

	RSAD2 – CYB5R3	***RSAD2-V1-CYB5R3***	***RSAD2-V2-CYB5R3***	***TtSAND – CYB5R3***	***RSAD2 – CYB5R1***	***RSAD2-CYB5R2***	***RSAD2-CYB5R4***
SAND-CYB5R							
**RMSD**<br>**RMSF**	1.44± 0.31<br>1.67 ±1.63	1.66 ± 0.33<br>1.63 ± 1.47	2.06 ± 0.45<br>1.95 ± 1.51	1.94 ± 0.42<br>1.91 ± 1.99	1.48± 0.35<br>2.24 ± 2.05	1.57 ± 0.45<br>1.44 ± 0.59	3.01 ± 0.72<br>1.50 ± 1.35
CYB5R							
RMSD	1.90 ±0.16<br>1.93 ±1.95	1.35 ± 0.09<br>1.67 ± 1.50	1.55 ± 0.18<br>2.00 ± 0.98	1.20 ± 0.14<br>1.82 ± 1.26	1.83 ± 0.12<br>2.14 ± 1.51	1.92 ± 0.49<br>1.43 ± 0.76	3.97 ± 0.33<br>1.52 ± 1.24
SAND							
**RMSD**	1.35 ±0.07<br>1.44 ±1.03	1.47 ± 0.07<br>1.60 ± 1.34	1.36 ± 0.07<br>1.89 ± 1.77	1.70 ± 0.14<br>1.96 ± 2.08	1.36 ± 0.07<br>2.33 ± 2.35	1.48 ± 0.07<br>1.42 ± 0.85	1.14 ± 0.42<br>1.48 ± 1.47

Table S6. Average electronic coupling, average steps and average constant electron transfer rate between the closest atom from the FAD structure and the closest atom from the [4Fe4S]-cluster. These values were obtained from the last 10 ns of MD.

	RSAD2 – CYB5R3	RSAD2-V1 – CYB5R3	RSAD2-V2 – CYB5R3	TtSAND – CYB5R3	RSAD2 – CYB5R1	RSAD2 – CYB5R2	RSAD2 – CYB5R4
**Average T_{DA}**	2.48E-06	9.79E-07	2.97E-09	3.81E-07	5.63E-06	5.95E-08	1.73E-07
**Average k_{ET} (s^{-1})**	1798.97	556.46	0.269	97.07	705.6	1.00	12.16

Average T_{DA}, average tunnelling matrix

Average k_{ET}, average electron transfer rate (s^{-1})

References

[1] Y. Sheng, Z. Chen, M. V Cherrier, L. Martin, T. T. T. Bui, W. Li, S. Lynham, Y. Nicolet, K. H. Ebrahimi, *Small* **2024**, *20*, 2310913.

[2] A. F. Alharbi, H. Kim, D. Chumroo, Y. JI, M. Hakil, K. H. Honarmand Ebrahimi, *Chem. Commun.* **2023**, *59*, 5419.

[3] K. H. Ebrahimi, J. Rowbotham, J. McCullagh, W. S. James, *ChemBioChem* **2020**, *21*, 1605.

[4] T. N. Hoang, M. Wu-Lu, A. Collauto, P.-L. Hagedoorn, M. Alexandru, M. Henschel, S. Kordasti, M. A. Mroginski, M. M. Roessler, K. H. Ebrahimi, *FEBS Lett.* **2025**, *599*, 952.

[5] K. H. Ebrahimi, J. Vowles, C. Browne, J. McCullagh, W. S. James, *FEBS Lett.* **2020**, *594*, 1631.

[6] R. Keller, K. Wuthrich, *Verl Goldau Cantina Switz* **2004**.

[7] Y. Shen, A. Bax, *J. Biomol. NMR* **2013**, *56*, 227.

[8] M. P. Williamson, *Prog. Nucl. Magn. Reson. Spectrosc.* **2013**, *73*, 1.

[9] J. Jumper, R. Evans, A. Pritzel, T. Green, M. Figurnov, O. Ronneberger, K. Tunyasuvunakool, R. Bates, A. Žídek, A. Potapenko, *Nature* **2021**, *596*, 583.

[10] M. L. Hekkelman, I. de Vries, R. P. Joosten, A. Perrakis, *bioRxiv* **2021**, 2021.11.26.470110.

[11] F. Autenrieth, E. Tajkhorshid, J. Baudry, Z. Luthey-Schulten, *J. Comput. Chem.* **2004**, *25*, 1613.

[12] D. M. A. Smith, Y. Xiong, T. P. Straatsma, K. M. Rosso, T. C. Squier, *J. Chem. Theory Comput.* **2012**, *8*, 2103.

[13] G. C. P. van Zundert, A. M. J. J. Bonvin, *Protein Struct. Predict.* **2014**, 163.

[14] M. T. Nelson, W. Humphrey, A. Gursoy, A. Dalke, L. V Kalé, R. D. Skeel, K. Schulten, *Int. J. Supercomput. Appl. High Perform. Comput.* **1996**, *10*, 251.

[15] J. Huang, A. D. MacKerell Jr, *J. Comput. Chem.* **2013**, *34*, 2135.

[16] R. B. Best, X. Zhu, J. Shim, P. E. M. Lopes, J. Mittal, M. Feig, A. D. MacKerell Jr, *J. Chem. Theory Comput.* **2012**, *8*, 3257.

[17] W. Humphrey, A. Dalke, K. Schulten, *J. Mol. Graph.* **1996**, *14*, 33.

[18] W. L. Jorgensen, J. Chandrasekhar, J. D. Madura, *J. Chem. Phys.* **1983**, *79*, 926.

[19] T. Meyer, E.-W. Knapp, *BBA-Bioenergetics* **2014**, e112.

[20] J.-P. Ryckaert, G. Ciccotti, H. J. C. Berendsen, *J. Comput. Phys.* **1977**, *23*, 327.

[21] T. Darden, D. York, L. Pedersen, *J. Chem. Phys.* **1993**, *98*, 10089.

[22] T. Darden, D. York, *DOI https//doi. org/10.1063/1.464397* **n.d.**, 10089.

[23] R. J. Gowers, M. Linke, J. Barnoud, T. J. E. Reddy, M. N. Melo, S. L. Seyler, J. Domanski, D. L. Dotson, S. Buchoux, I. M. Kenney, *MDAnalysis: A Python Package for the Rapid Analysis of Molecular Dynamics Simulations*, Los Alamos National Lab.(LANL), Los Alamos, NM (United States), **2019**.

[24] L. C. Xue, J. P. Rodrigues, P. L. Kastritis, A. M. Bonvin, A. Vangone, *Bioinformatics* **2016**, *32*, 3676.

[25] I. A. Balabin, X. Hu, D. N. Beratan, *J. Comput. Chem.* **2012**, *33*, 906.

[26] T. Utesch, M. A. Mroginski, *J. Phys. Chem. Lett.* **2010**, *1*, 2159.
